# Supplementary figures and images for: Long non-coding RNA NMRAL2P promotes glycolysis and reduces ROS in head and neck tumors by interacting with the ENO1 protein and promoting GPX2 transcription
Source: PeerJ. 2023 Oct 2;11:e16140. doi: 10.7717/peerj.16140 (PMC10552744; doi:10.7717/peerj.16140)

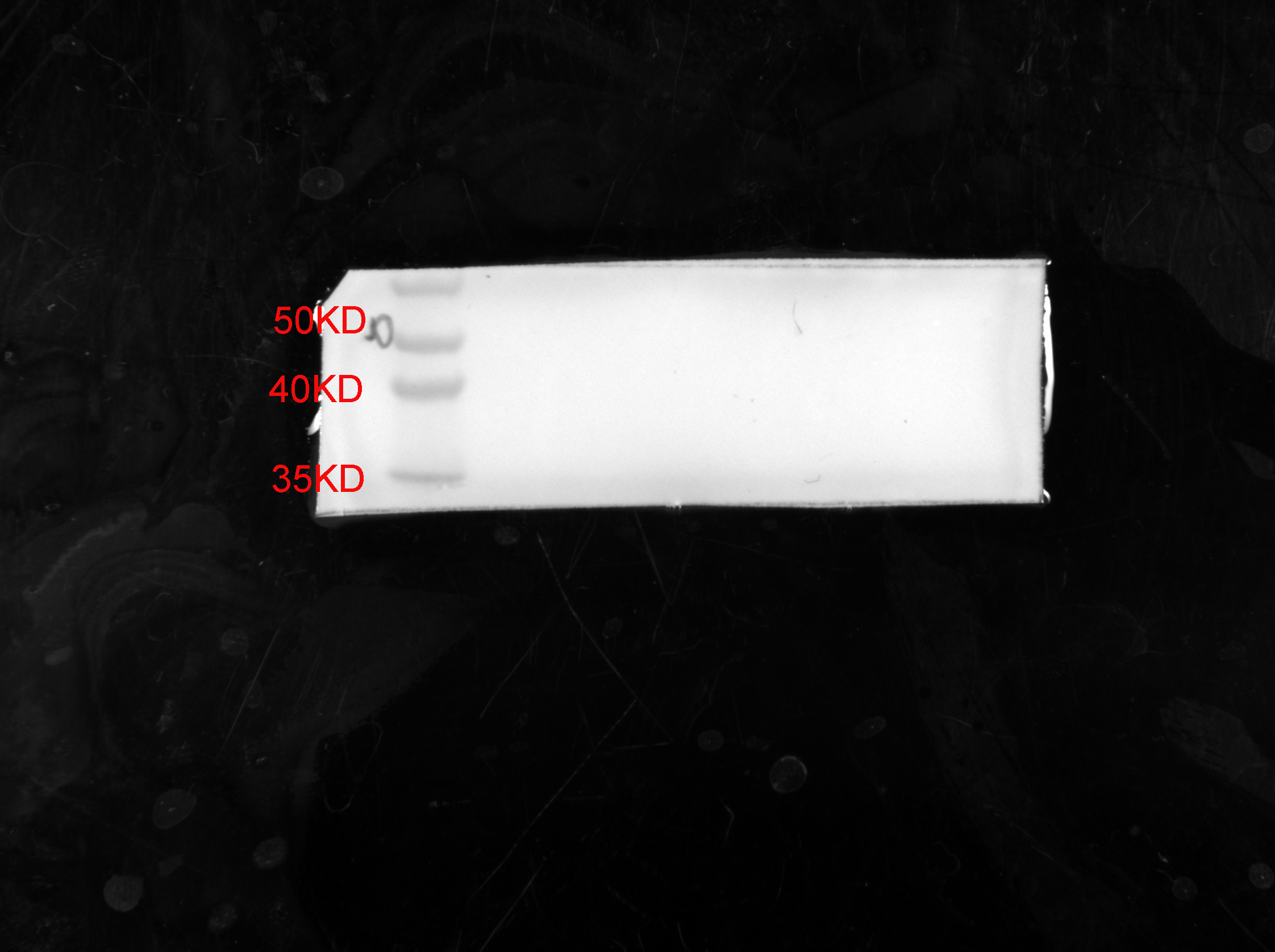

Supplement: Supplemental Information 1 — NMRAL2P overexpression plasmids (NMRAL2P-OE) or NMRAL2P knockdown (NMRAL2P-ASO) and their corresponding negative control groups (Vector or NC) were transferred into TU177 and AMC-HN-8 cells. After adding cycloheximide,proteins were collected at 0 h, 3 h, 6 h, 9 h and 12 h, respectively. Western blotting was used to detect the changes of ENO1 protein level to verify the effect of overexpression of NMRAL2P or knocking down NMRAL2P on ENO1 degradation. The protein blot images of ENO1. [file peerj-11-16140-s001.zip › ENO1 half-life/WB Verification of half-life of NMRAL2P overexpression/AMC-HN-8 a┬-actin NMRAL2P-oe White light_.png]

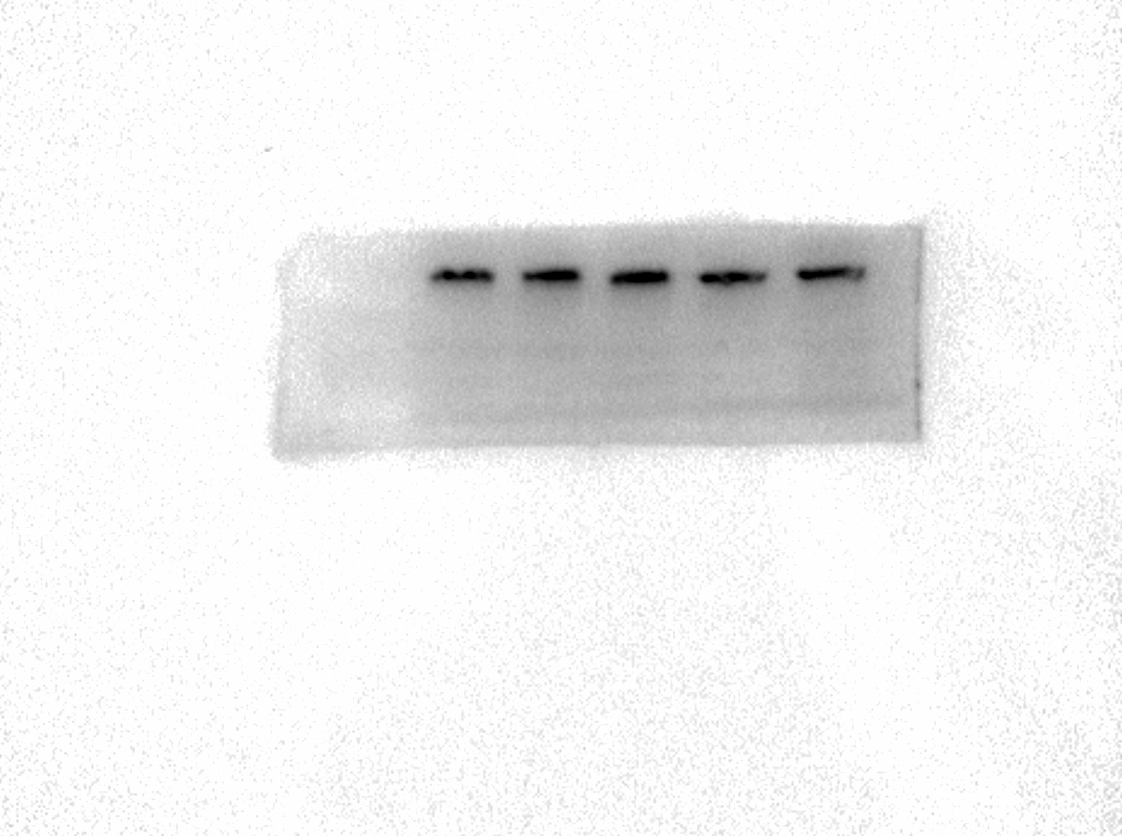

Supplement: Supplemental Information 1 — NMRAL2P overexpression plasmids (NMRAL2P-OE) or NMRAL2P knockdown (NMRAL2P-ASO) and their corresponding negative control groups (Vector or NC) were transferred into TU177 and AMC-HN-8 cells. After adding cycloheximide,proteins were collected at 0 h, 3 h, 6 h, 9 h and 12 h, respectively. Western blotting was used to detect the changes of ENO1 protein level to verify the effect of overexpression of NMRAL2P or knocking down NMRAL2P on ENO1 degradation. The protein blot images of ENO1. [file peerj-11-16140-s001.zip › ENO1 half-life/WB Verification of half-life of NMRAL2P overexpression/AMC-HN-8 a┬-actin NMRAL2P-oe.png]

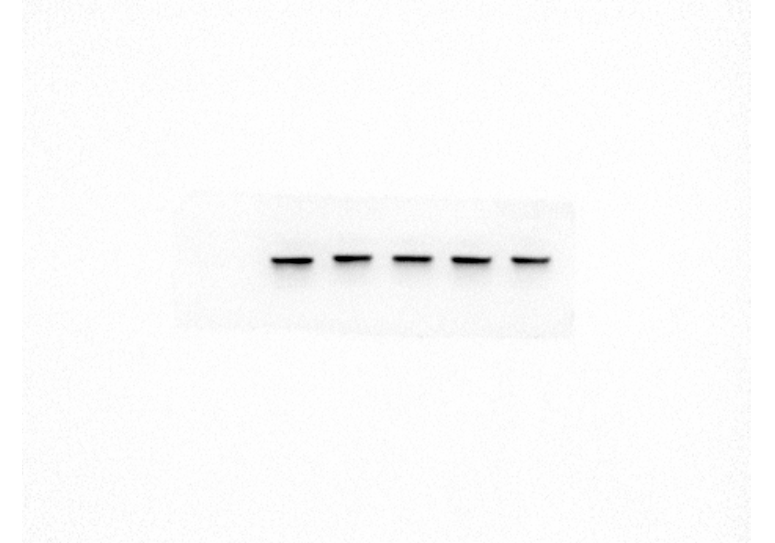

Supplement: Supplemental Information 1 — NMRAL2P overexpression plasmids (NMRAL2P-OE) or NMRAL2P knockdown (NMRAL2P-ASO) and their corresponding negative control groups (Vector or NC) were transferred into TU177 and AMC-HN-8 cells. After adding cycloheximide,proteins were collected at 0 h, 3 h, 6 h, 9 h and 12 h, respectively. Western blotting was used to detect the changes of ENO1 protein level to verify the effect of overexpression of NMRAL2P or knocking down NMRAL2P on ENO1 degradation. The protein blot images of ENO1. [file peerj-11-16140-s001.zip › ENO1 half-life/WB Verification of half-life of NMRAL2P overexpression/AMC-HN-8 a┬-actin Vector_.png]

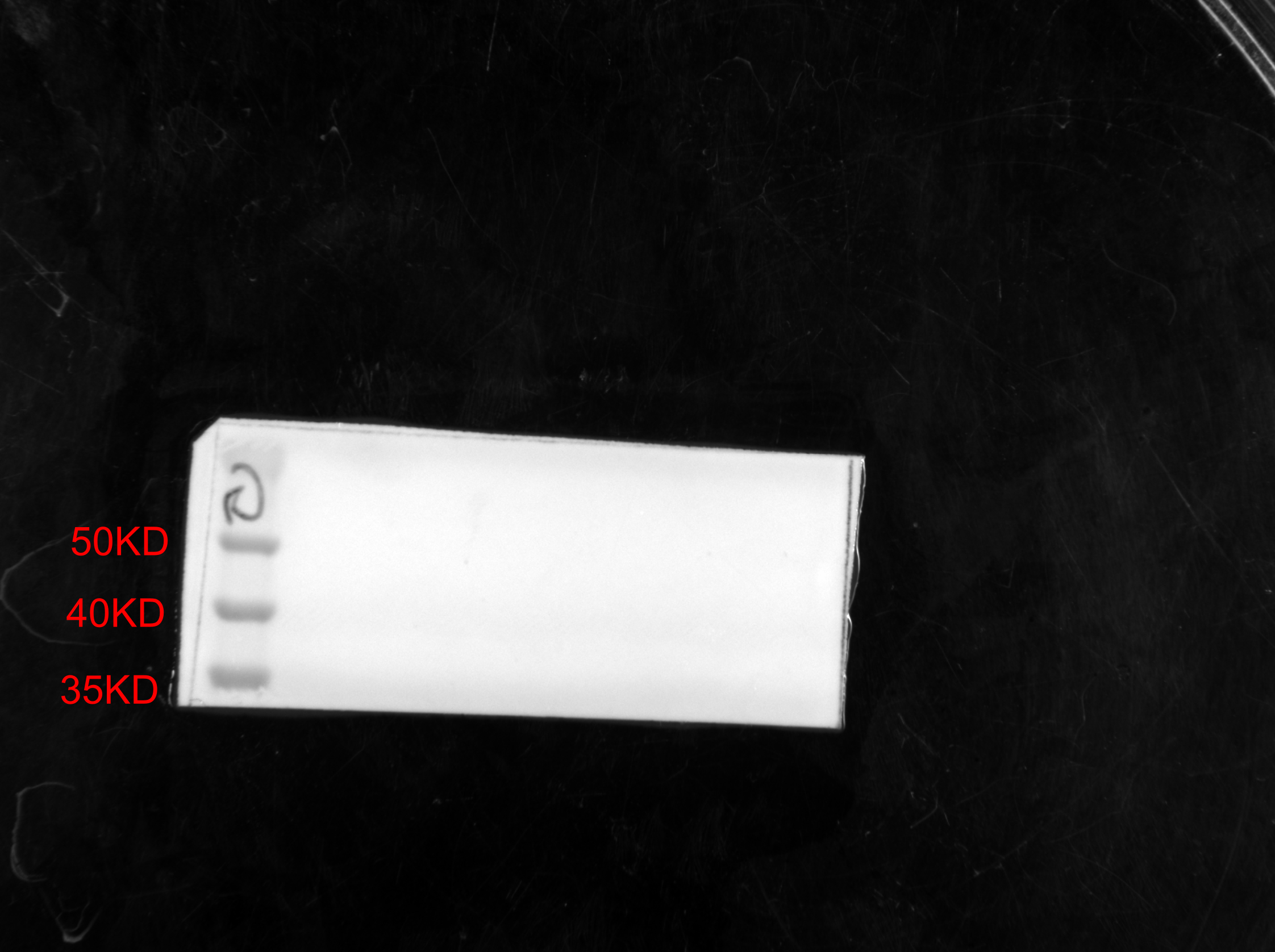

Supplement: Supplemental Information 1 — NMRAL2P overexpression plasmids (NMRAL2P-OE) or NMRAL2P knockdown (NMRAL2P-ASO) and their corresponding negative control groups (Vector or NC) were transferred into TU177 and AMC-HN-8 cells. After adding cycloheximide,proteins were collected at 0 h, 3 h, 6 h, 9 h and 12 h, respectively. Western blotting was used to detect the changes of ENO1 protein level to verify the effect of overexpression of NMRAL2P or knocking down NMRAL2P on ENO1 degradation. The protein blot images of ENO1. [file peerj-11-16140-s001.zip › ENO1 half-life/WB Verification of half-life of NMRAL2P overexpression/AMC-HN-8 ENO1 NMRAL2P-oe White light.png]

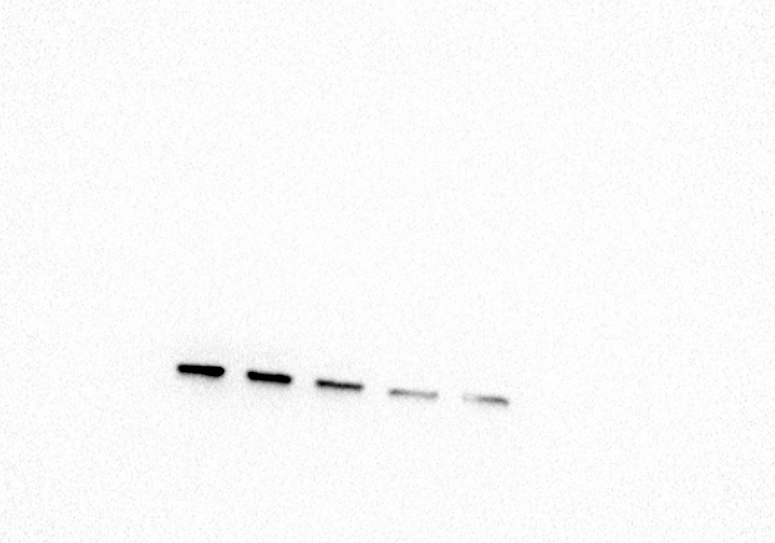

Supplement: Supplemental Information 1 — NMRAL2P overexpression plasmids (NMRAL2P-OE) or NMRAL2P knockdown (NMRAL2P-ASO) and their corresponding negative control groups (Vector or NC) were transferred into TU177 and AMC-HN-8 cells. After adding cycloheximide,proteins were collected at 0 h, 3 h, 6 h, 9 h and 12 h, respectively. Western blotting was used to detect the changes of ENO1 protein level to verify the effect of overexpression of NMRAL2P or knocking down NMRAL2P on ENO1 degradation. The protein blot images of ENO1. [file peerj-11-16140-s001.zip › ENO1 half-life/WB Verification of half-life of NMRAL2P overexpression/AMC-HN-8 ENO1 NMRAL2P-oe.png]

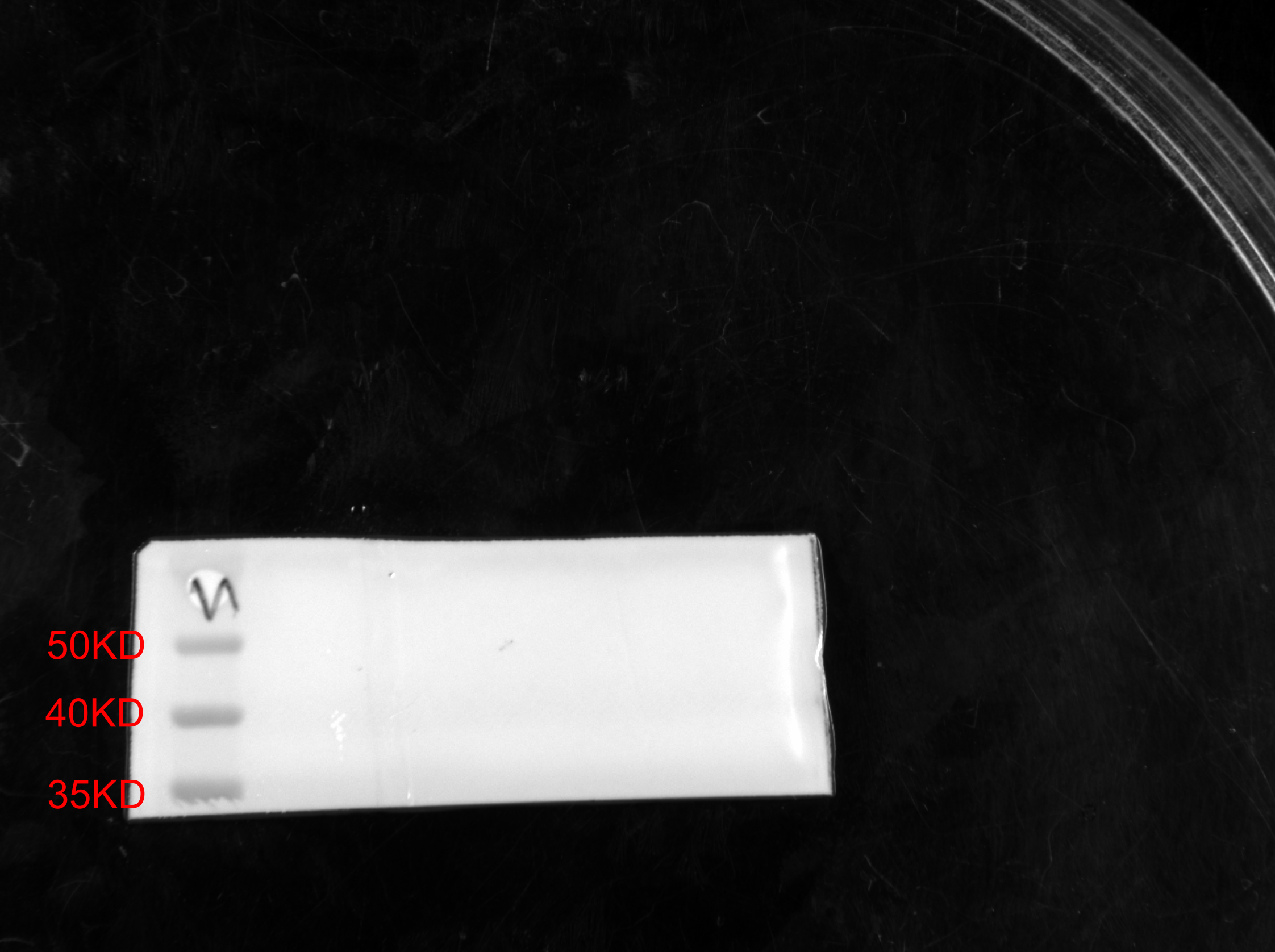

Supplement: Supplemental Information 1 — NMRAL2P overexpression plasmids (NMRAL2P-OE) or NMRAL2P knockdown (NMRAL2P-ASO) and their corresponding negative control groups (Vector or NC) were transferred into TU177 and AMC-HN-8 cells. After adding cycloheximide,proteins were collected at 0 h, 3 h, 6 h, 9 h and 12 h, respectively. Western blotting was used to detect the changes of ENO1 protein level to verify the effect of overexpression of NMRAL2P or knocking down NMRAL2P on ENO1 degradation. The protein blot images of ENO1. [file peerj-11-16140-s001.zip › ENO1 half-life/WB Verification of half-life of NMRAL2P overexpression/AMC-HN-8 ENO1 Vector White light.png]

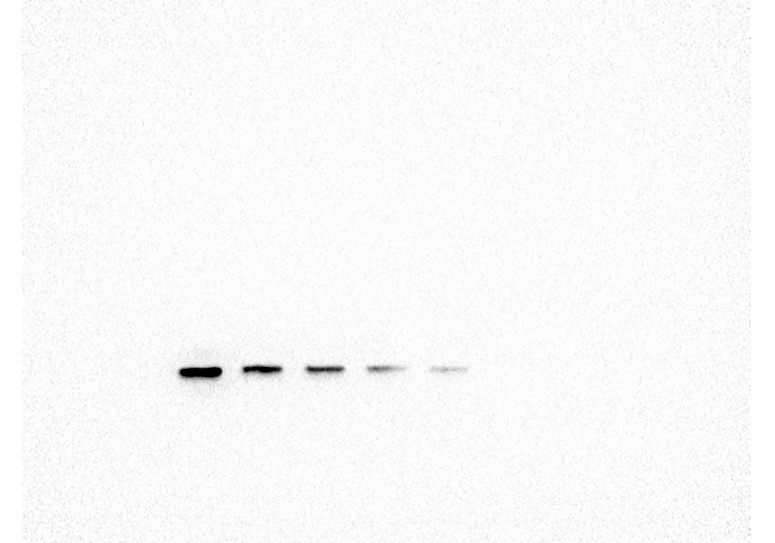

Supplement: Supplemental Information 1 — NMRAL2P overexpression plasmids (NMRAL2P-OE) or NMRAL2P knockdown (NMRAL2P-ASO) and their corresponding negative control groups (Vector or NC) were transferred into TU177 and AMC-HN-8 cells. After adding cycloheximide,proteins were collected at 0 h, 3 h, 6 h, 9 h and 12 h, respectively. Western blotting was used to detect the changes of ENO1 protein level to verify the effect of overexpression of NMRAL2P or knocking down NMRAL2P on ENO1 degradation. The protein blot images of ENO1. [file peerj-11-16140-s001.zip › ENO1 half-life/WB Verification of half-life of NMRAL2P overexpression/AMC-HN-8 ENO1 Vector.png]

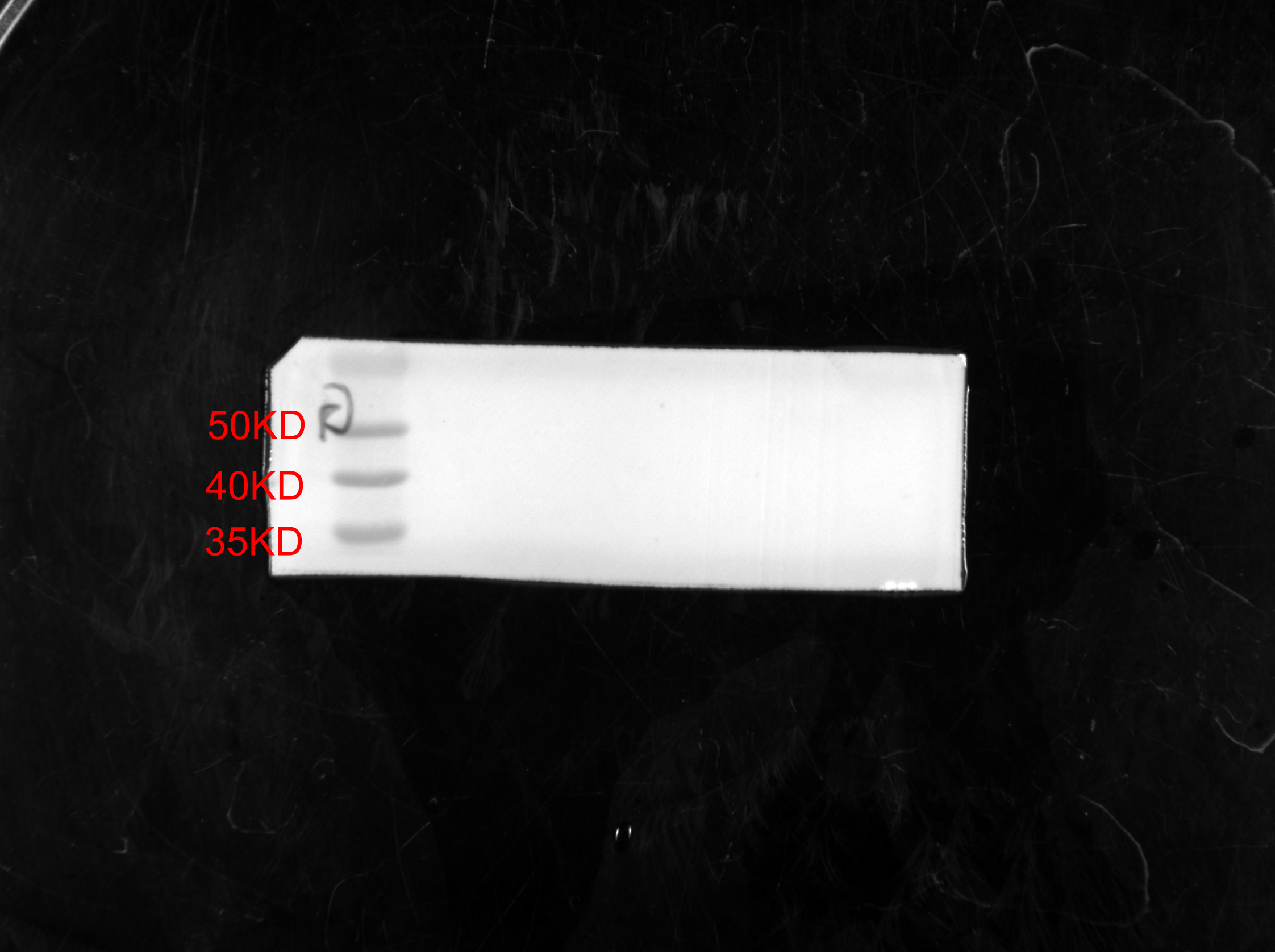

Supplement: Supplemental Information 1 — NMRAL2P overexpression plasmids (NMRAL2P-OE) or NMRAL2P knockdown (NMRAL2P-ASO) and their corresponding negative control groups (Vector or NC) were transferred into TU177 and AMC-HN-8 cells. After adding cycloheximide,proteins were collected at 0 h, 3 h, 6 h, 9 h and 12 h, respectively. Western blotting was used to detect the changes of ENO1 protein level to verify the effect of overexpression of NMRAL2P or knocking down NMRAL2P on ENO1 degradation. The protein blot images of ENO1. [file peerj-11-16140-s001.zip › ENO1 half-life/WB Verification of half-life of NMRAL2P overexpression/AMC-HN-8 a┬-actin Vector White light.png]

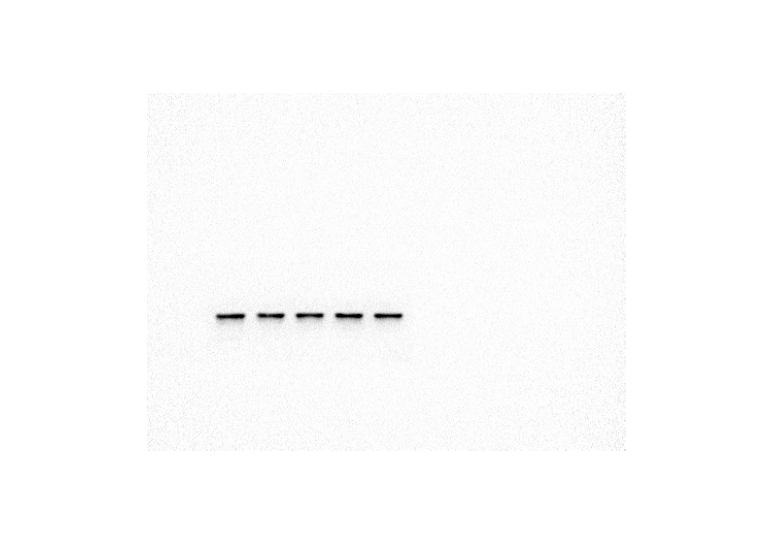

Supplement: Supplemental Information 1 — NMRAL2P overexpression plasmids (NMRAL2P-OE) or NMRAL2P knockdown (NMRAL2P-ASO) and their corresponding negative control groups (Vector or NC) were transferred into TU177 and AMC-HN-8 cells. After adding cycloheximide,proteins were collected at 0 h, 3 h, 6 h, 9 h and 12 h, respectively. Western blotting was used to detect the changes of ENO1 protein level to verify the effect of overexpression of NMRAL2P or knocking down NMRAL2P on ENO1 degradation. The protein blot images of ENO1. [file peerj-11-16140-s001.zip › ENO1 half-life/WB Verification of half-life of NMRAL2P overexpression/TU177 a┬-actin NMRAL2P-oe.png]

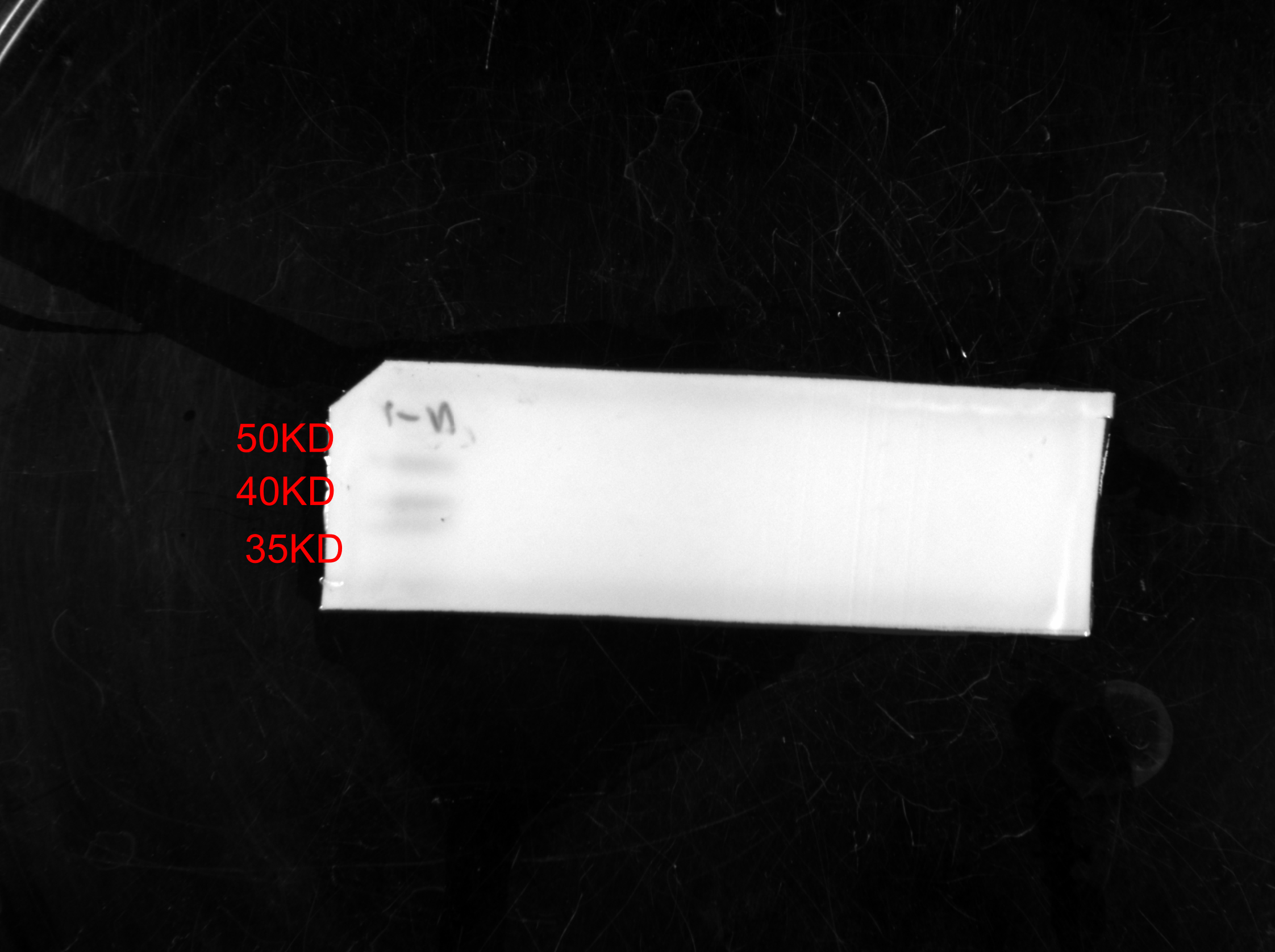

Supplement: Supplemental Information 1 — NMRAL2P overexpression plasmids (NMRAL2P-OE) or NMRAL2P knockdown (NMRAL2P-ASO) and their corresponding negative control groups (Vector or NC) were transferred into TU177 and AMC-HN-8 cells. After adding cycloheximide,proteins were collected at 0 h, 3 h, 6 h, 9 h and 12 h, respectively. Western blotting was used to detect the changes of ENO1 protein level to verify the effect of overexpression of NMRAL2P or knocking down NMRAL2P on ENO1 degradation. The protein blot images of ENO1. [file peerj-11-16140-s001.zip › ENO1 half-life/WB Verification of half-life of NMRAL2P overexpression/TU177 ENO1 NMRAL2P- oe White light.png]

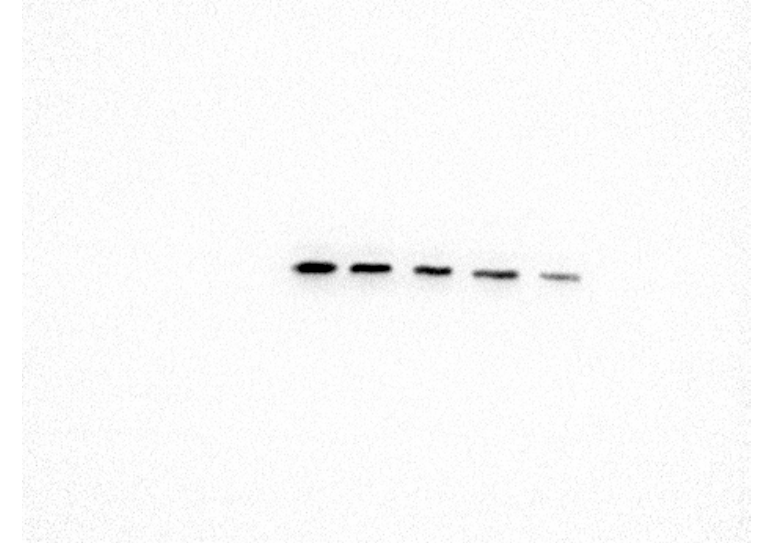

Supplement: Supplemental Information 1 — NMRAL2P overexpression plasmids (NMRAL2P-OE) or NMRAL2P knockdown (NMRAL2P-ASO) and their corresponding negative control groups (Vector or NC) were transferred into TU177 and AMC-HN-8 cells. After adding cycloheximide,proteins were collected at 0 h, 3 h, 6 h, 9 h and 12 h, respectively. Western blotting was used to detect the changes of ENO1 protein level to verify the effect of overexpression of NMRAL2P or knocking down NMRAL2P on ENO1 degradation. The protein blot images of ENO1. [file peerj-11-16140-s001.zip › ENO1 half-life/WB Verification of half-life of NMRAL2P overexpression/TU177 ENO1 NMRAL2P-oe_.png]

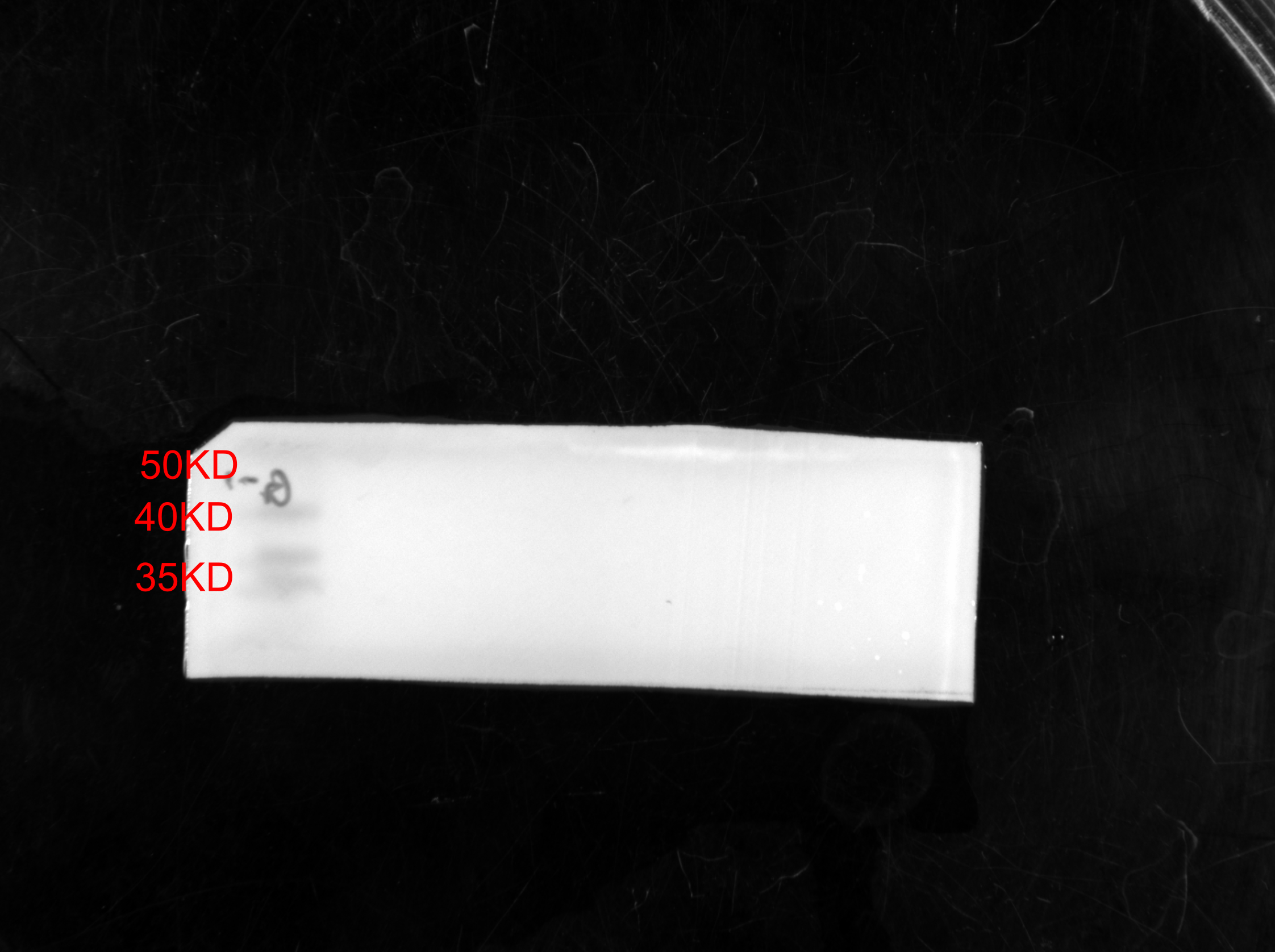

Supplement: Supplemental Information 1 — NMRAL2P overexpression plasmids (NMRAL2P-OE) or NMRAL2P knockdown (NMRAL2P-ASO) and their corresponding negative control groups (Vector or NC) were transferred into TU177 and AMC-HN-8 cells. After adding cycloheximide,proteins were collected at 0 h, 3 h, 6 h, 9 h and 12 h, respectively. Western blotting was used to detect the changes of ENO1 protein level to verify the effect of overexpression of NMRAL2P or knocking down NMRAL2P on ENO1 degradation. The protein blot images of ENO1. [file peerj-11-16140-s001.zip › ENO1 half-life/WB Verification of half-life of NMRAL2P overexpression/TU177 ENO1 vector White light_.png]

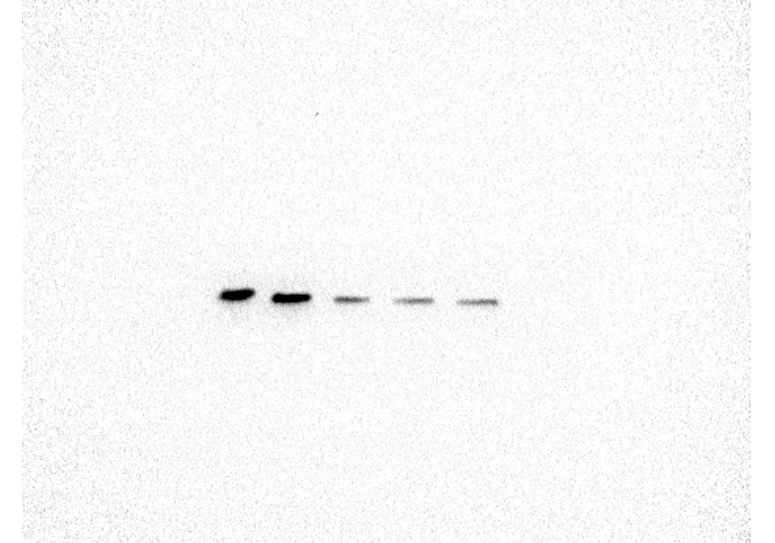

Supplement: Supplemental Information 1 — NMRAL2P overexpression plasmids (NMRAL2P-OE) or NMRAL2P knockdown (NMRAL2P-ASO) and their corresponding negative control groups (Vector or NC) were transferred into TU177 and AMC-HN-8 cells. After adding cycloheximide,proteins were collected at 0 h, 3 h, 6 h, 9 h and 12 h, respectively. Western blotting was used to detect the changes of ENO1 protein level to verify the effect of overexpression of NMRAL2P or knocking down NMRAL2P on ENO1 degradation. The protein blot images of ENO1. [file peerj-11-16140-s001.zip › ENO1 half-life/WB Verification of half-life of NMRAL2P overexpression/TU177 ENO1 Vector.png]

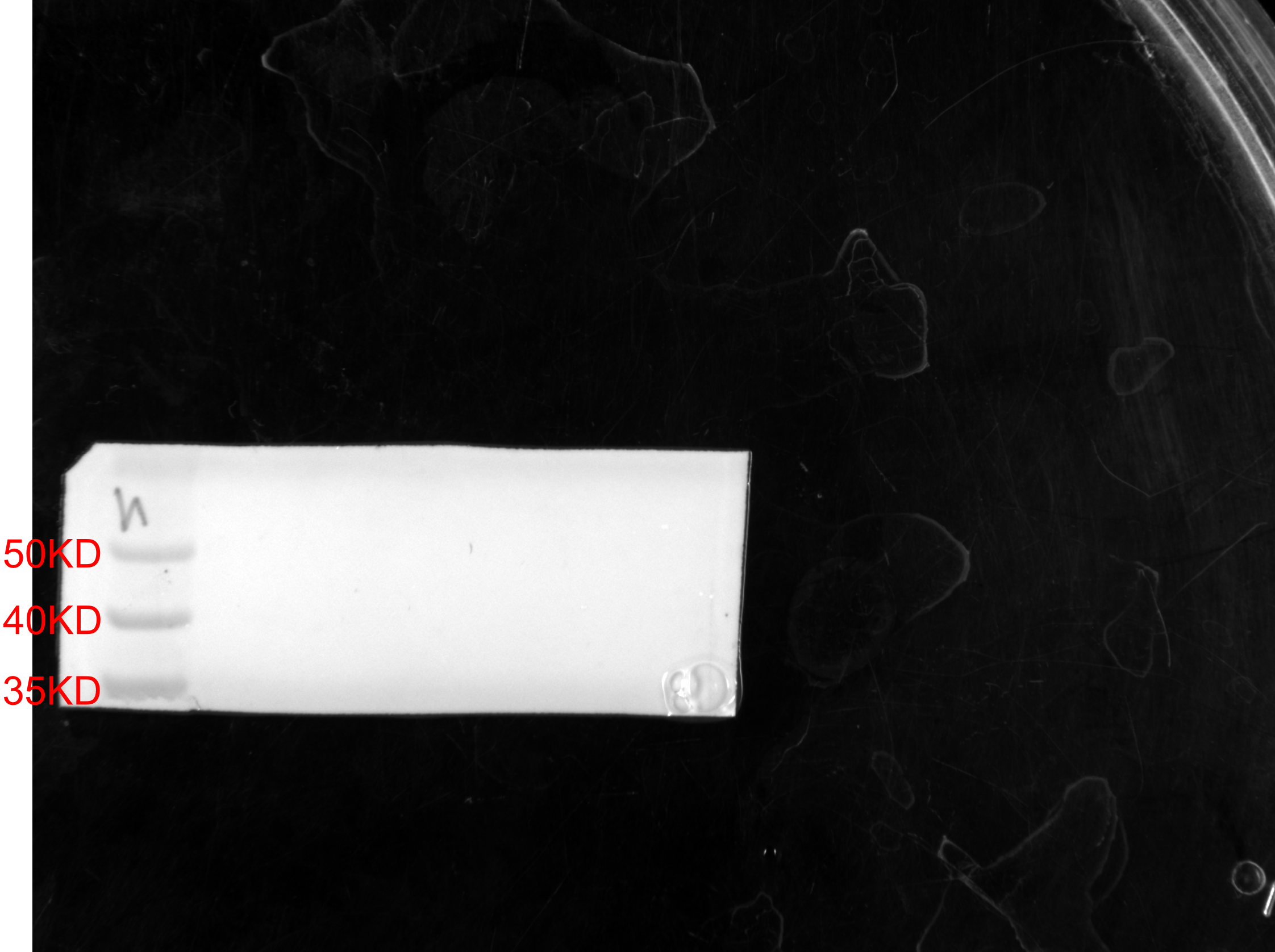

Supplement: Supplemental Information 1 — NMRAL2P overexpression plasmids (NMRAL2P-OE) or NMRAL2P knockdown (NMRAL2P-ASO) and their corresponding negative control groups (Vector or NC) were transferred into TU177 and AMC-HN-8 cells. After adding cycloheximide,proteins were collected at 0 h, 3 h, 6 h, 9 h and 12 h, respectively. Western blotting was used to detect the changes of ENO1 protein level to verify the effect of overexpression of NMRAL2P or knocking down NMRAL2P on ENO1 degradation. The protein blot images of ENO1. [file peerj-11-16140-s001.zip › ENO1 half-life/WB Verification of half-life of NMRAL2P overexpression/TU177 a┬-actin NMRAL2P-oe White light_.png]

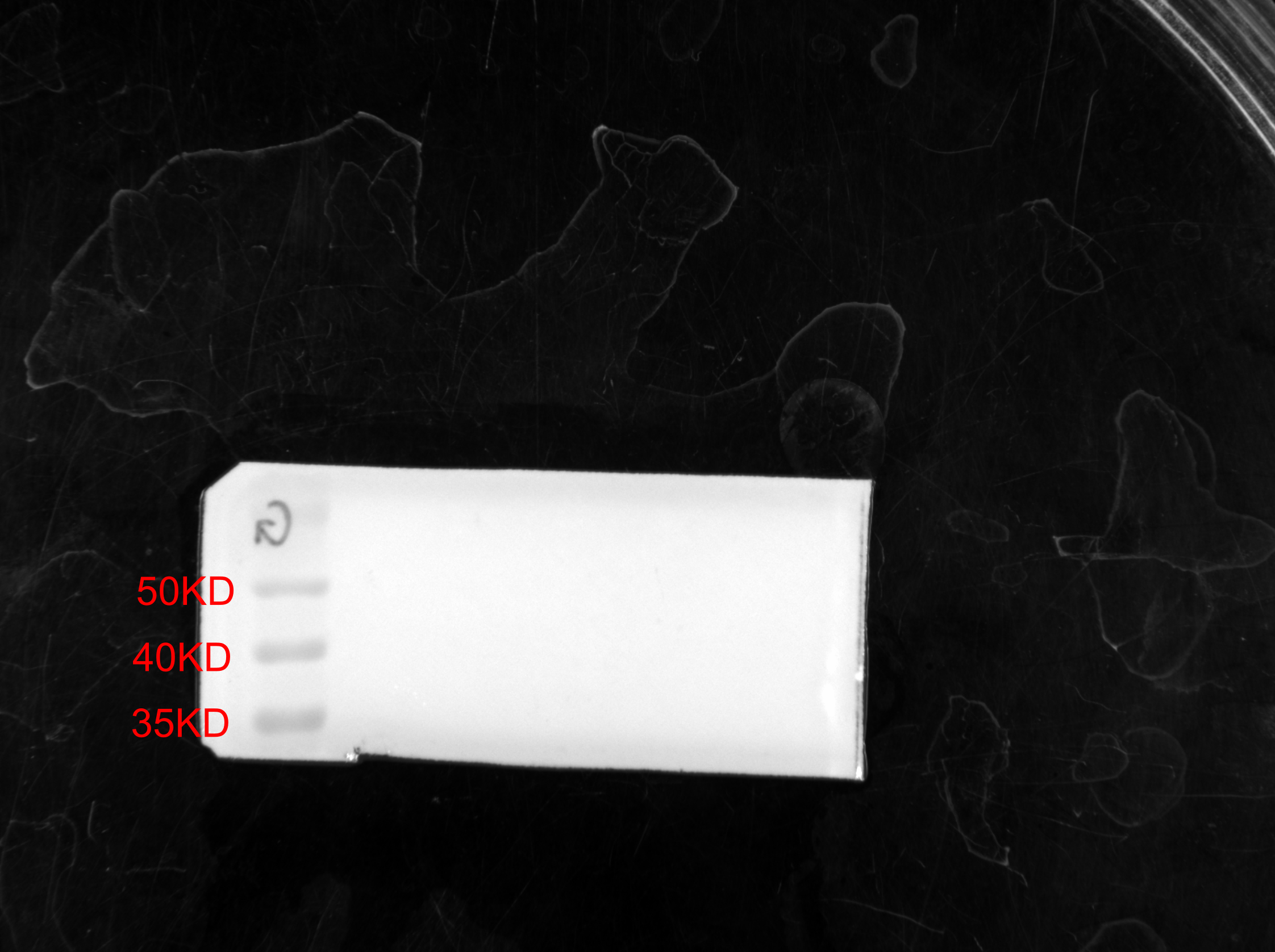

Supplement: Supplemental Information 1 — NMRAL2P overexpression plasmids (NMRAL2P-OE) or NMRAL2P knockdown (NMRAL2P-ASO) and their corresponding negative control groups (Vector or NC) were transferred into TU177 and AMC-HN-8 cells. After adding cycloheximide,proteins were collected at 0 h, 3 h, 6 h, 9 h and 12 h, respectively. Western blotting was used to detect the changes of ENO1 protein level to verify the effect of overexpression of NMRAL2P or knocking down NMRAL2P on ENO1 degradation. The protein blot images of ENO1. [file peerj-11-16140-s001.zip › ENO1 half-life/WB Verification of half-life of NMRAL2P overexpression/TU177 a┬-actin Vector White light_.png]

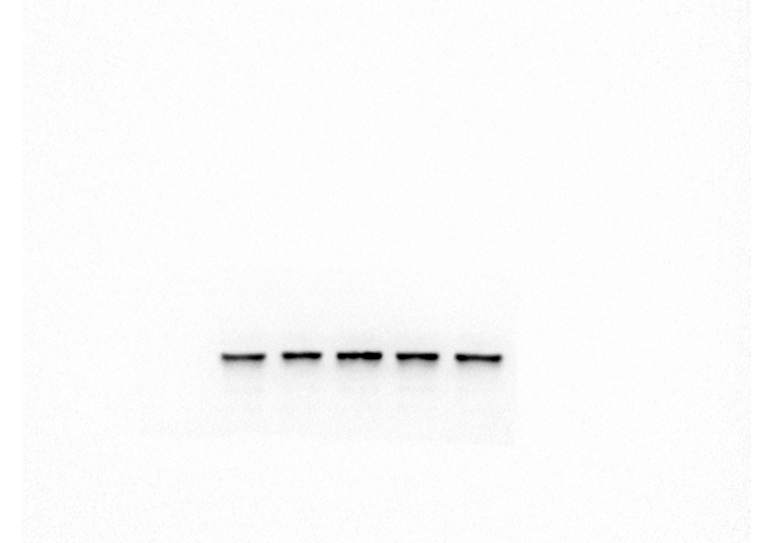

Supplement: Supplemental Information 1 — NMRAL2P overexpression plasmids (NMRAL2P-OE) or NMRAL2P knockdown (NMRAL2P-ASO) and their corresponding negative control groups (Vector or NC) were transferred into TU177 and AMC-HN-8 cells. After adding cycloheximide,proteins were collected at 0 h, 3 h, 6 h, 9 h and 12 h, respectively. Western blotting was used to detect the changes of ENO1 protein level to verify the effect of overexpression of NMRAL2P or knocking down NMRAL2P on ENO1 degradation. The protein blot images of ENO1. [file peerj-11-16140-s001.zip › ENO1 half-life/WB Verification of half-life of NMRAL2P overexpression/TU177 a┬-actin Vector.png]

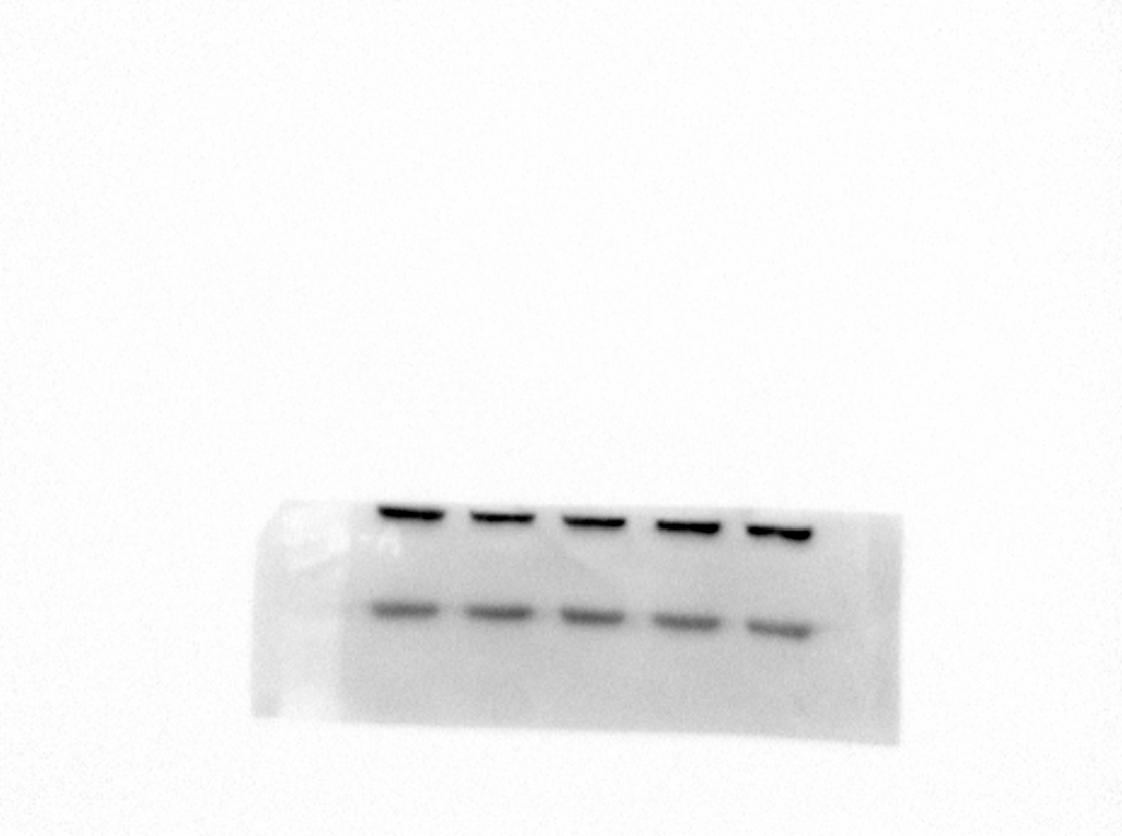

Supplement: Supplemental Information 1 — NMRAL2P overexpression plasmids (NMRAL2P-OE) or NMRAL2P knockdown (NMRAL2P-ASO) and their corresponding negative control groups (Vector or NC) were transferred into TU177 and AMC-HN-8 cells. After adding cycloheximide,proteins were collected at 0 h, 3 h, 6 h, 9 h and 12 h, respectively. Western blotting was used to detect the changes of ENO1 protein level to verify the effect of overexpression of NMRAL2P or knocking down NMRAL2P on ENO1 degradation. The protein blot images of ENO1. [file peerj-11-16140-s001.zip › ENO1 half-life/WB Verification of NMRAL2P knock-down half-life/AMC-HN-8 ENO1 NC Exposure_8.0sec.png]

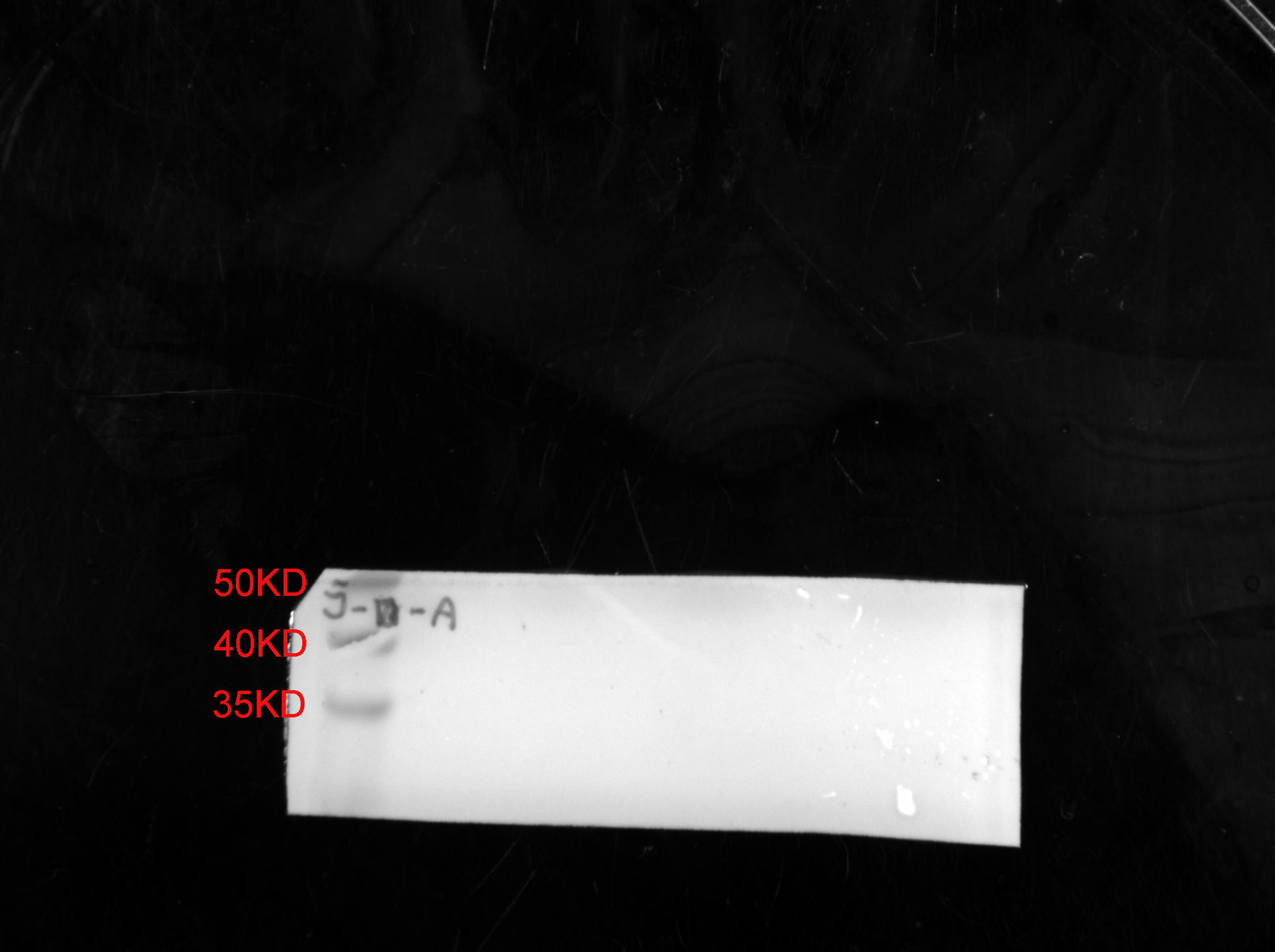

Supplement: Supplemental Information 1 — NMRAL2P overexpression plasmids (NMRAL2P-OE) or NMRAL2P knockdown (NMRAL2P-ASO) and their corresponding negative control groups (Vector or NC) were transferred into TU177 and AMC-HN-8 cells. After adding cycloheximide,proteins were collected at 0 h, 3 h, 6 h, 9 h and 12 h, respectively. Western blotting was used to detect the changes of ENO1 protein level to verify the effect of overexpression of NMRAL2P or knocking down NMRAL2P on ENO1 degradation. The protein blot images of ENO1. [file peerj-11-16140-s001.zip › ENO1 half-life/WB Verification of NMRAL2P knock-down half-life/AMC-HN-8 ENO1 NC White light.png]

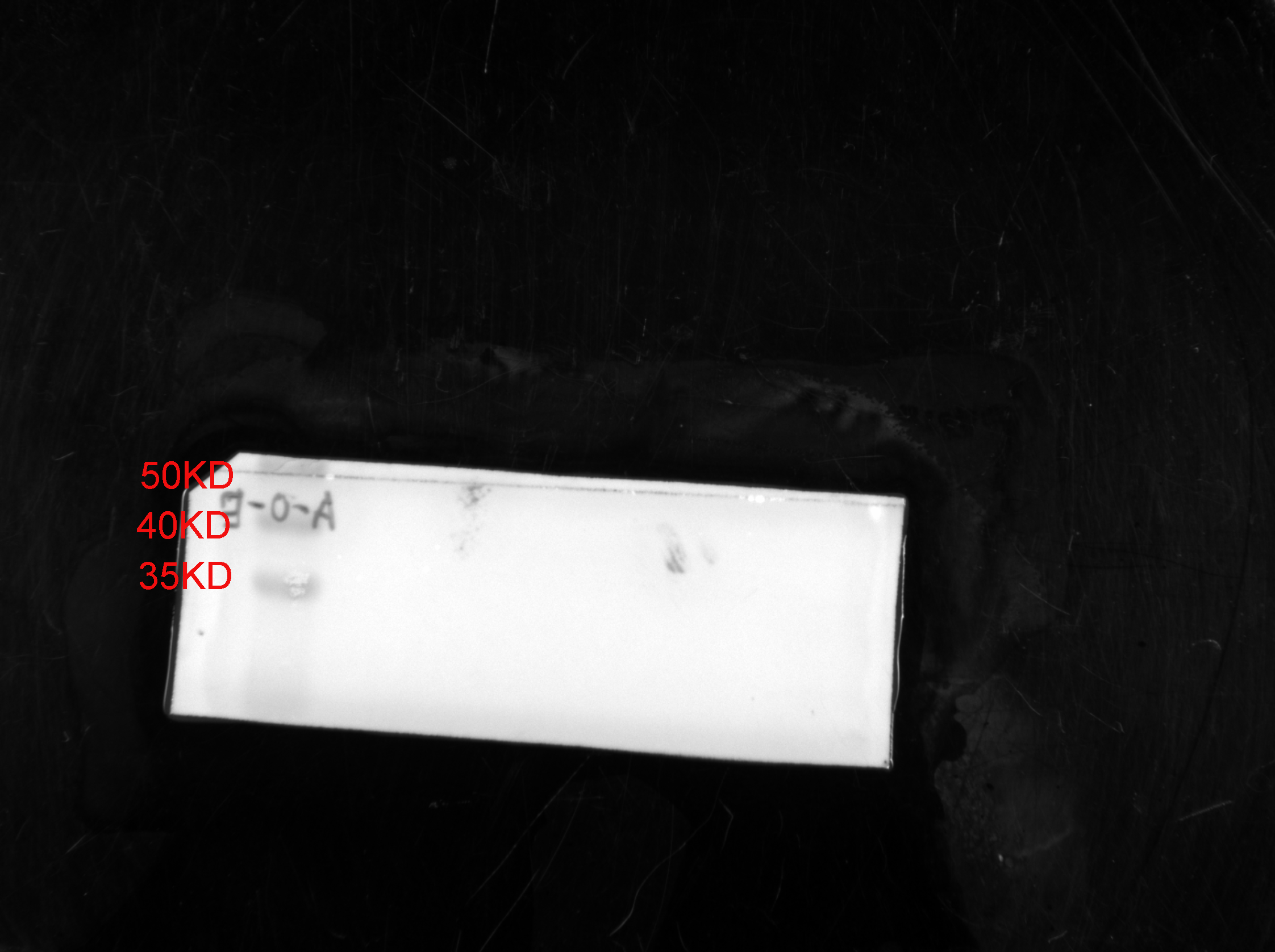

Supplement: Supplemental Information 1 — NMRAL2P overexpression plasmids (NMRAL2P-OE) or NMRAL2P knockdown (NMRAL2P-ASO) and their corresponding negative control groups (Vector or NC) were transferred into TU177 and AMC-HN-8 cells. After adding cycloheximide,proteins were collected at 0 h, 3 h, 6 h, 9 h and 12 h, respectively. Western blotting was used to detect the changes of ENO1 protein level to verify the effect of overexpression of NMRAL2P or knocking down NMRAL2P on ENO1 degradation. The protein blot images of ENO1. [file peerj-11-16140-s001.zip › ENO1 half-life/WB Verification of NMRAL2P knock-down half-life/AMC-HN-8 ENO1 NMRAL2P-ASO White light.png]

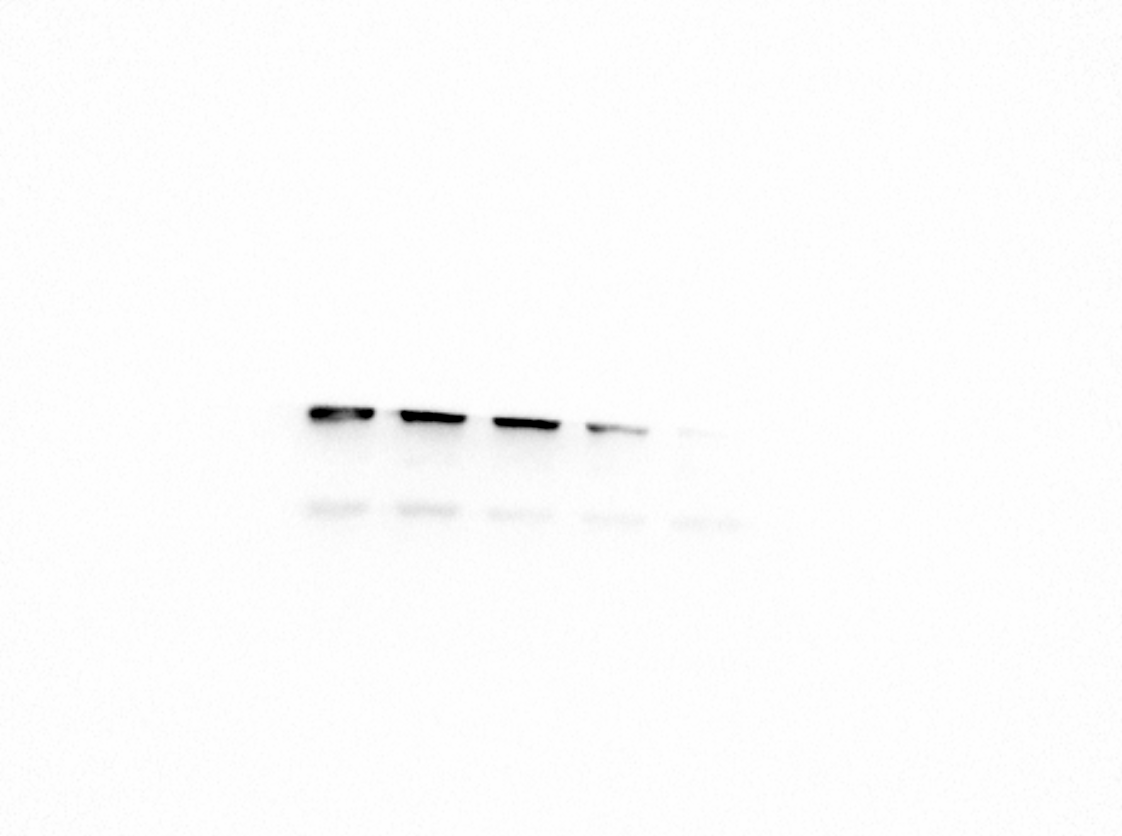

Supplement: Supplemental Information 1 — NMRAL2P overexpression plasmids (NMRAL2P-OE) or NMRAL2P knockdown (NMRAL2P-ASO) and their corresponding negative control groups (Vector or NC) were transferred into TU177 and AMC-HN-8 cells. After adding cycloheximide,proteins were collected at 0 h, 3 h, 6 h, 9 h and 12 h, respectively. Western blotting was used to detect the changes of ENO1 protein level to verify the effect of overexpression of NMRAL2P or knocking down NMRAL2P on ENO1 degradation. The protein blot images of ENO1. [file peerj-11-16140-s001.zip › ENO1 half-life/WB Verification of NMRAL2P knock-down half-life/AMC-HN-8 ENO1 NMRAL2P-ASO_Exposure_1.0sec.png]

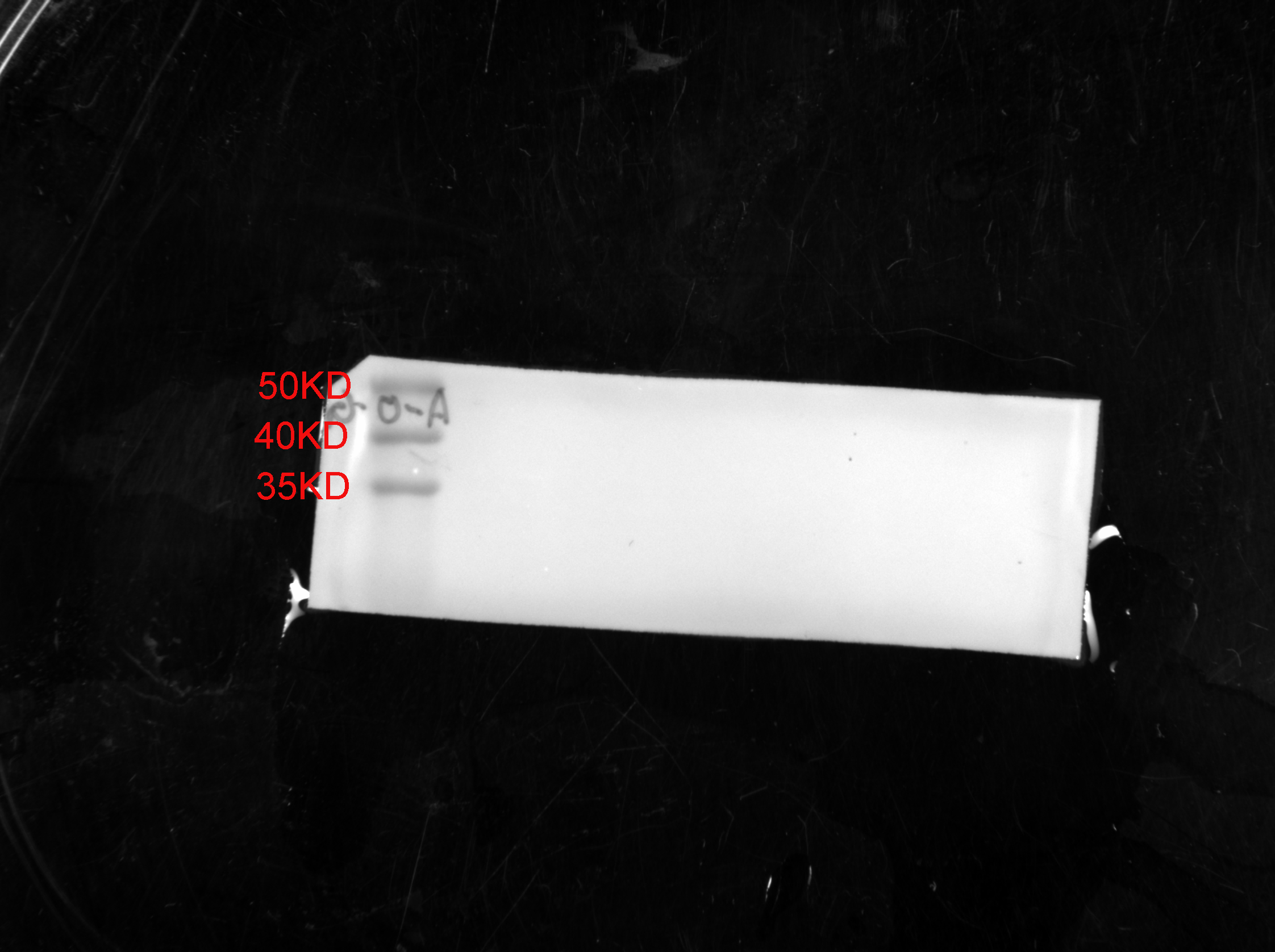

Supplement: Supplemental Information 1 — NMRAL2P overexpression plasmids (NMRAL2P-OE) or NMRAL2P knockdown (NMRAL2P-ASO) and their corresponding negative control groups (Vector or NC) were transferred into TU177 and AMC-HN-8 cells. After adding cycloheximide,proteins were collected at 0 h, 3 h, 6 h, 9 h and 12 h, respectively. Western blotting was used to detect the changes of ENO1 protein level to verify the effect of overexpression of NMRAL2P or knocking down NMRAL2P on ENO1 degradation. The protein blot images of ENO1. [file peerj-11-16140-s001.zip › ENO1 half-life/WB Verification of NMRAL2P knock-down half-life/AMC-HN-8 a┬-actin NC White light.png]

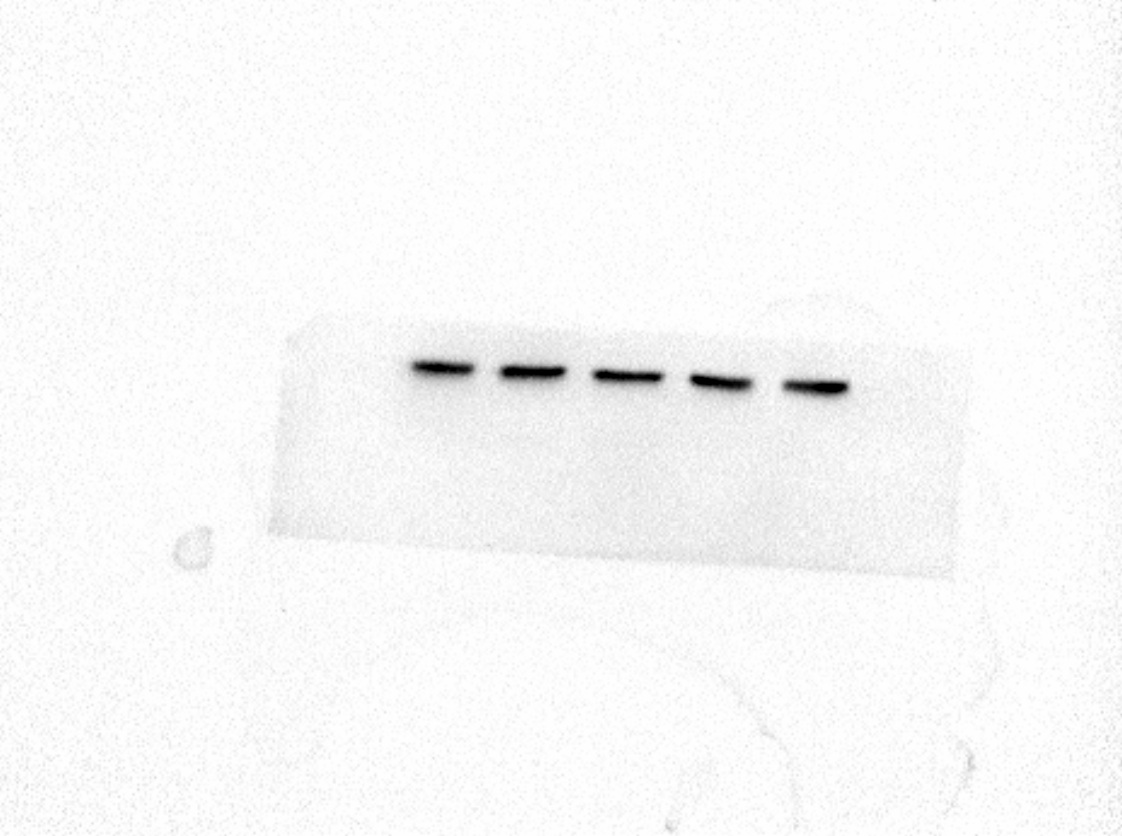

Supplement: Supplemental Information 1 — NMRAL2P overexpression plasmids (NMRAL2P-OE) or NMRAL2P knockdown (NMRAL2P-ASO) and their corresponding negative control groups (Vector or NC) were transferred into TU177 and AMC-HN-8 cells. After adding cycloheximide,proteins were collected at 0 h, 3 h, 6 h, 9 h and 12 h, respectively. Western blotting was used to detect the changes of ENO1 protein level to verify the effect of overexpression of NMRAL2P or knocking down NMRAL2P on ENO1 degradation. The protein blot images of ENO1. [file peerj-11-16140-s001.zip › ENO1 half-life/WB Verification of NMRAL2P knock-down half-life/AMC-HN-8 a┬-actin NC_Exposure_70.0sec.png]

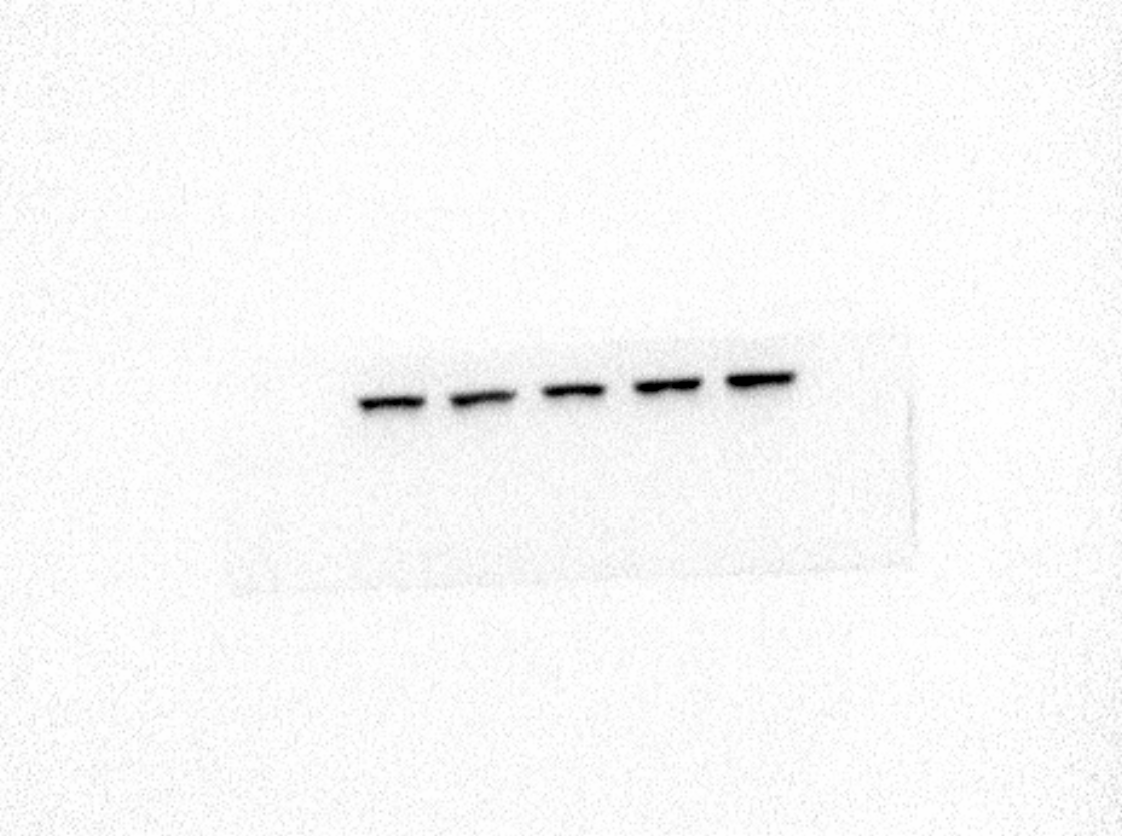

Supplement: Supplemental Information 1 — NMRAL2P overexpression plasmids (NMRAL2P-OE) or NMRAL2P knockdown (NMRAL2P-ASO) and their corresponding negative control groups (Vector or NC) were transferred into TU177 and AMC-HN-8 cells. After adding cycloheximide,proteins were collected at 0 h, 3 h, 6 h, 9 h and 12 h, respectively. Western blotting was used to detect the changes of ENO1 protein level to verify the effect of overexpression of NMRAL2P or knocking down NMRAL2P on ENO1 degradation. The protein blot images of ENO1. [file peerj-11-16140-s001.zip › ENO1 half-life/WB Verification of NMRAL2P knock-down half-life/AMC-HN-8 a┬-actin NMRAL2P-ASO Exposure_13.3sec.png]

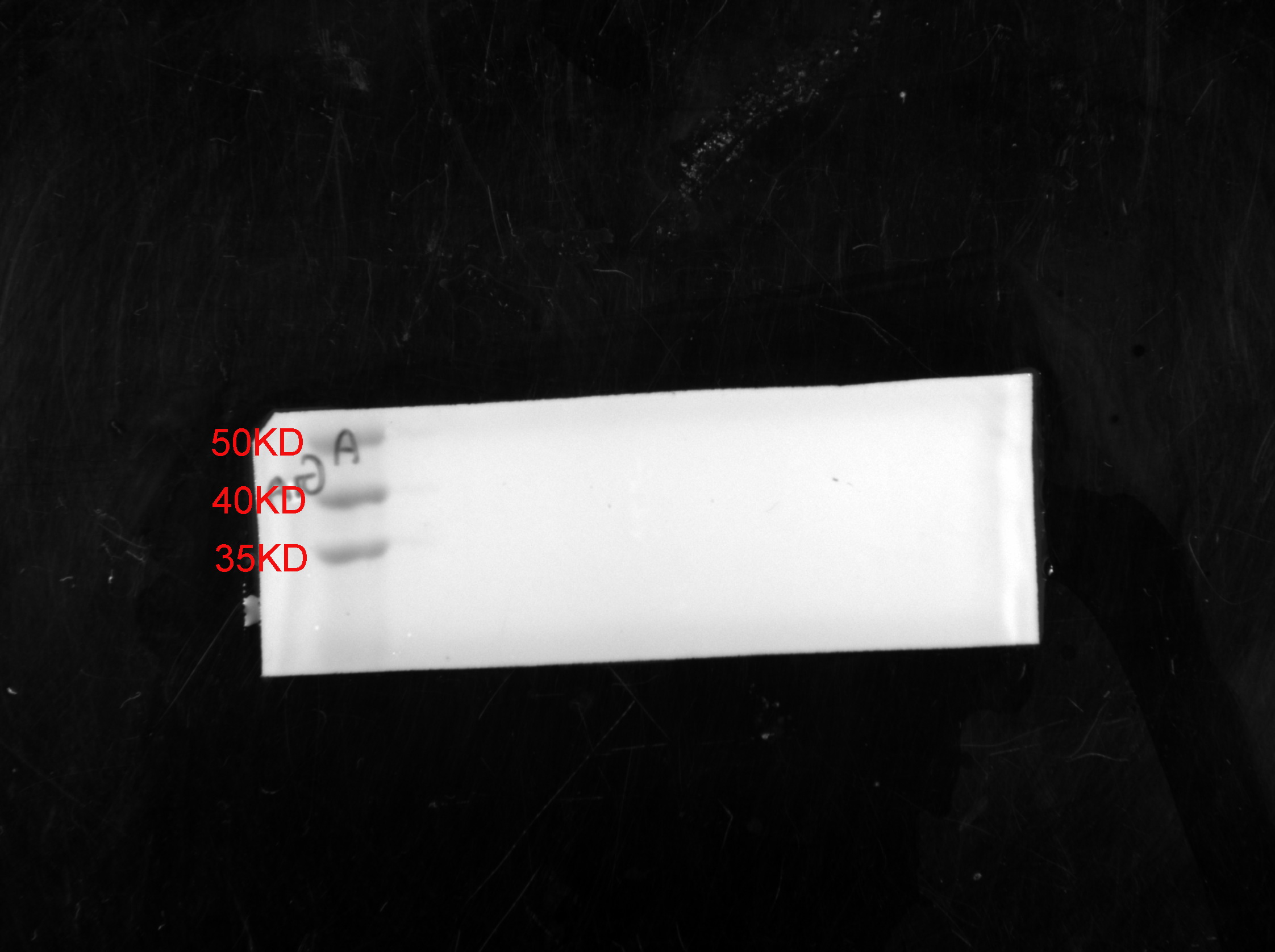

Supplement: Supplemental Information 1 — NMRAL2P overexpression plasmids (NMRAL2P-OE) or NMRAL2P knockdown (NMRAL2P-ASO) and their corresponding negative control groups (Vector or NC) were transferred into TU177 and AMC-HN-8 cells. After adding cycloheximide,proteins were collected at 0 h, 3 h, 6 h, 9 h and 12 h, respectively. Western blotting was used to detect the changes of ENO1 protein level to verify the effect of overexpression of NMRAL2P or knocking down NMRAL2P on ENO1 degradation. The protein blot images of ENO1. [file peerj-11-16140-s001.zip › ENO1 half-life/WB Verification of NMRAL2P knock-down half-life/AMC-HN-8 a┬-actin NMRAL2P-ASO White light.png]

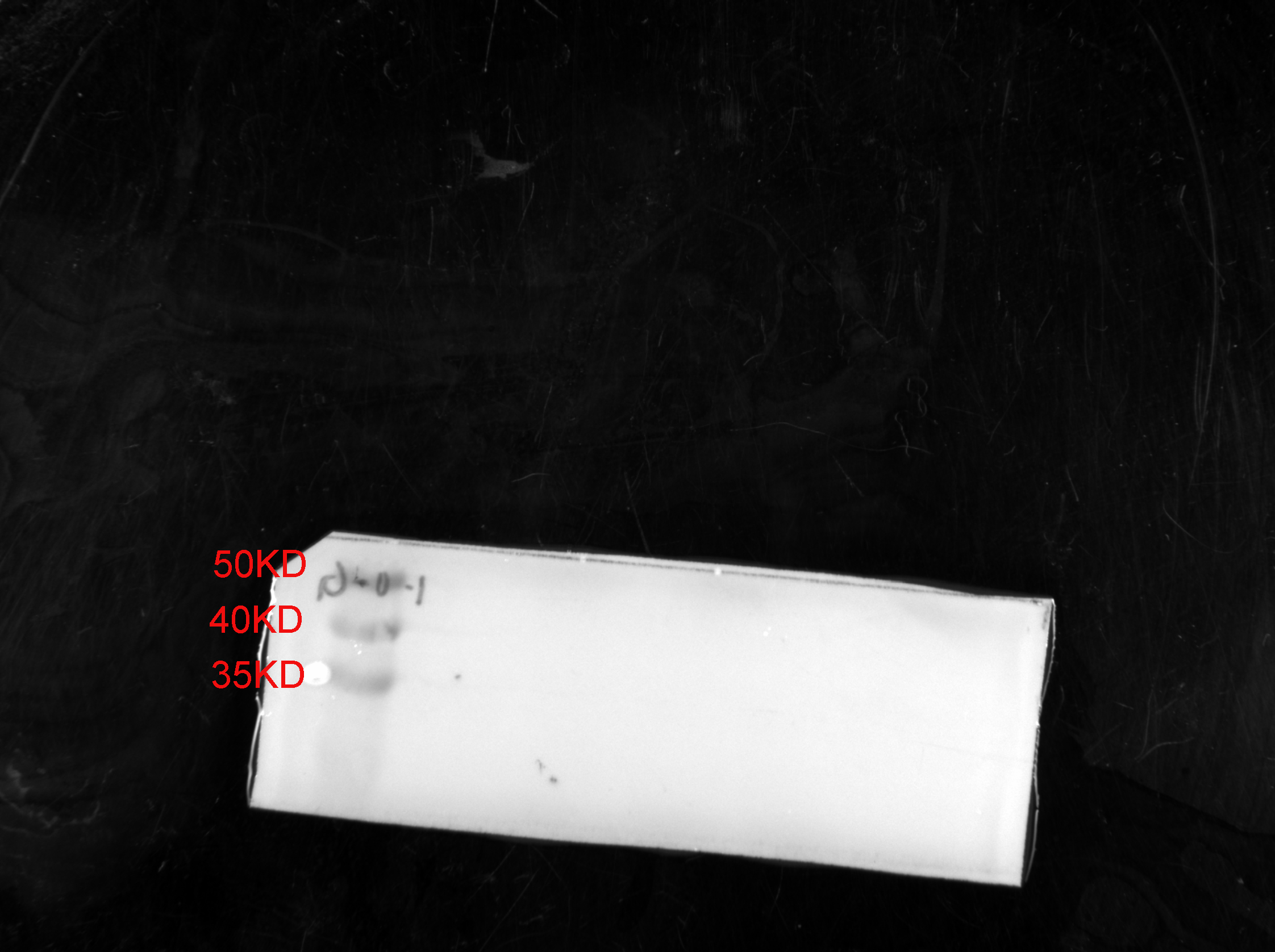

Supplement: Supplemental Information 1 — NMRAL2P overexpression plasmids (NMRAL2P-OE) or NMRAL2P knockdown (NMRAL2P-ASO) and their corresponding negative control groups (Vector or NC) were transferred into TU177 and AMC-HN-8 cells. After adding cycloheximide,proteins were collected at 0 h, 3 h, 6 h, 9 h and 12 h, respectively. Western blotting was used to detect the changes of ENO1 protein level to verify the effect of overexpression of NMRAL2P or knocking down NMRAL2P on ENO1 degradation. The protein blot images of ENO1. [file peerj-11-16140-s001.zip › ENO1 half-life/WB Verification of NMRAL2P knock-down half-life/TU177 a┬-actin NMRAL2P-ASO White light.png]

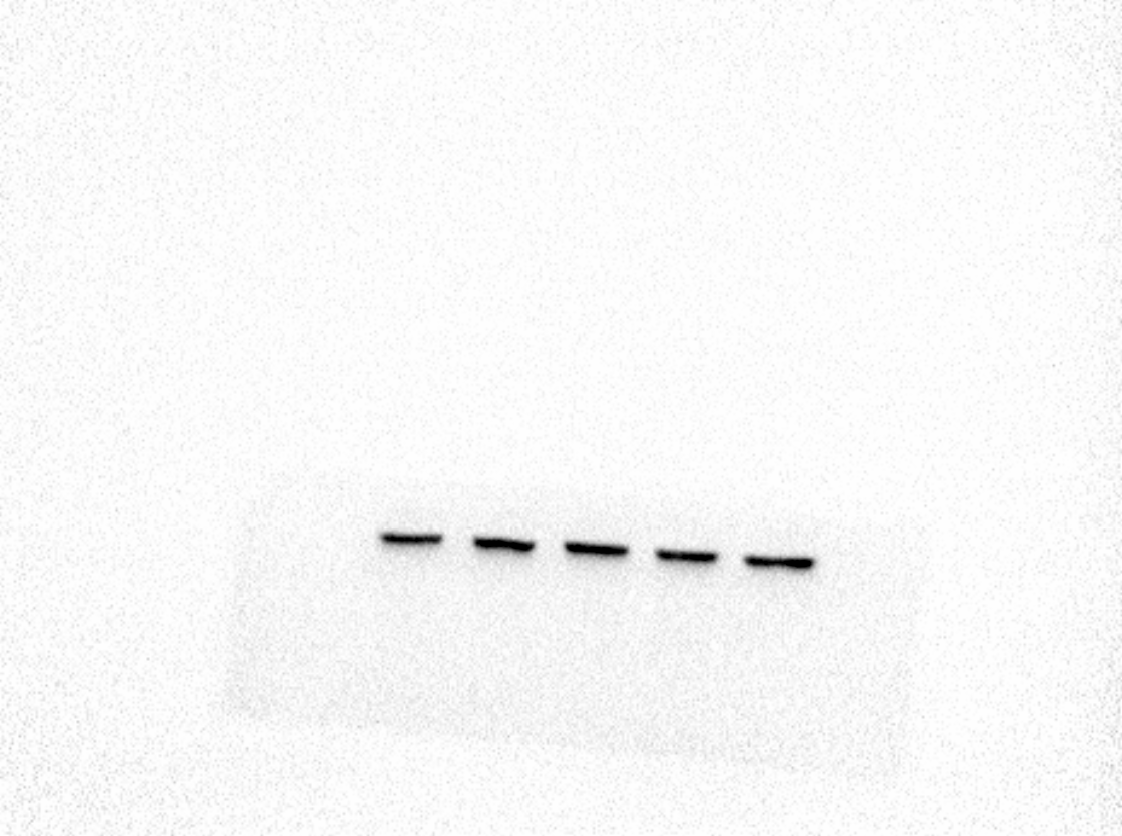

Supplement: Supplemental Information 1 — NMRAL2P overexpression plasmids (NMRAL2P-OE) or NMRAL2P knockdown (NMRAL2P-ASO) and their corresponding negative control groups (Vector or NC) were transferred into TU177 and AMC-HN-8 cells. After adding cycloheximide,proteins were collected at 0 h, 3 h, 6 h, 9 h and 12 h, respectively. Western blotting was used to detect the changes of ENO1 protein level to verify the effect of overexpression of NMRAL2P or knocking down NMRAL2P on ENO1 degradation. The protein blot images of ENO1. [file peerj-11-16140-s001.zip › ENO1 half-life/WB Verification of NMRAL2P knock-down half-life/TU177 a┬-actin NMRAL2P-ASO_Exposure_25.6sec.png]

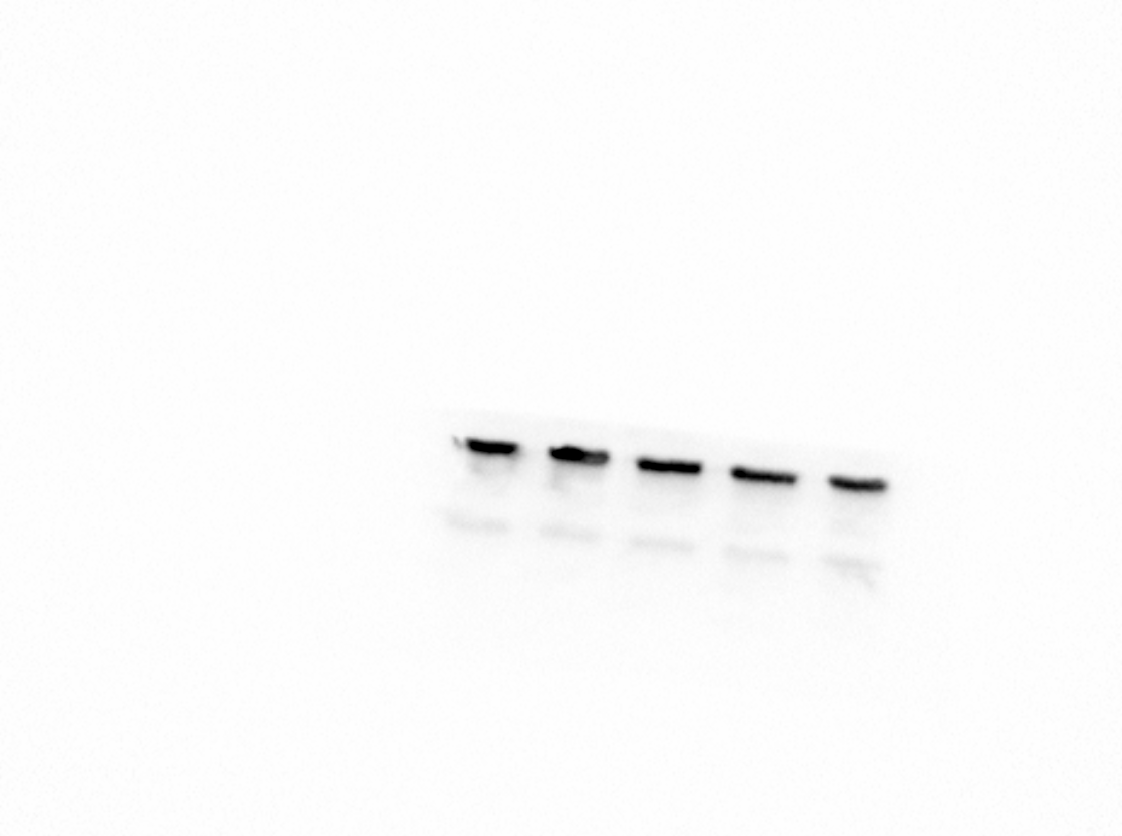

Supplement: Supplemental Information 1 — NMRAL2P overexpression plasmids (NMRAL2P-OE) or NMRAL2P knockdown (NMRAL2P-ASO) and their corresponding negative control groups (Vector or NC) were transferred into TU177 and AMC-HN-8 cells. After adding cycloheximide,proteins were collected at 0 h, 3 h, 6 h, 9 h and 12 h, respectively. Western blotting was used to detect the changes of ENO1 protein level to verify the effect of overexpression of NMRAL2P or knocking down NMRAL2P on ENO1 degradation. The protein blot images of ENO1. [file peerj-11-16140-s001.zip › ENO1 half-life/WB Verification of NMRAL2P knock-down half-life/TU177 ENO1 NC Exposure_1.0sec.png]

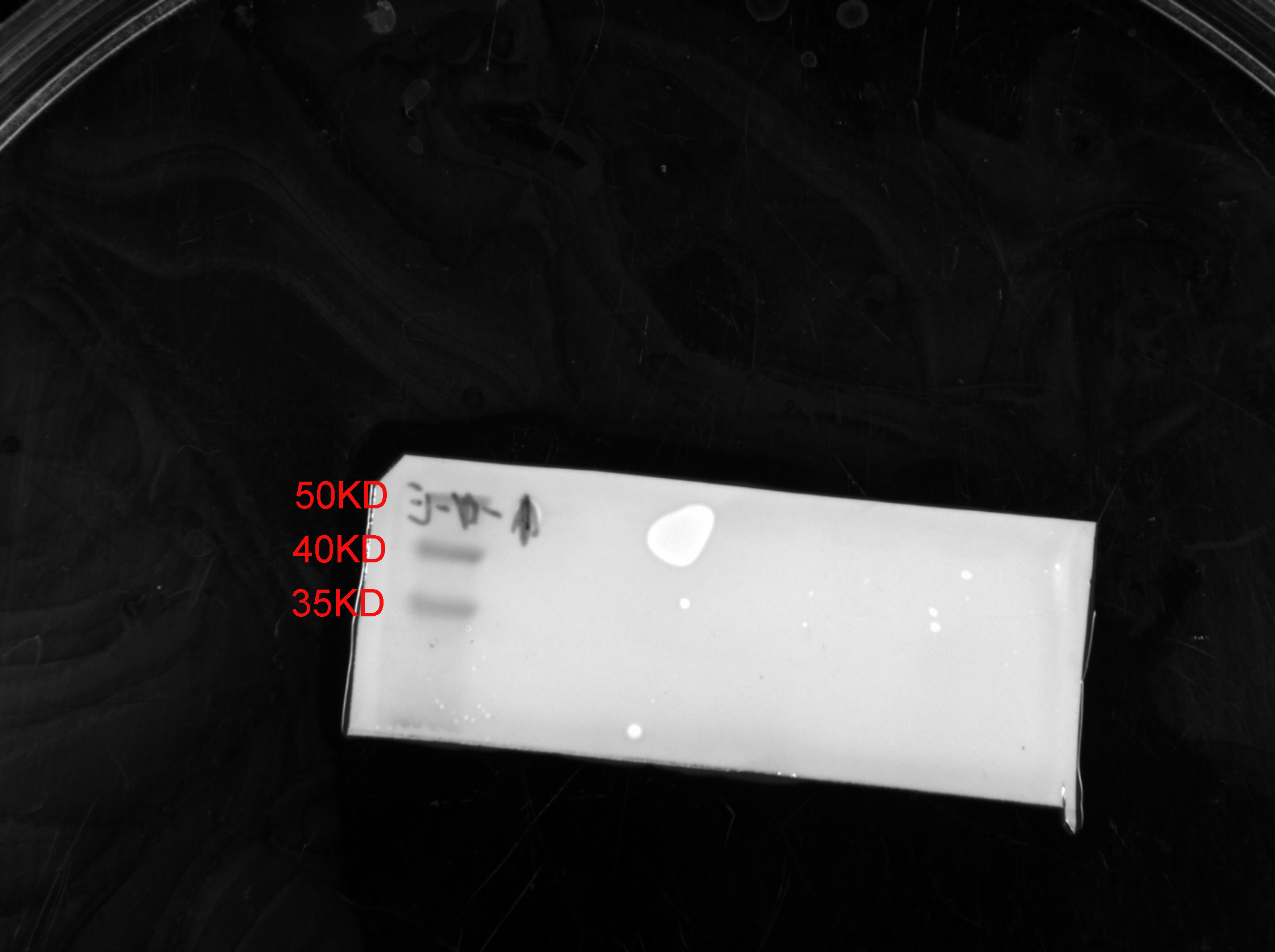

Supplement: Supplemental Information 1 — NMRAL2P overexpression plasmids (NMRAL2P-OE) or NMRAL2P knockdown (NMRAL2P-ASO) and their corresponding negative control groups (Vector or NC) were transferred into TU177 and AMC-HN-8 cells. After adding cycloheximide,proteins were collected at 0 h, 3 h, 6 h, 9 h and 12 h, respectively. Western blotting was used to detect the changes of ENO1 protein level to verify the effect of overexpression of NMRAL2P or knocking down NMRAL2P on ENO1 degradation. The protein blot images of ENO1. [file peerj-11-16140-s001.zip › ENO1 half-life/WB Verification of NMRAL2P knock-down half-life/TU177 ENO1 NC White light.png]

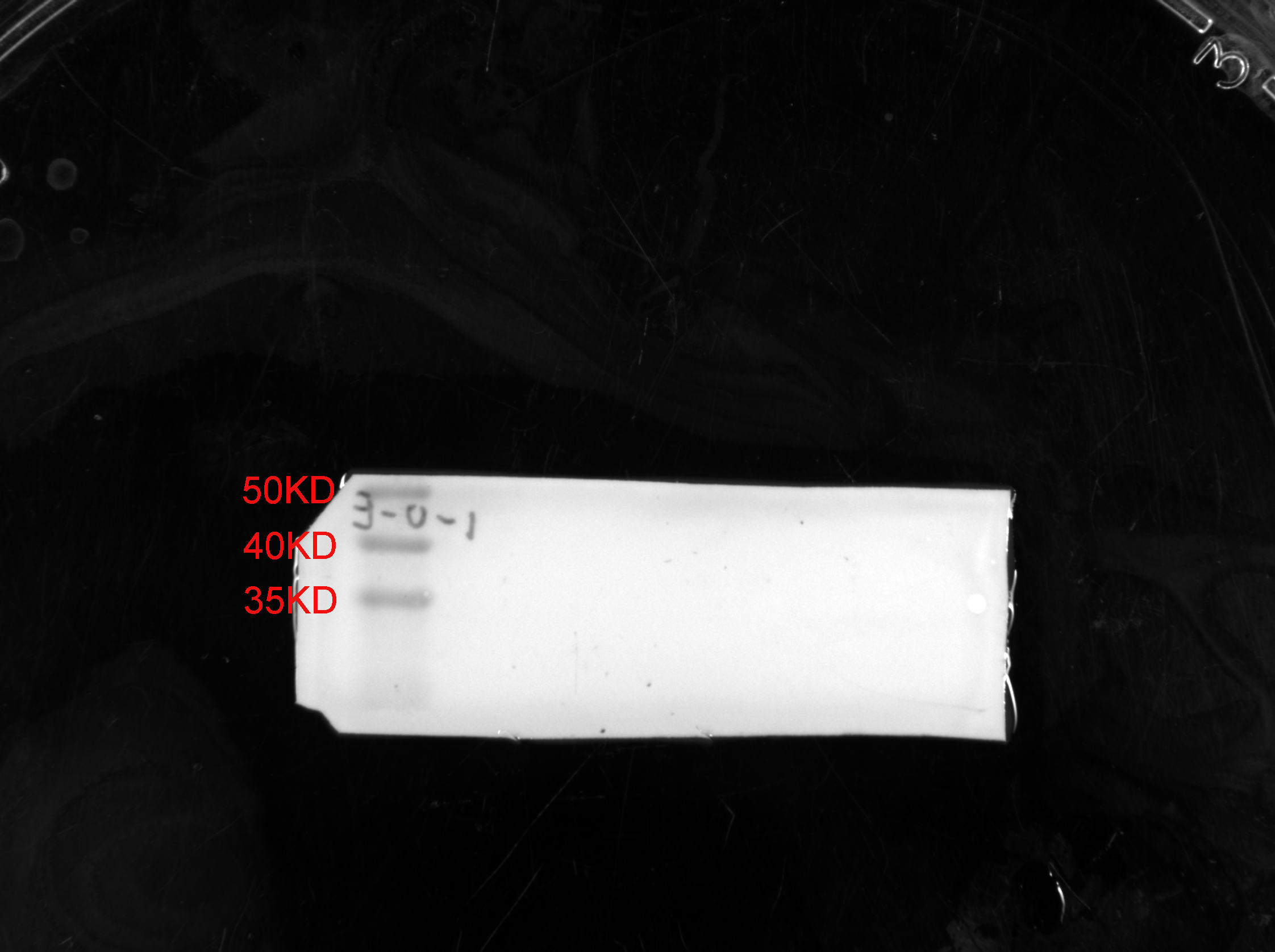

Supplement: Supplemental Information 1 — NMRAL2P overexpression plasmids (NMRAL2P-OE) or NMRAL2P knockdown (NMRAL2P-ASO) and their corresponding negative control groups (Vector or NC) were transferred into TU177 and AMC-HN-8 cells. After adding cycloheximide,proteins were collected at 0 h, 3 h, 6 h, 9 h and 12 h, respectively. Western blotting was used to detect the changes of ENO1 protein level to verify the effect of overexpression of NMRAL2P or knocking down NMRAL2P on ENO1 degradation. The protein blot images of ENO1. [file peerj-11-16140-s001.zip › ENO1 half-life/WB Verification of NMRAL2P knock-down half-life/TU177 ENO1 NMRAL2P-ASO White light.png]

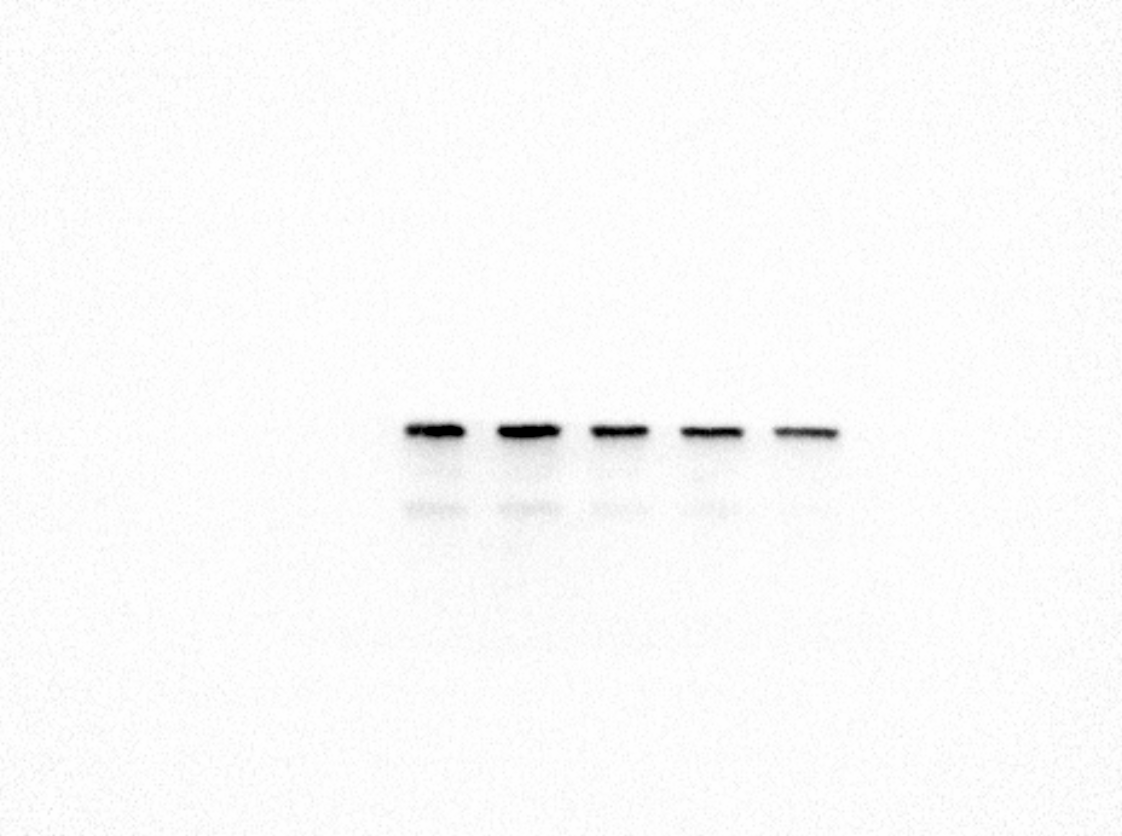

Supplement: Supplemental Information 1 — NMRAL2P overexpression plasmids (NMRAL2P-OE) or NMRAL2P knockdown (NMRAL2P-ASO) and their corresponding negative control groups (Vector or NC) were transferred into TU177 and AMC-HN-8 cells. After adding cycloheximide,proteins were collected at 0 h, 3 h, 6 h, 9 h and 12 h, respectively. Western blotting was used to detect the changes of ENO1 protein level to verify the effect of overexpression of NMRAL2P or knocking down NMRAL2P on ENO1 degradation. The protein blot images of ENO1. [file peerj-11-16140-s001.zip › ENO1 half-life/WB Verification of NMRAL2P knock-down half-life/TU177 ENO1 NMRAL2P-ASO_1.0sec.png]

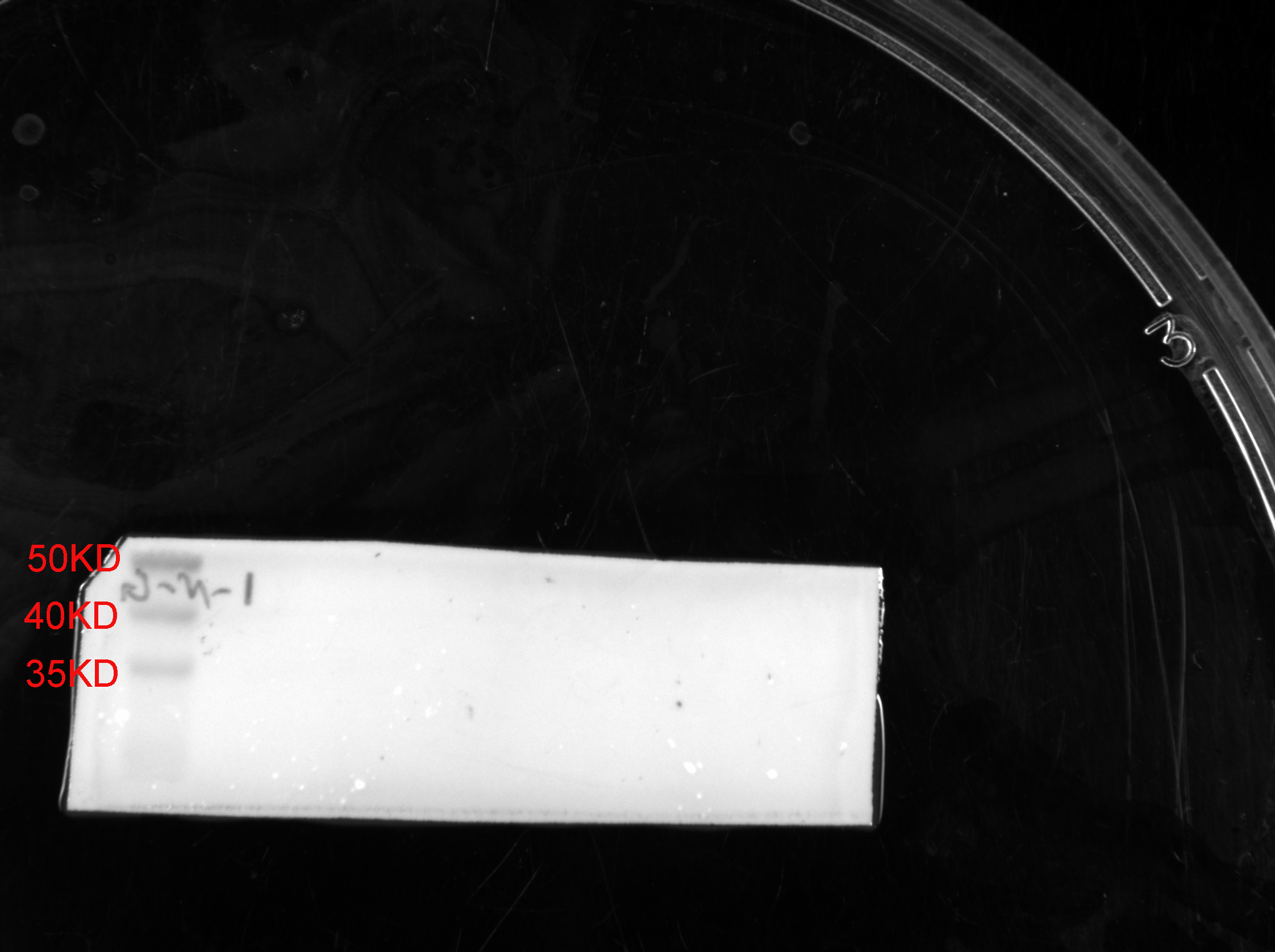

Supplement: Supplemental Information 1 — NMRAL2P overexpression plasmids (NMRAL2P-OE) or NMRAL2P knockdown (NMRAL2P-ASO) and their corresponding negative control groups (Vector or NC) were transferred into TU177 and AMC-HN-8 cells. After adding cycloheximide,proteins were collected at 0 h, 3 h, 6 h, 9 h and 12 h, respectively. Western blotting was used to detect the changes of ENO1 protein level to verify the effect of overexpression of NMRAL2P or knocking down NMRAL2P on ENO1 degradation. The protein blot images of ENO1. [file peerj-11-16140-s001.zip › ENO1 half-life/WB Verification of NMRAL2P knock-down half-life/TU177 a┬-actin NC White light.png]

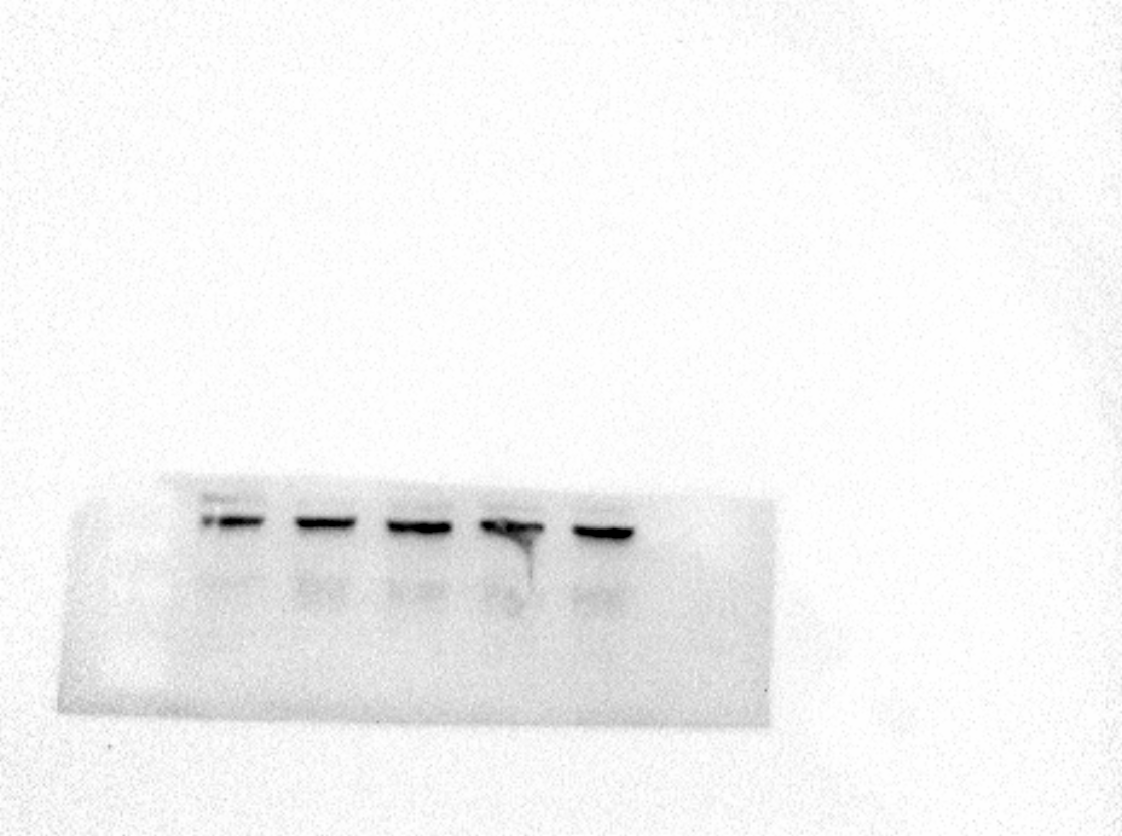

Supplement: Supplemental Information 1 — NMRAL2P overexpression plasmids (NMRAL2P-OE) or NMRAL2P knockdown (NMRAL2P-ASO) and their corresponding negative control groups (Vector or NC) were transferred into TU177 and AMC-HN-8 cells. After adding cycloheximide,proteins were collected at 0 h, 3 h, 6 h, 9 h and 12 h, respectively. Western blotting was used to detect the changes of ENO1 protein level to verify the effect of overexpression of NMRAL2P or knocking down NMRAL2P on ENO1 degradation. The protein blot images of ENO1. [file peerj-11-16140-s001.zip › ENO1 half-life/WB Verification of NMRAL2P knock-down half-life/TU177 a┬-actin NC_Exposure_50.1sec.png]

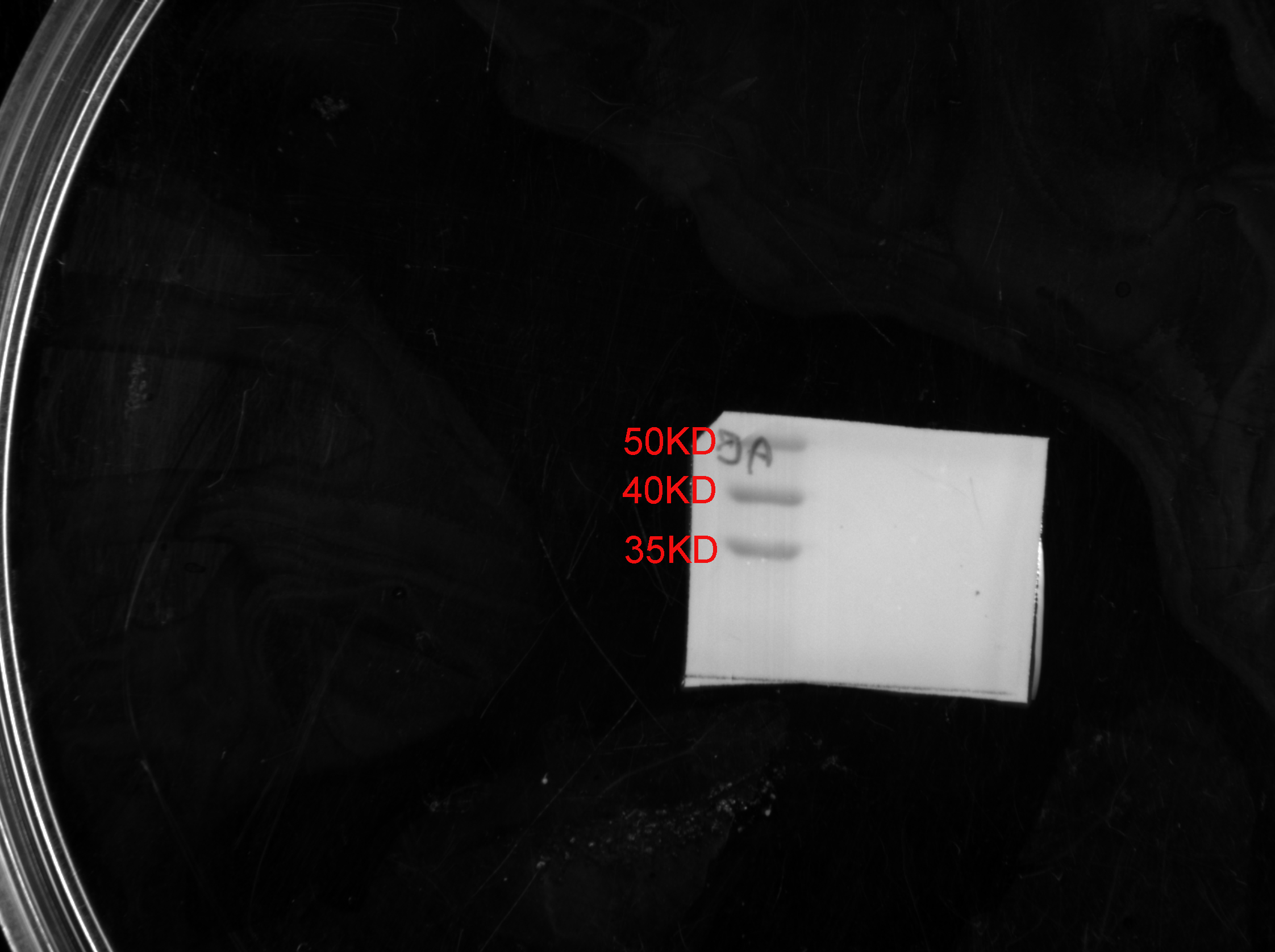

Supplement: Supplemental Information 2 — NMRAL2P overexpression plasmids (NMRAL2P-OE) or NMRAL2P knockdown (NMRAL2P-ASO) and their corresponding negative control groups (Vector or NC) were transferred into TU177 and AMC-HN-8 cells. 48 h later, the expression of ENO1 was detected. The image of protein ENO1 blot after NMRAL2P-oe or NMRAL2P-ASO were transferred into TU177 and AMC-HN-8 cells. [file peerj-11-16140-s002.zip › Overexpression and knock-down verification/WB Knock down NMRAL2P/AMC-HN-8 ENO1 White light.png]

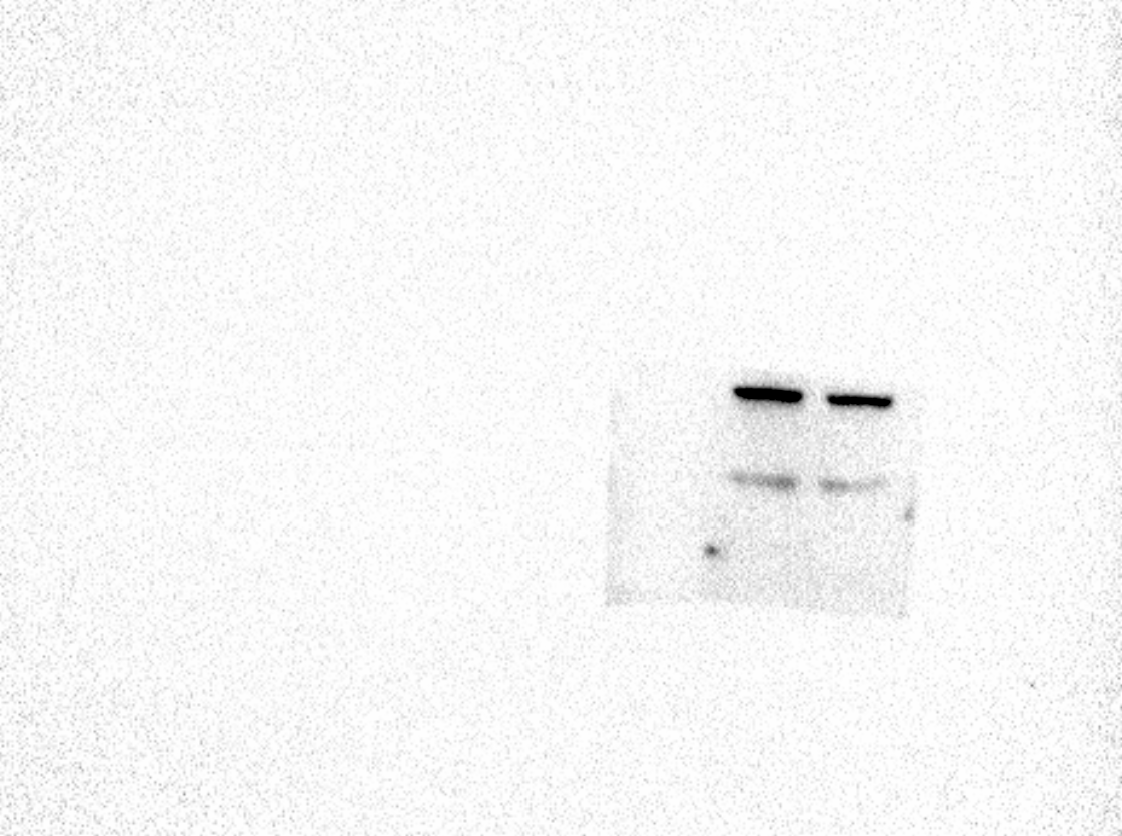

Supplement: Supplemental Information 2 — NMRAL2P overexpression plasmids (NMRAL2P-OE) or NMRAL2P knockdown (NMRAL2P-ASO) and their corresponding negative control groups (Vector or NC) were transferred into TU177 and AMC-HN-8 cells. 48 h later, the expression of ENO1 was detected. The image of protein ENO1 blot after NMRAL2P-oe or NMRAL2P-ASO were transferred into TU177 and AMC-HN-8 cells. [file peerj-11-16140-s002.zip › Overexpression and knock-down verification/WB Knock down NMRAL2P/AMC-HN-8 ENO1_Exposure_19.5sec.png]

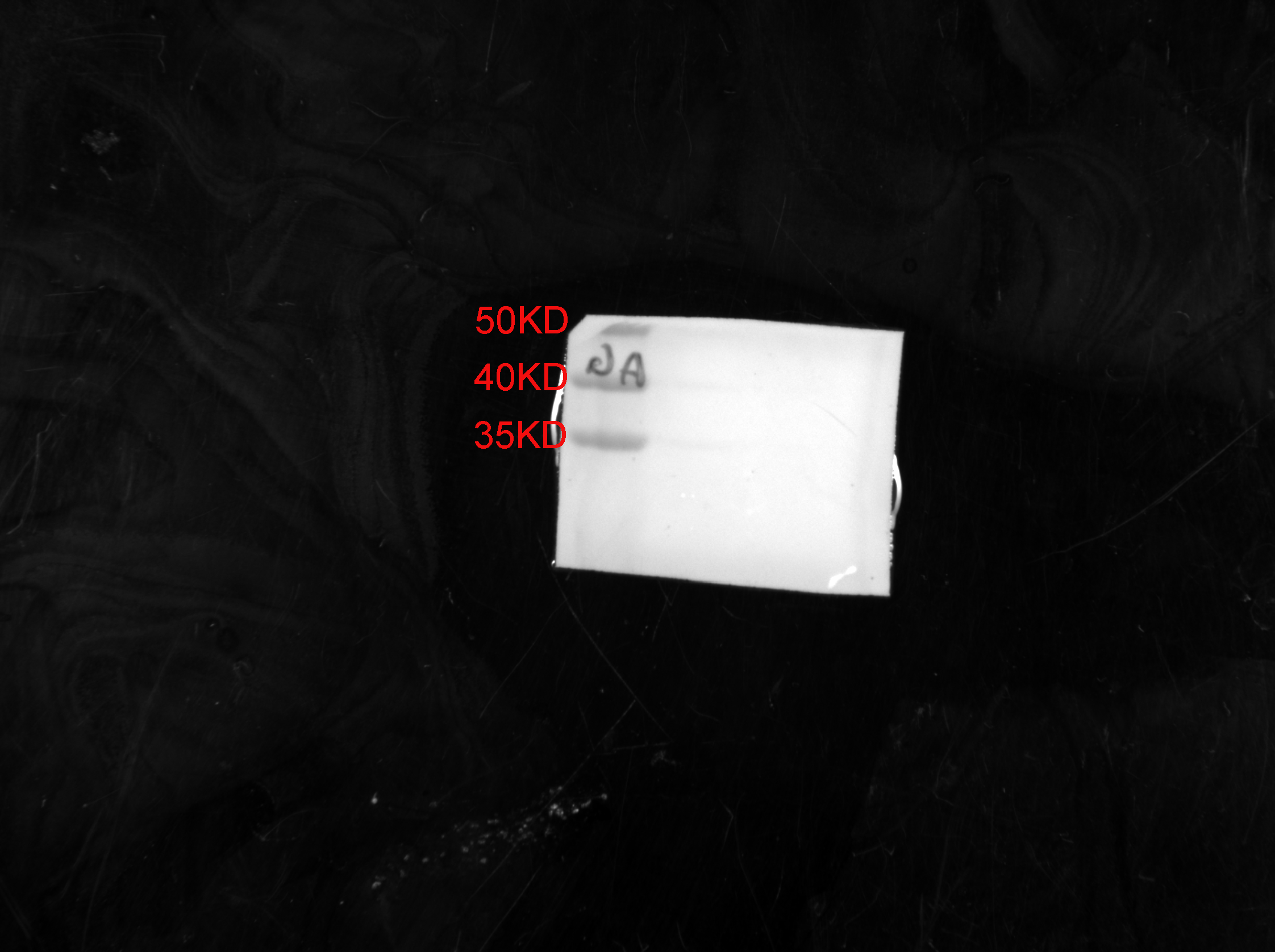

Supplement: Supplemental Information 2 — NMRAL2P overexpression plasmids (NMRAL2P-OE) or NMRAL2P knockdown (NMRAL2P-ASO) and their corresponding negative control groups (Vector or NC) were transferred into TU177 and AMC-HN-8 cells. 48 h later, the expression of ENO1 was detected. The image of protein ENO1 blot after NMRAL2P-oe or NMRAL2P-ASO were transferred into TU177 and AMC-HN-8 cells. [file peerj-11-16140-s002.zip › Overexpression and knock-down verification/WB Knock down NMRAL2P/AMC-HN-8 a┬-actin White light.png]

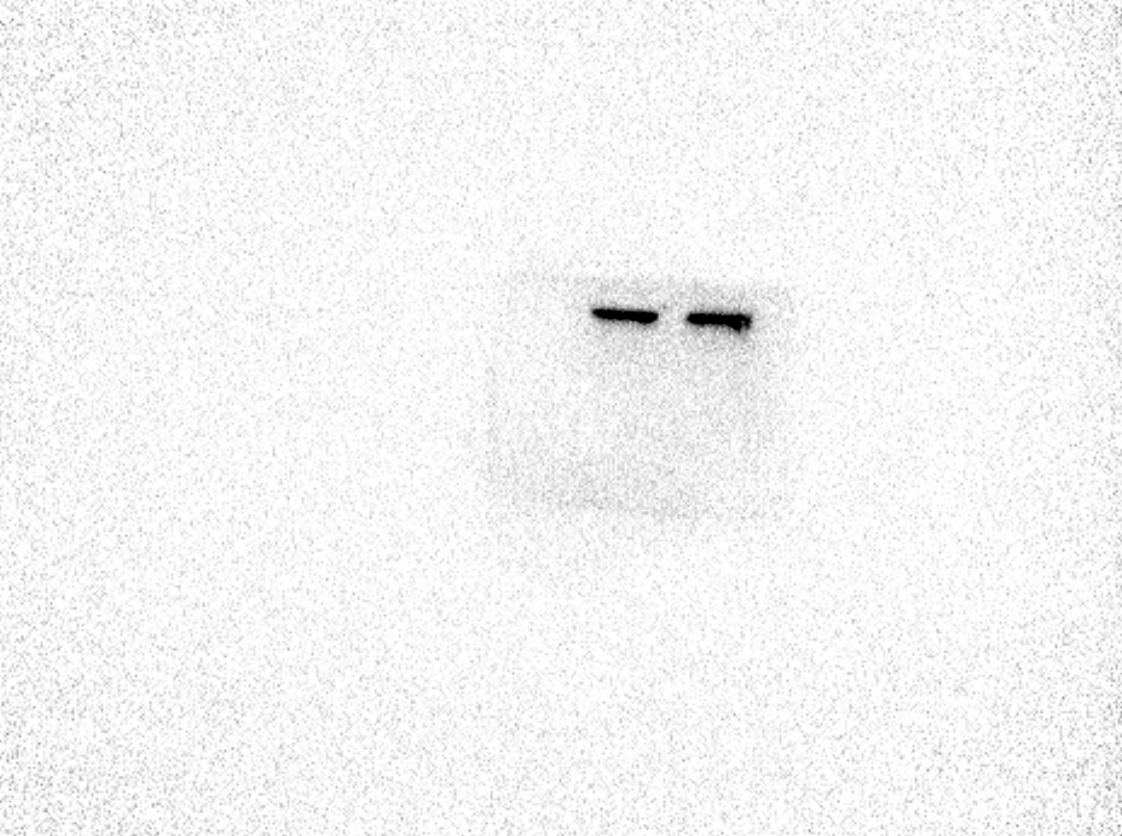

Supplement: Supplemental Information 2 — NMRAL2P overexpression plasmids (NMRAL2P-OE) or NMRAL2P knockdown (NMRAL2P-ASO) and their corresponding negative control groups (Vector or NC) were transferred into TU177 and AMC-HN-8 cells. 48 h later, the expression of ENO1 was detected. The image of protein ENO1 blot after NMRAL2P-oe or NMRAL2P-ASO were transferred into TU177 and AMC-HN-8 cells. [file peerj-11-16140-s002.zip › Overexpression and knock-down verification/WB Knock down NMRAL2P/AMC-HN-8 a┬-actin_Exposure_5.2sec.png]

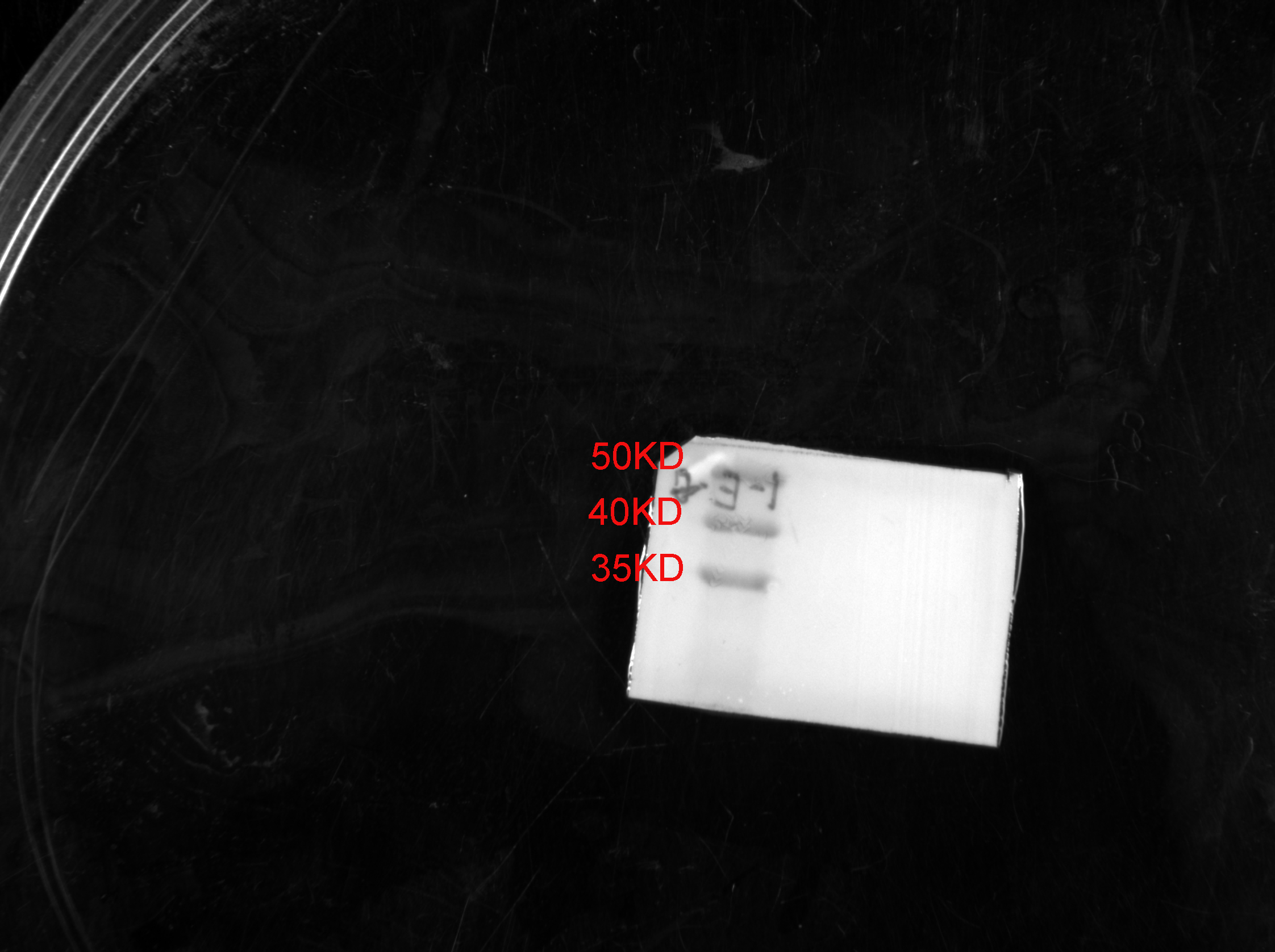

Supplement: Supplemental Information 2 — NMRAL2P overexpression plasmids (NMRAL2P-OE) or NMRAL2P knockdown (NMRAL2P-ASO) and their corresponding negative control groups (Vector or NC) were transferred into TU177 and AMC-HN-8 cells. 48 h later, the expression of ENO1 was detected. The image of protein ENO1 blot after NMRAL2P-oe or NMRAL2P-ASO were transferred into TU177 and AMC-HN-8 cells. [file peerj-11-16140-s002.zip › Overexpression and knock-down verification/WB Knock down NMRAL2P/TU177 ENO1 White light.png]

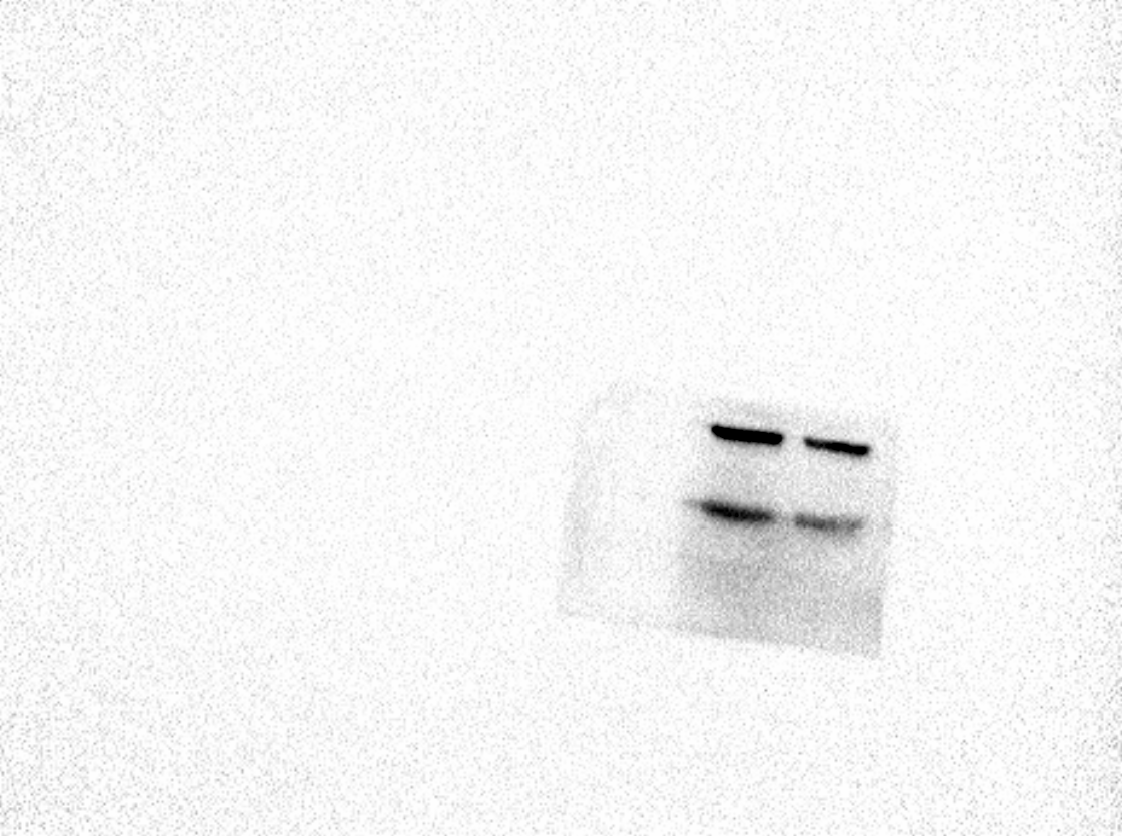

Supplement: Supplemental Information 2 — NMRAL2P overexpression plasmids (NMRAL2P-OE) or NMRAL2P knockdown (NMRAL2P-ASO) and their corresponding negative control groups (Vector or NC) were transferred into TU177 and AMC-HN-8 cells. 48 h later, the expression of ENO1 was detected. The image of protein ENO1 blot after NMRAL2P-oe or NMRAL2P-ASO were transferred into TU177 and AMC-HN-8 cells. [file peerj-11-16140-s002.zip › Overexpression and knock-down verification/WB Knock down NMRAL2P/TU177 ENO1_Exposure_13.2sec.png]

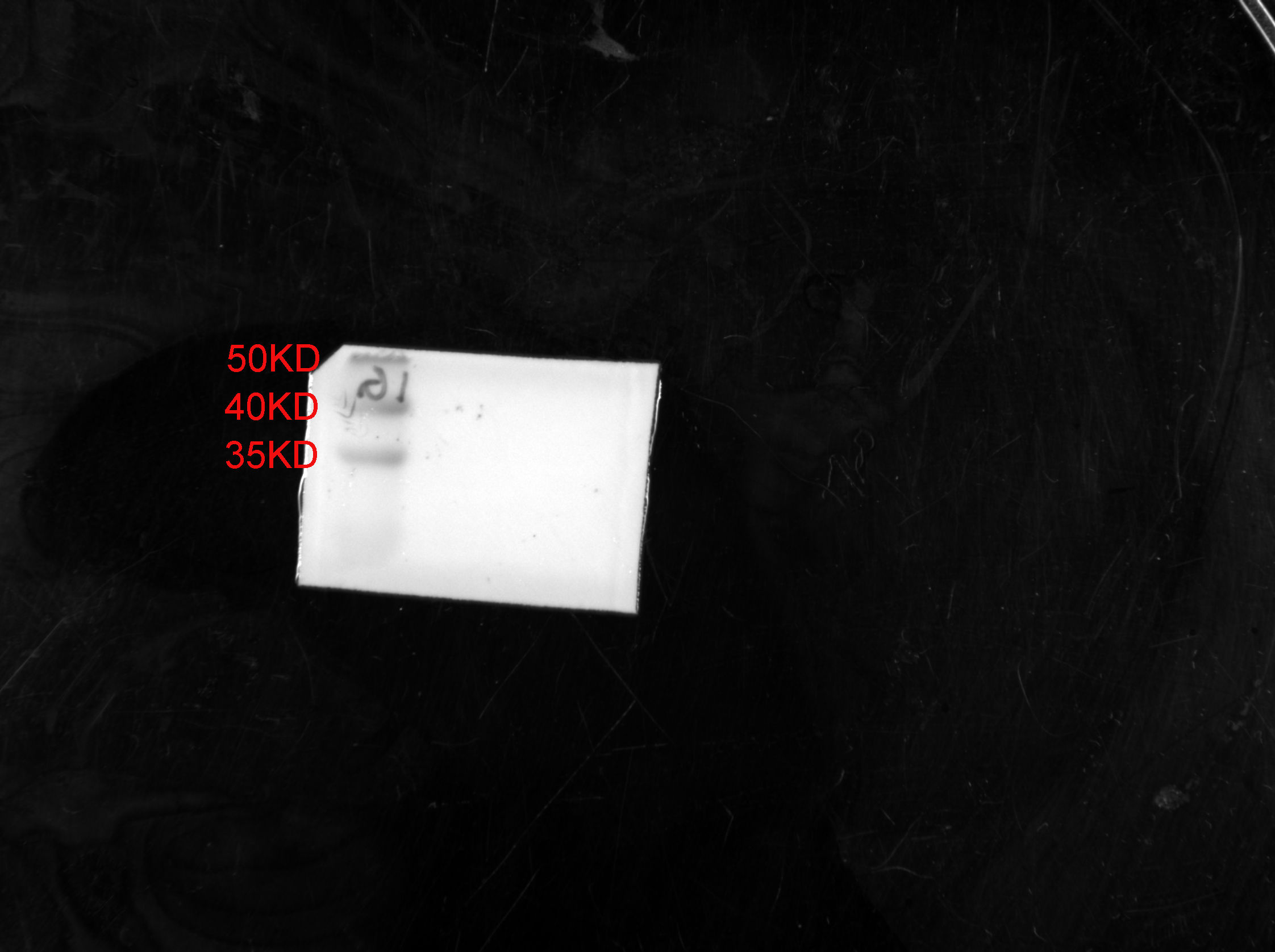

Supplement: Supplemental Information 2 — NMRAL2P overexpression plasmids (NMRAL2P-OE) or NMRAL2P knockdown (NMRAL2P-ASO) and their corresponding negative control groups (Vector or NC) were transferred into TU177 and AMC-HN-8 cells. 48 h later, the expression of ENO1 was detected. The image of protein ENO1 blot after NMRAL2P-oe or NMRAL2P-ASO were transferred into TU177 and AMC-HN-8 cells. [file peerj-11-16140-s002.zip › Overexpression and knock-down verification/WB Knock down NMRAL2P/TU177 a┬-actin White light_.png]

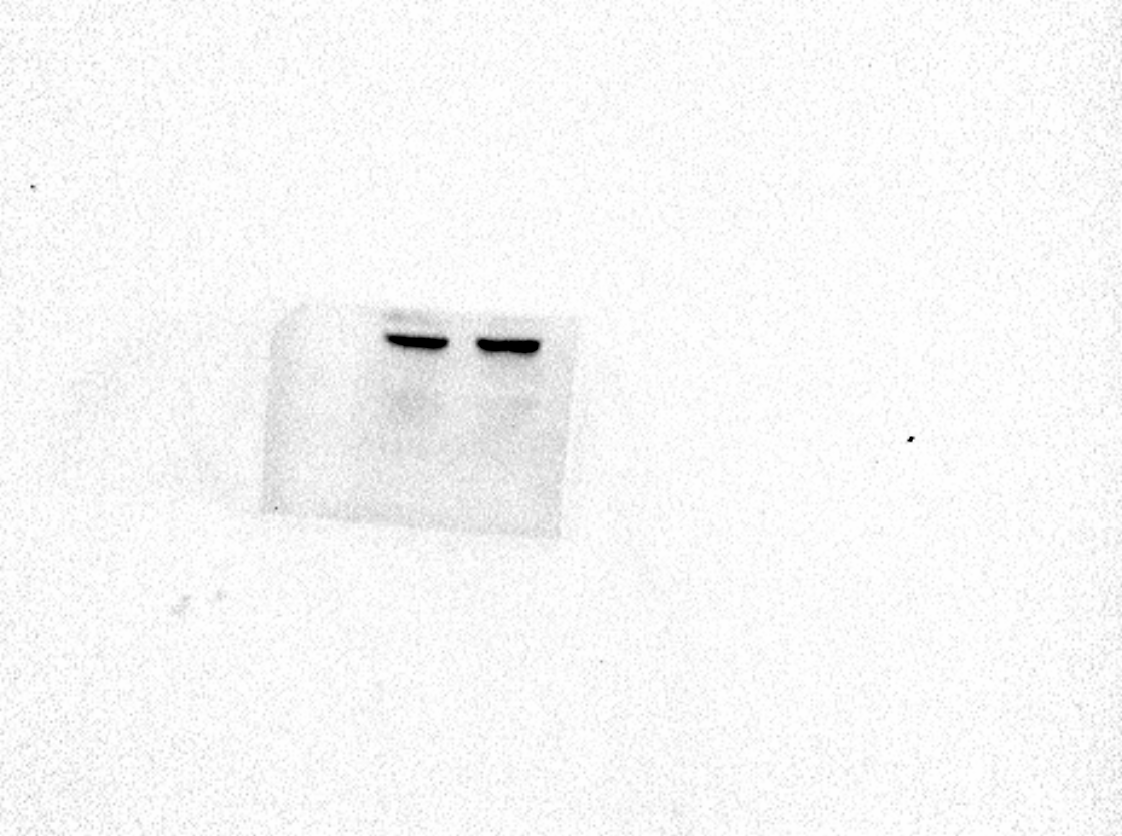

Supplement: Supplemental Information 2 — NMRAL2P overexpression plasmids (NMRAL2P-OE) or NMRAL2P knockdown (NMRAL2P-ASO) and their corresponding negative control groups (Vector or NC) were transferred into TU177 and AMC-HN-8 cells. 48 h later, the expression of ENO1 was detected. The image of protein ENO1 blot after NMRAL2P-oe or NMRAL2P-ASO were transferred into TU177 and AMC-HN-8 cells. [file peerj-11-16140-s002.zip › Overexpression and knock-down verification/WB Knock down NMRAL2P/TU177 a┬-actin_Exposure_69.5sec.png]

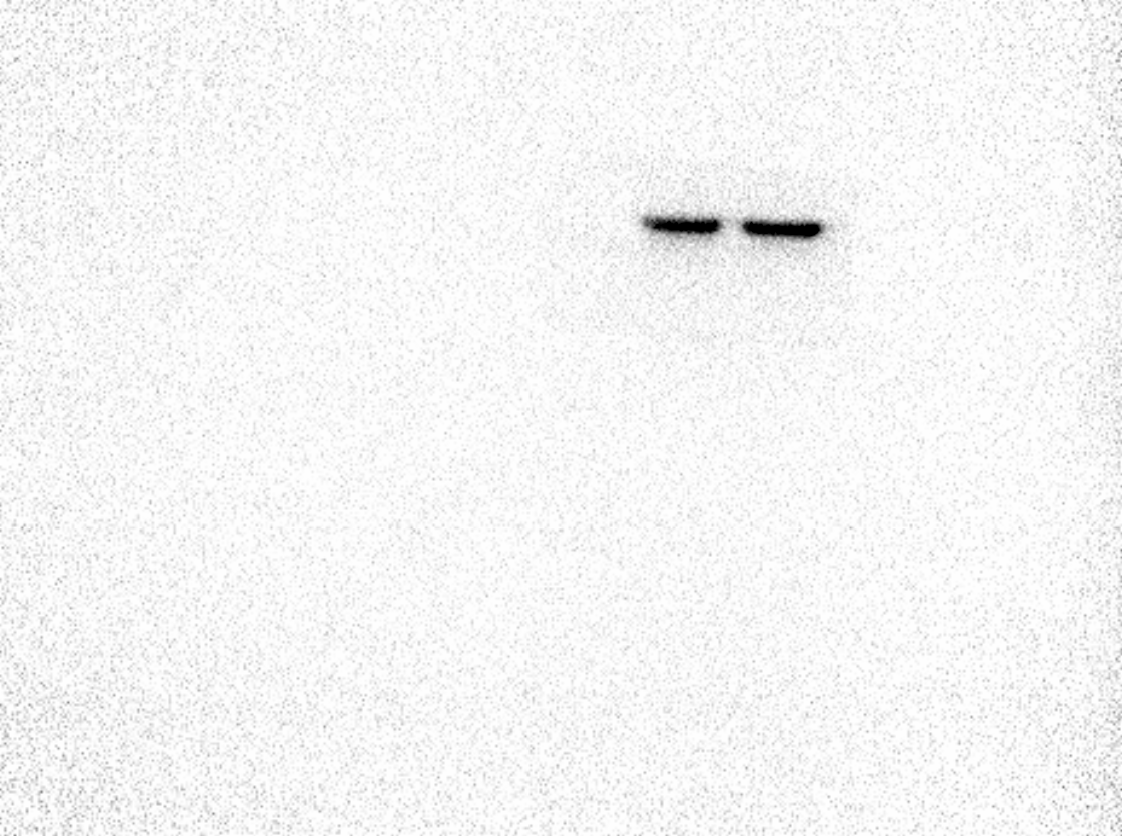

Supplement: Supplemental Information 2 — NMRAL2P overexpression plasmids (NMRAL2P-OE) or NMRAL2P knockdown (NMRAL2P-ASO) and their corresponding negative control groups (Vector or NC) were transferred into TU177 and AMC-HN-8 cells. 48 h later, the expression of ENO1 was detected. The image of protein ENO1 blot after NMRAL2P-oe or NMRAL2P-ASO were transferred into TU177 and AMC-HN-8 cells. [file peerj-11-16140-s002.zip › Overexpression and knock-down verification/WB Overexpression NMRAL2P/AMC -HN-8 a┬-actin_Exposure_9.0sec.png]

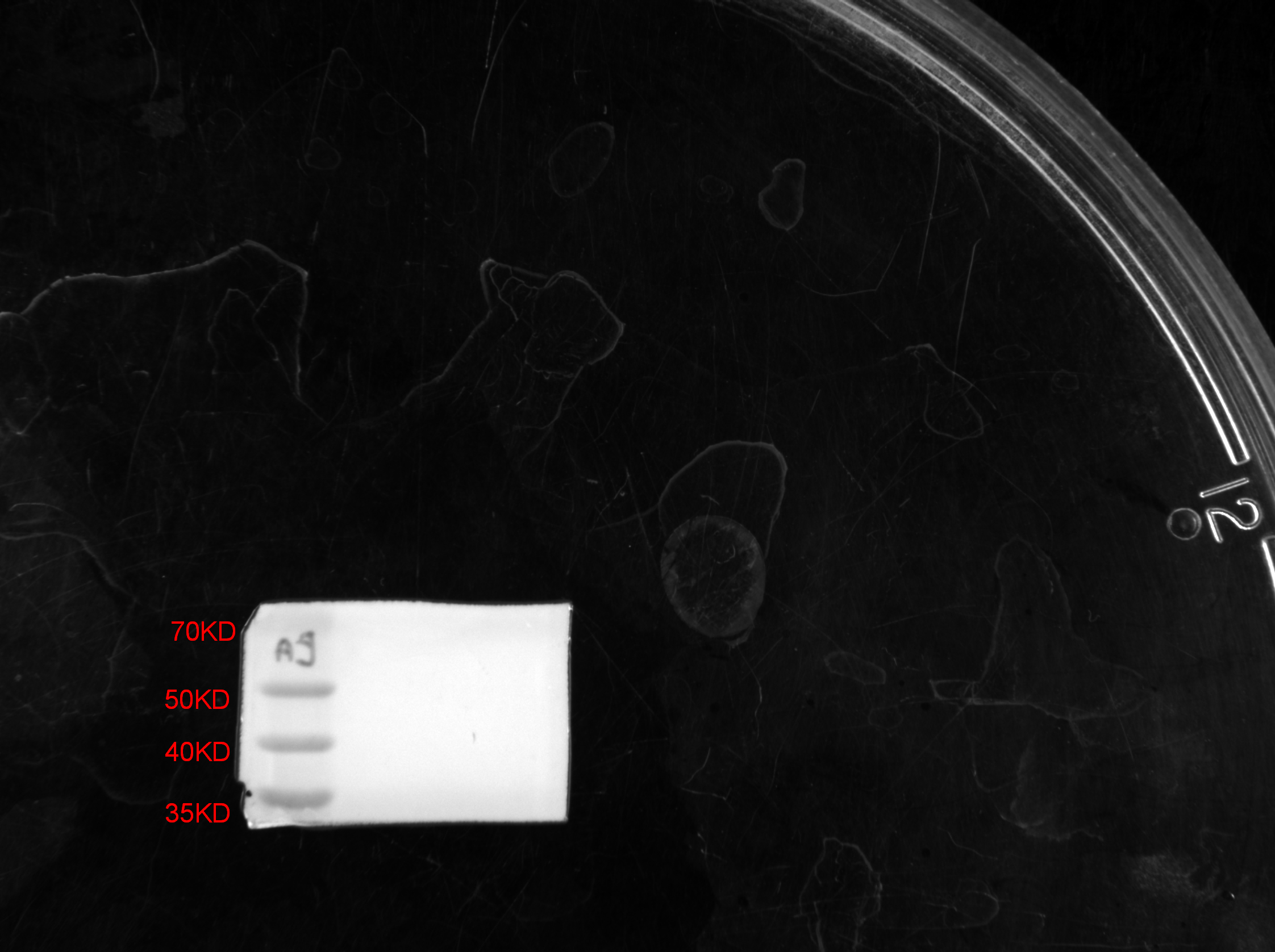

Supplement: Supplemental Information 2 — NMRAL2P overexpression plasmids (NMRAL2P-OE) or NMRAL2P knockdown (NMRAL2P-ASO) and their corresponding negative control groups (Vector or NC) were transferred into TU177 and AMC-HN-8 cells. 48 h later, the expression of ENO1 was detected. The image of protein ENO1 blot after NMRAL2P-oe or NMRAL2P-ASO were transferred into TU177 and AMC-HN-8 cells. [file peerj-11-16140-s002.zip › Overexpression and knock-down verification/WB Overexpression NMRAL2P/AMC-HN-8 ENO1 White light.png]

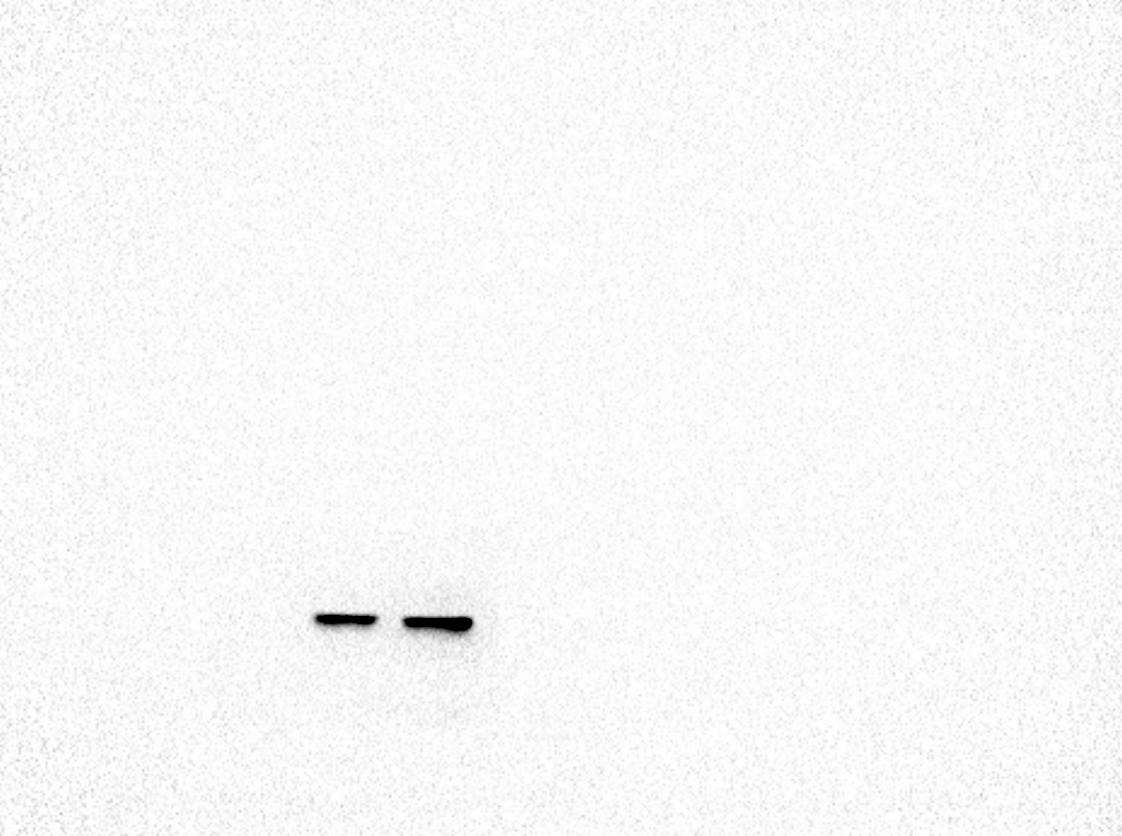

Supplement: Supplemental Information 2 — NMRAL2P overexpression plasmids (NMRAL2P-OE) or NMRAL2P knockdown (NMRAL2P-ASO) and their corresponding negative control groups (Vector or NC) were transferred into TU177 and AMC-HN-8 cells. 48 h later, the expression of ENO1 was detected. The image of protein ENO1 blot after NMRAL2P-oe or NMRAL2P-ASO were transferred into TU177 and AMC-HN-8 cells. [file peerj-11-16140-s002.zip › Overexpression and knock-down verification/WB Overexpression NMRAL2P/AMC-HN-8 ENO1_Exposure_1.0sec.png]

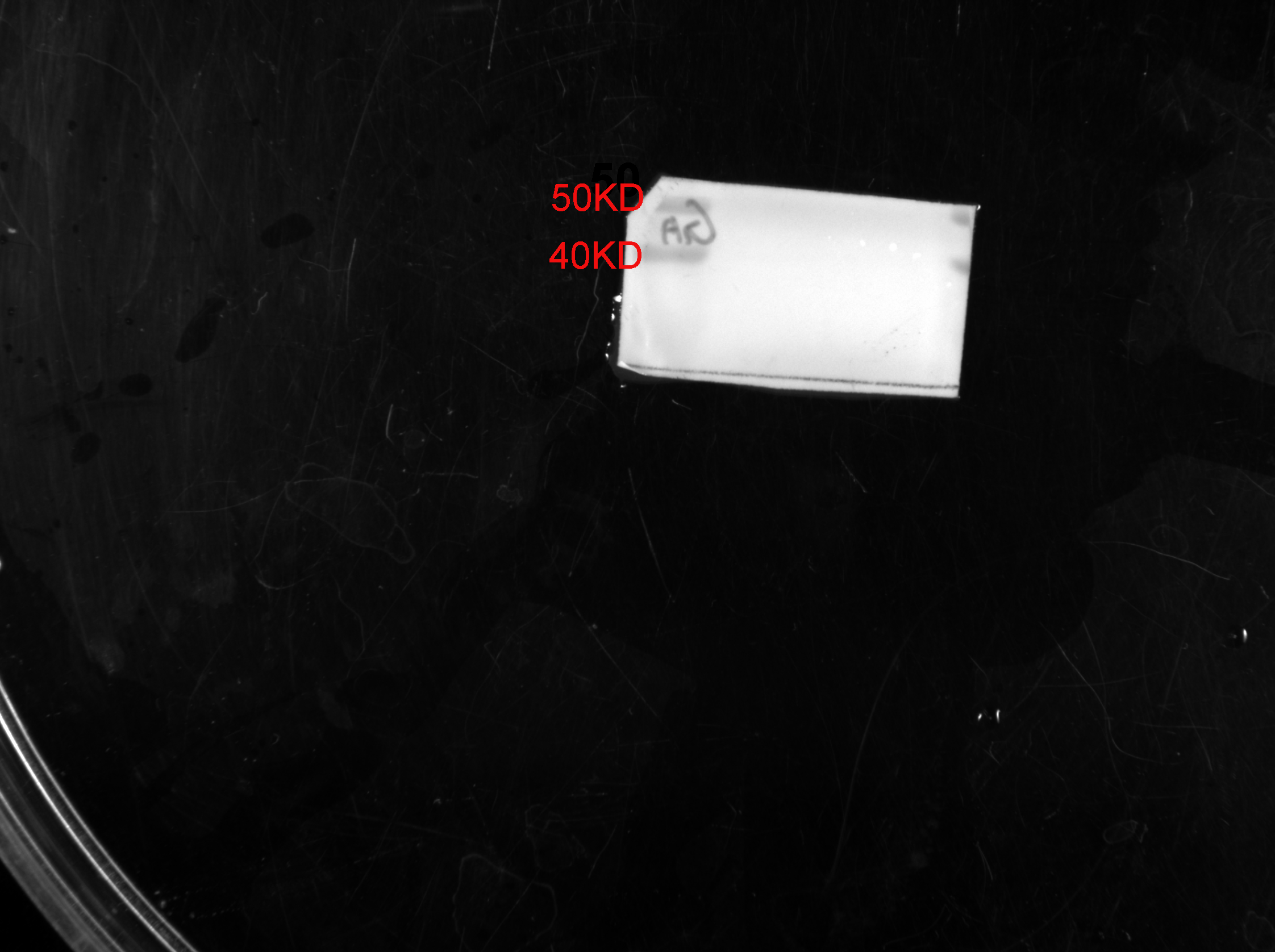

Supplement: Supplemental Information 2 — NMRAL2P overexpression plasmids (NMRAL2P-OE) or NMRAL2P knockdown (NMRAL2P-ASO) and their corresponding negative control groups (Vector or NC) were transferred into TU177 and AMC-HN-8 cells. 48 h later, the expression of ENO1 was detected. The image of protein ENO1 blot after NMRAL2P-oe or NMRAL2P-ASO were transferred into TU177 and AMC-HN-8 cells. [file peerj-11-16140-s002.zip › Overexpression and knock-down verification/WB Overexpression NMRAL2P/AMC-HN-8 a┬-actin White light_.png]

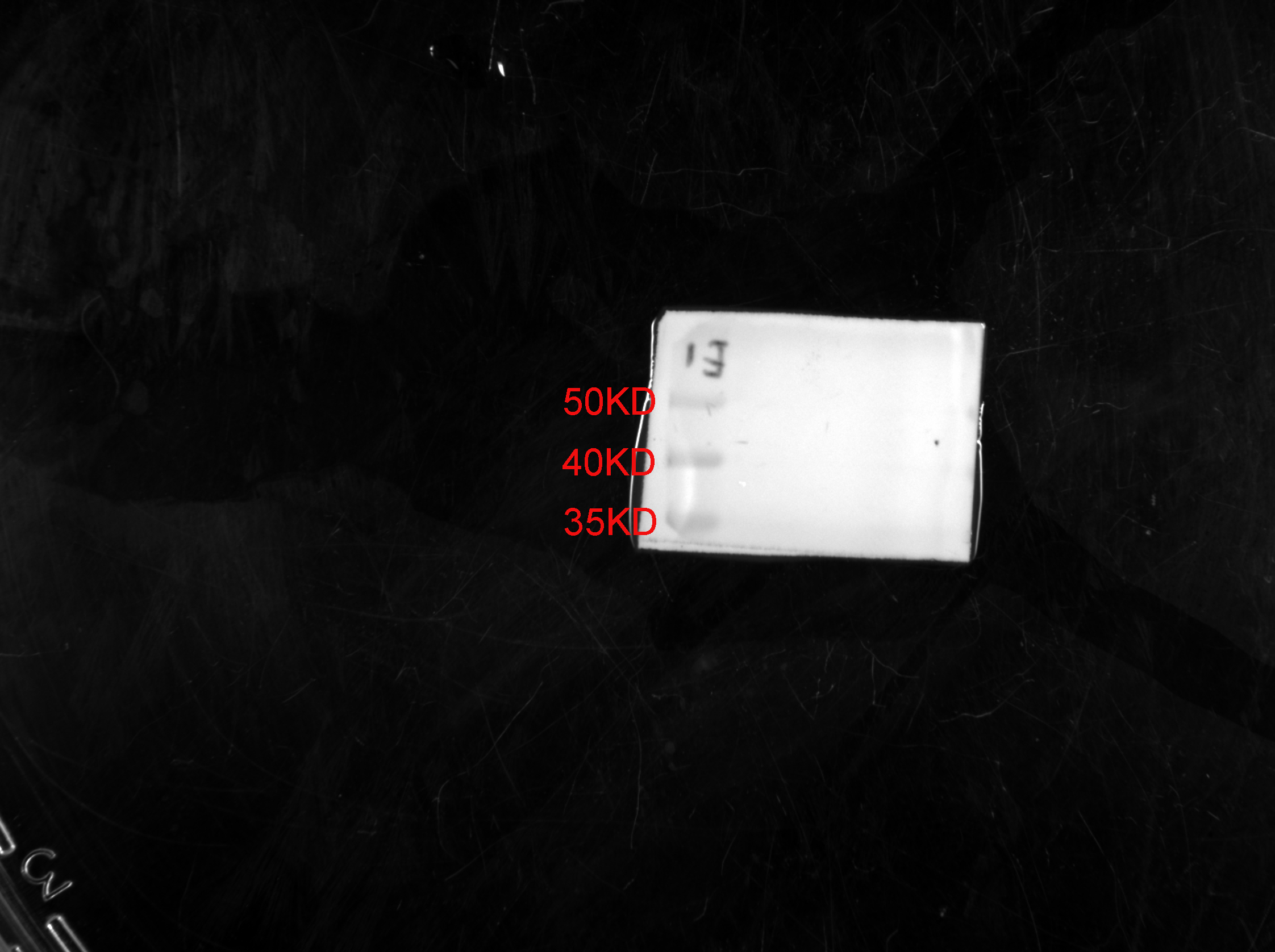

Supplement: Supplemental Information 2 — NMRAL2P overexpression plasmids (NMRAL2P-OE) or NMRAL2P knockdown (NMRAL2P-ASO) and their corresponding negative control groups (Vector or NC) were transferred into TU177 and AMC-HN-8 cells. 48 h later, the expression of ENO1 was detected. The image of protein ENO1 blot after NMRAL2P-oe or NMRAL2P-ASO were transferred into TU177 and AMC-HN-8 cells. [file peerj-11-16140-s002.zip › Overexpression and knock-down verification/WB Overexpression NMRAL2P/TU177 ENO1 White light_.png]

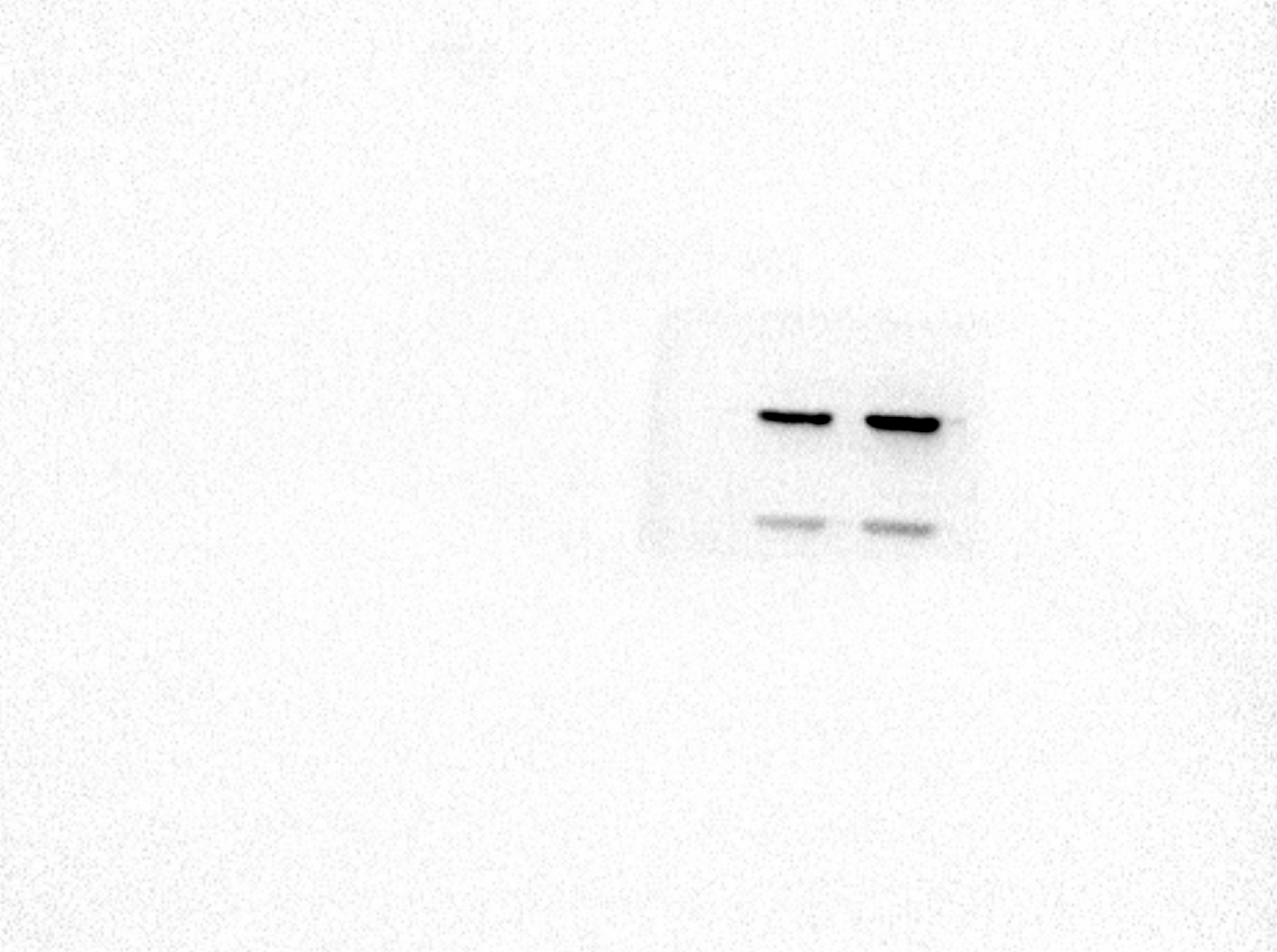

Supplement: Supplemental Information 2 — NMRAL2P overexpression plasmids (NMRAL2P-OE) or NMRAL2P knockdown (NMRAL2P-ASO) and their corresponding negative control groups (Vector or NC) were transferred into TU177 and AMC-HN-8 cells. 48 h later, the expression of ENO1 was detected. The image of protein ENO1 blot after NMRAL2P-oe or NMRAL2P-ASO were transferred into TU177 and AMC-HN-8 cells. [file peerj-11-16140-s002.zip › Overexpression and knock-down verification/WB Overexpression NMRAL2P/TU177 ENO1_Exposure_7.0sec.png]

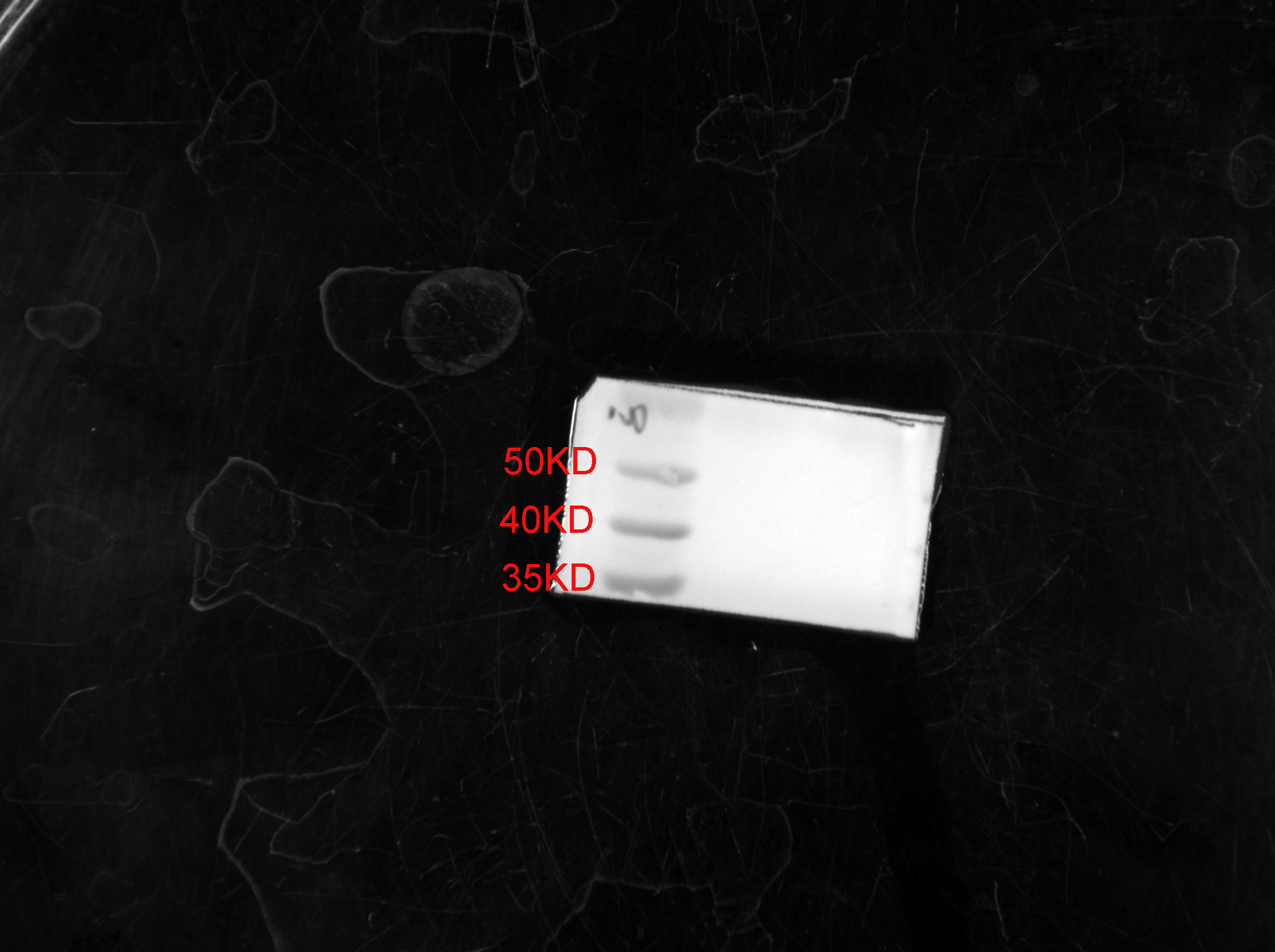

Supplement: Supplemental Information 2 — NMRAL2P overexpression plasmids (NMRAL2P-OE) or NMRAL2P knockdown (NMRAL2P-ASO) and their corresponding negative control groups (Vector or NC) were transferred into TU177 and AMC-HN-8 cells. 48 h later, the expression of ENO1 was detected. The image of protein ENO1 blot after NMRAL2P-oe or NMRAL2P-ASO were transferred into TU177 and AMC-HN-8 cells. [file peerj-11-16140-s002.zip › Overexpression and knock-down verification/WB Overexpression NMRAL2P/TU177 a┬-actin White light_.png]

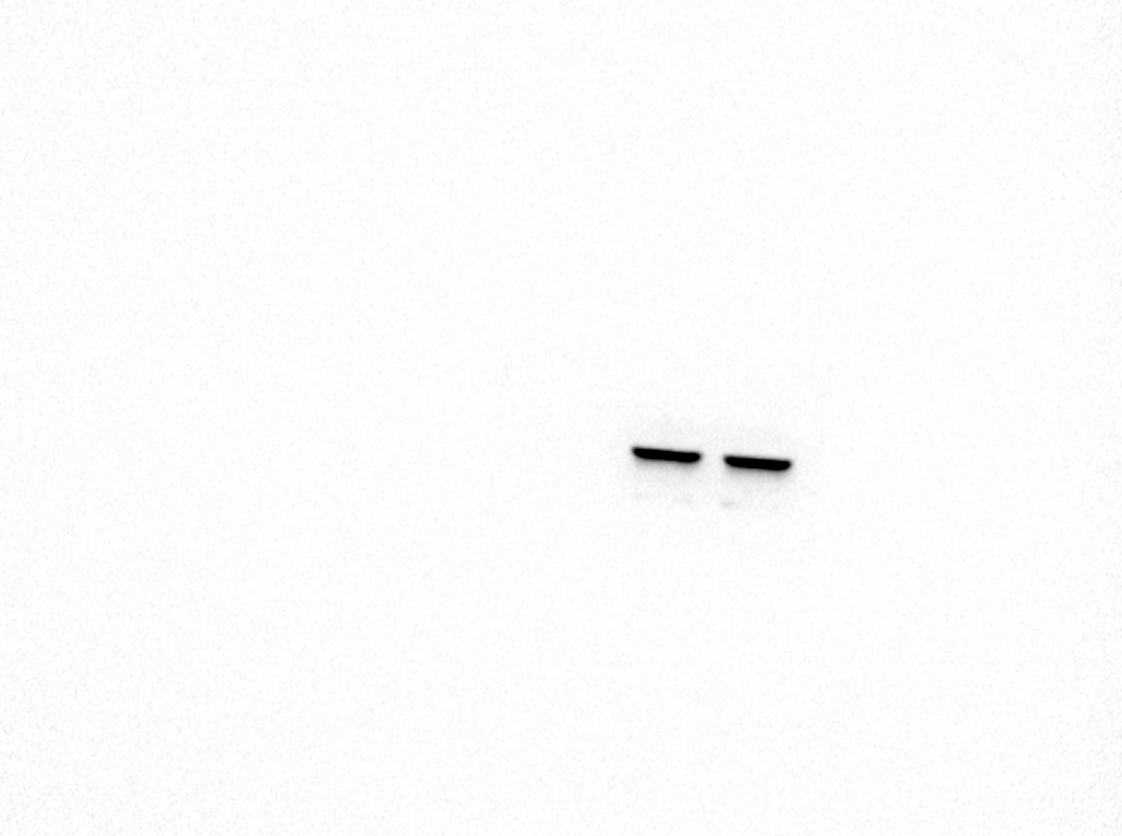

Supplement: Supplemental Information 2 — NMRAL2P overexpression plasmids (NMRAL2P-OE) or NMRAL2P knockdown (NMRAL2P-ASO) and their corresponding negative control groups (Vector or NC) were transferred into TU177 and AMC-HN-8 cells. 48 h later, the expression of ENO1 was detected. The image of protein ENO1 blot after NMRAL2P-oe or NMRAL2P-ASO were transferred into TU177 and AMC-HN-8 cells. [file peerj-11-16140-s002.zip › Overexpression and knock-down verification/WB Overexpression NMRAL2P/TU177 a┬-actin_Exposure_8.0sec.png]

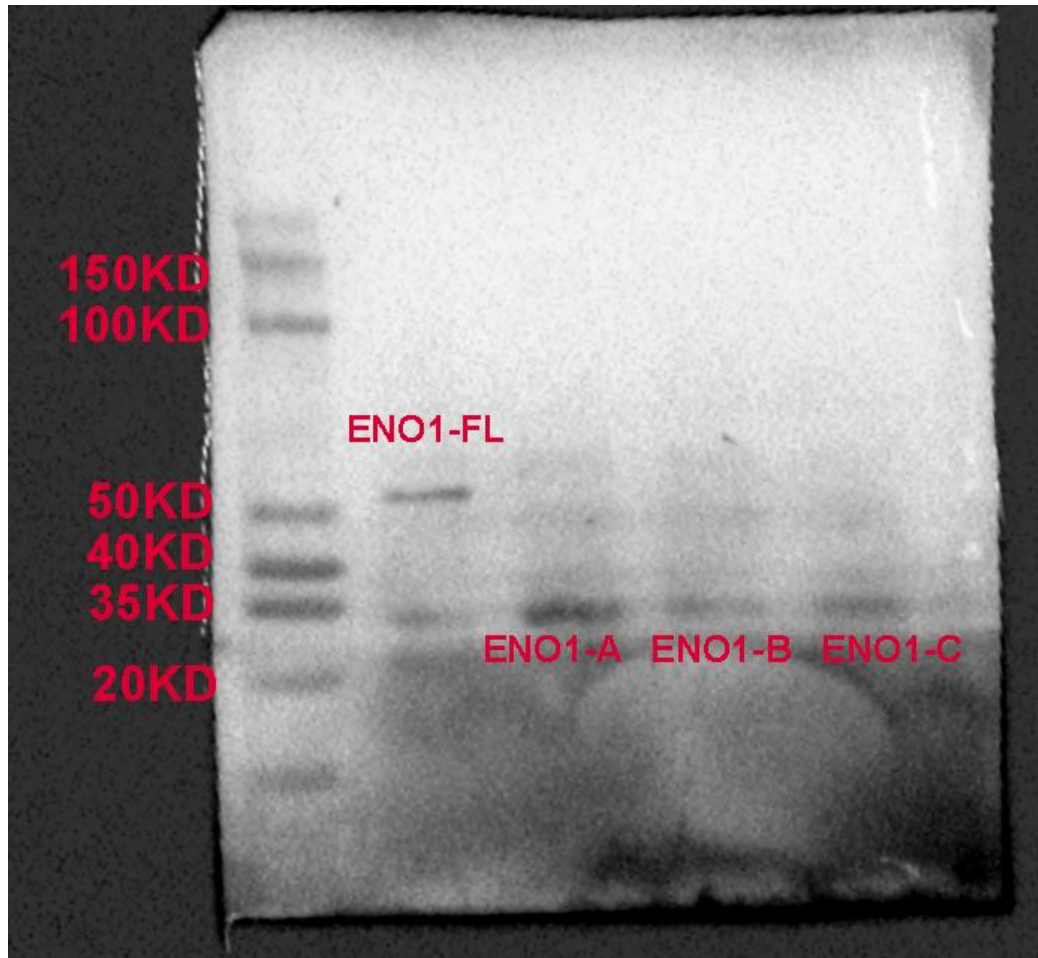

Supplementary Figure S1: Verifying ENO1 truncation efficiency by western blot

Supplement: Supplemental Information 7 [file peerj-11-16140-s007.pdf]

Project: Untitled.sqd Contig 4

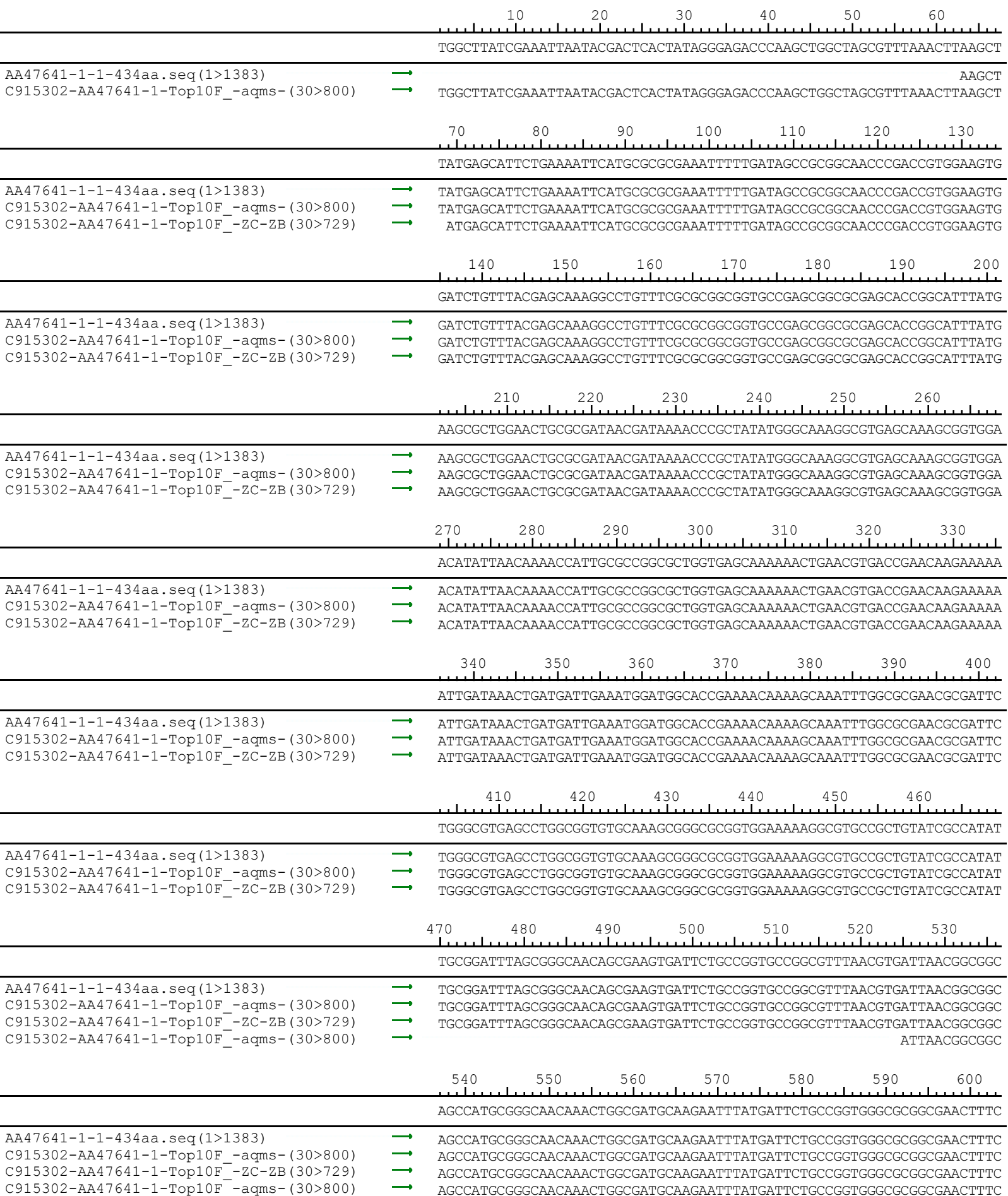

Project: Untitled.sqd Contig 4

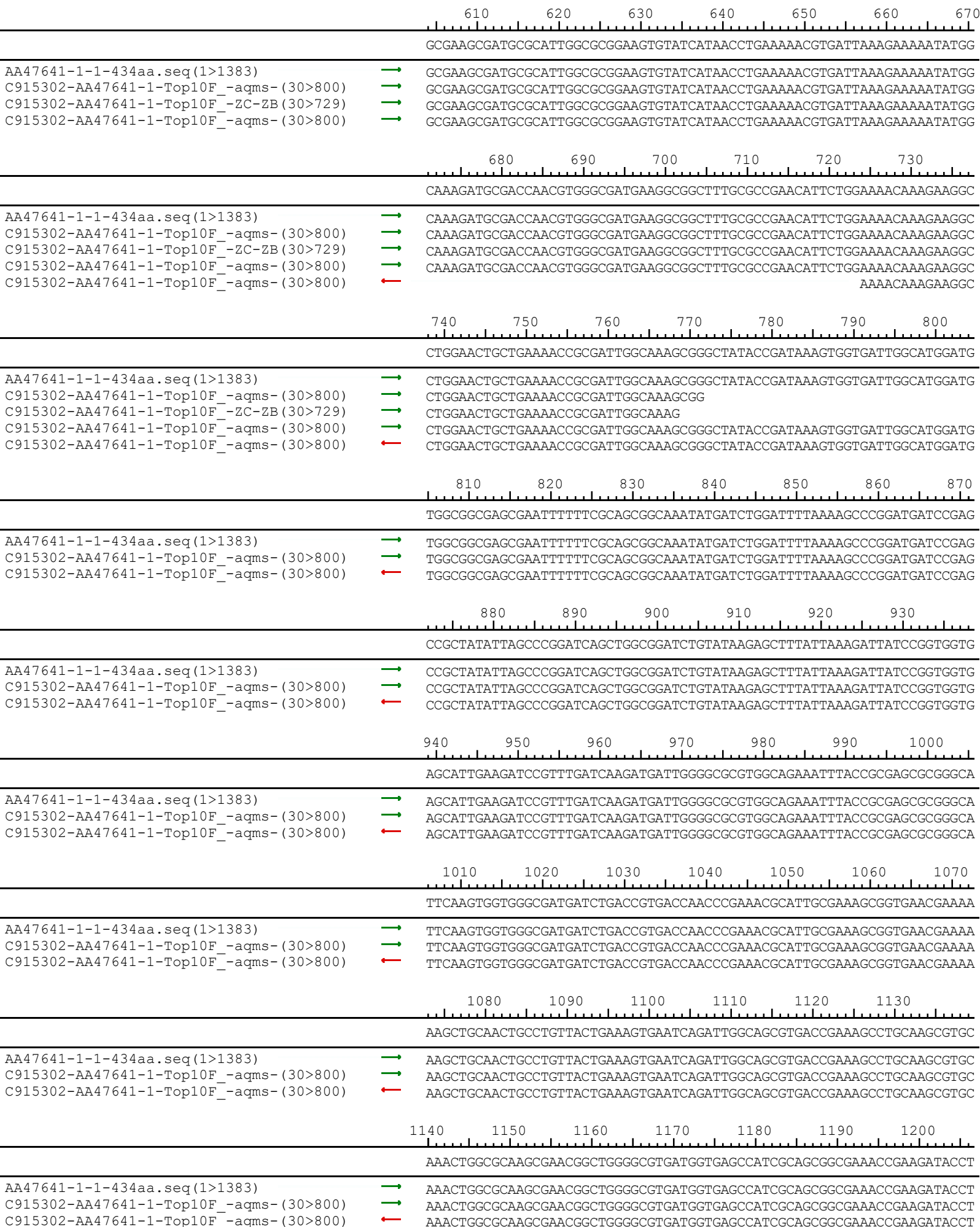

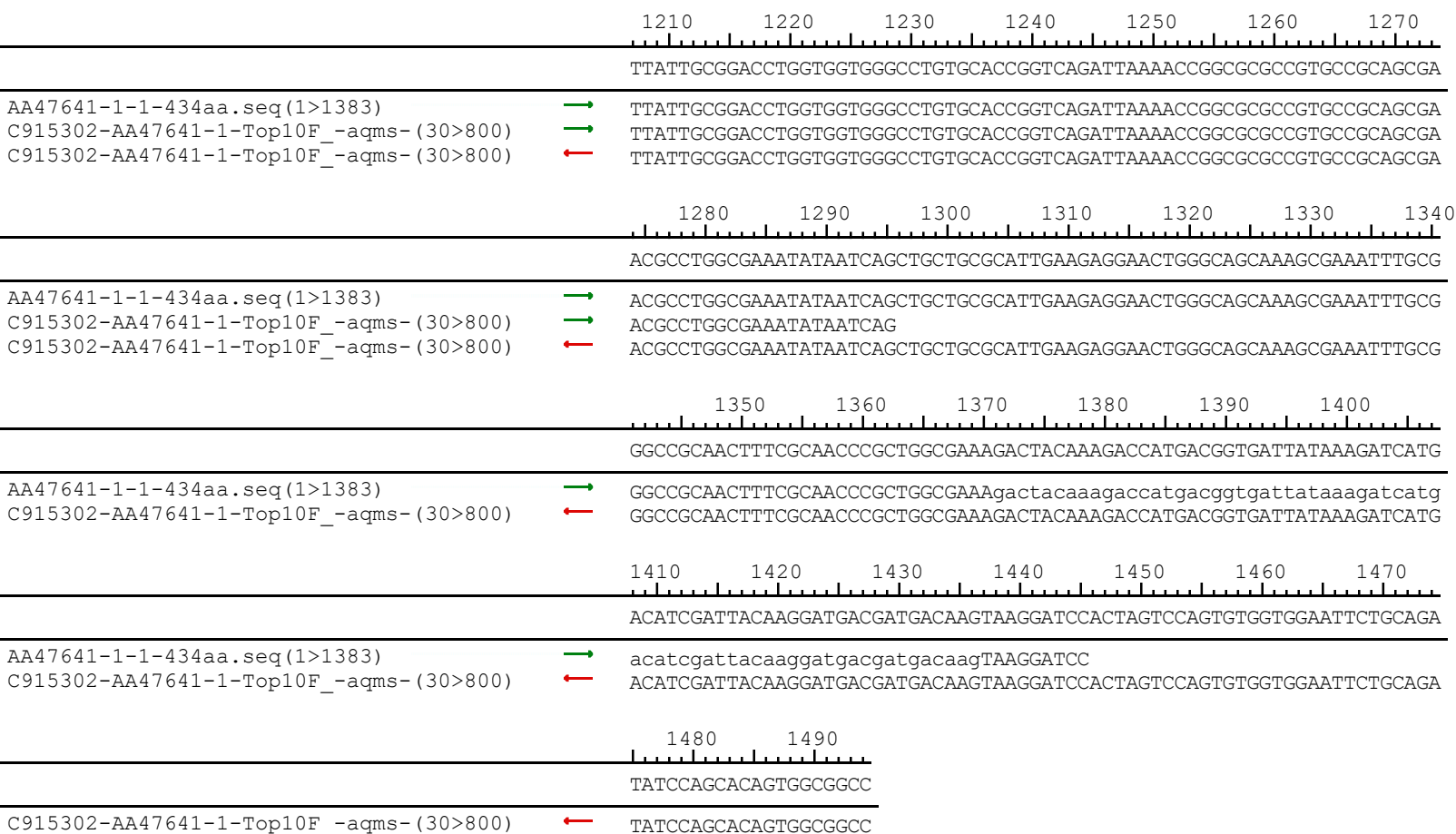

Supplement: Supplemental Information 13 [file peerj-11-16140-s013.zip › Supplementary file 3/Fig4C Sequencing result 1-434aa in pcDNA3.1.pdf]

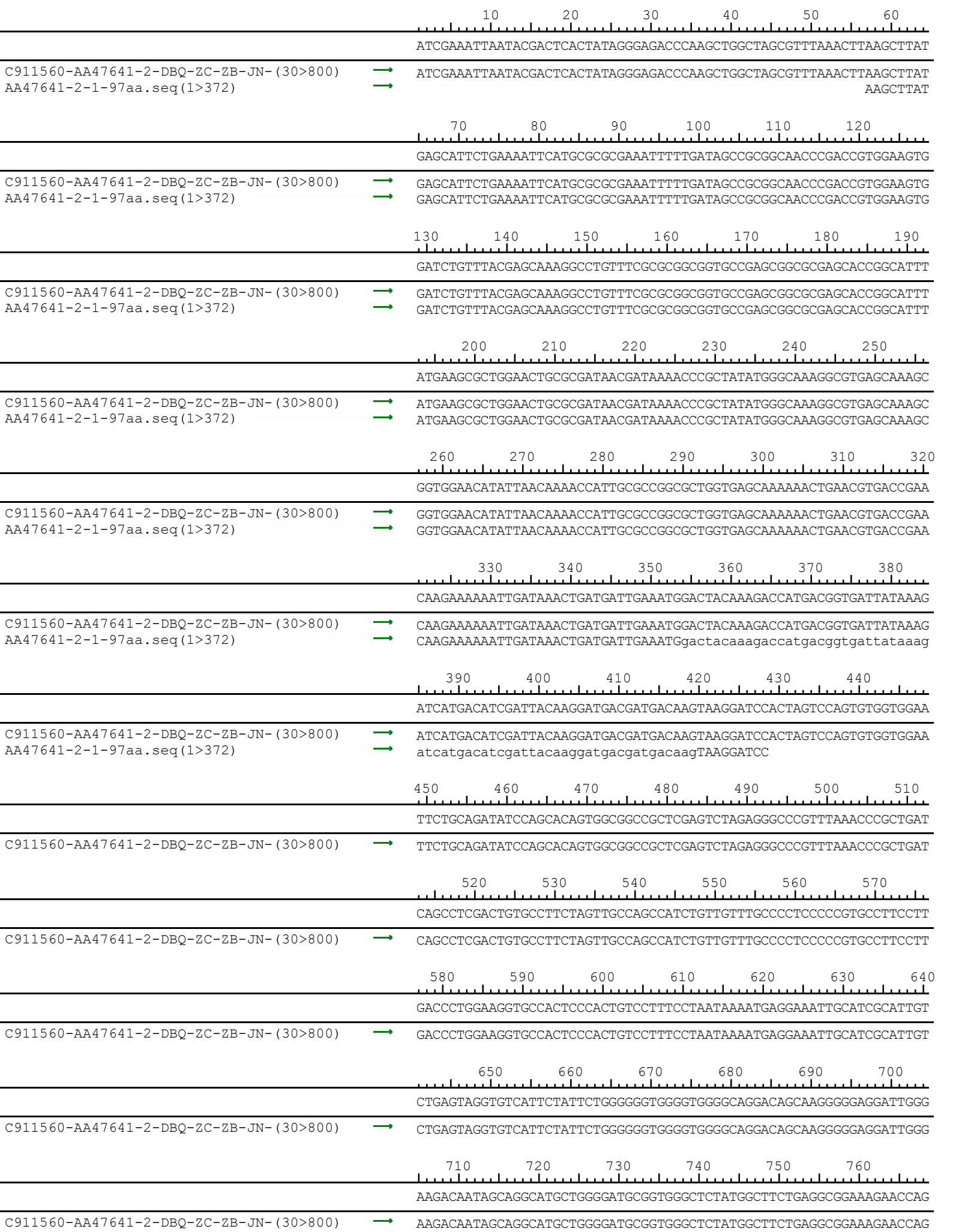

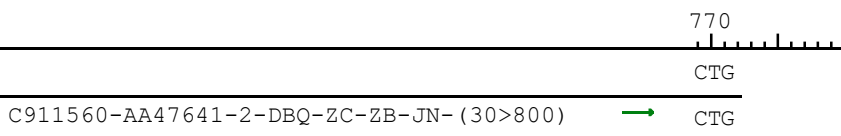

Supplement: Supplemental Information 13 [file peerj-11-16140-s013.zip › Supplementary file 3/Fig4C Sequencing result 1-97aa in pcDNA3.1.pdf]

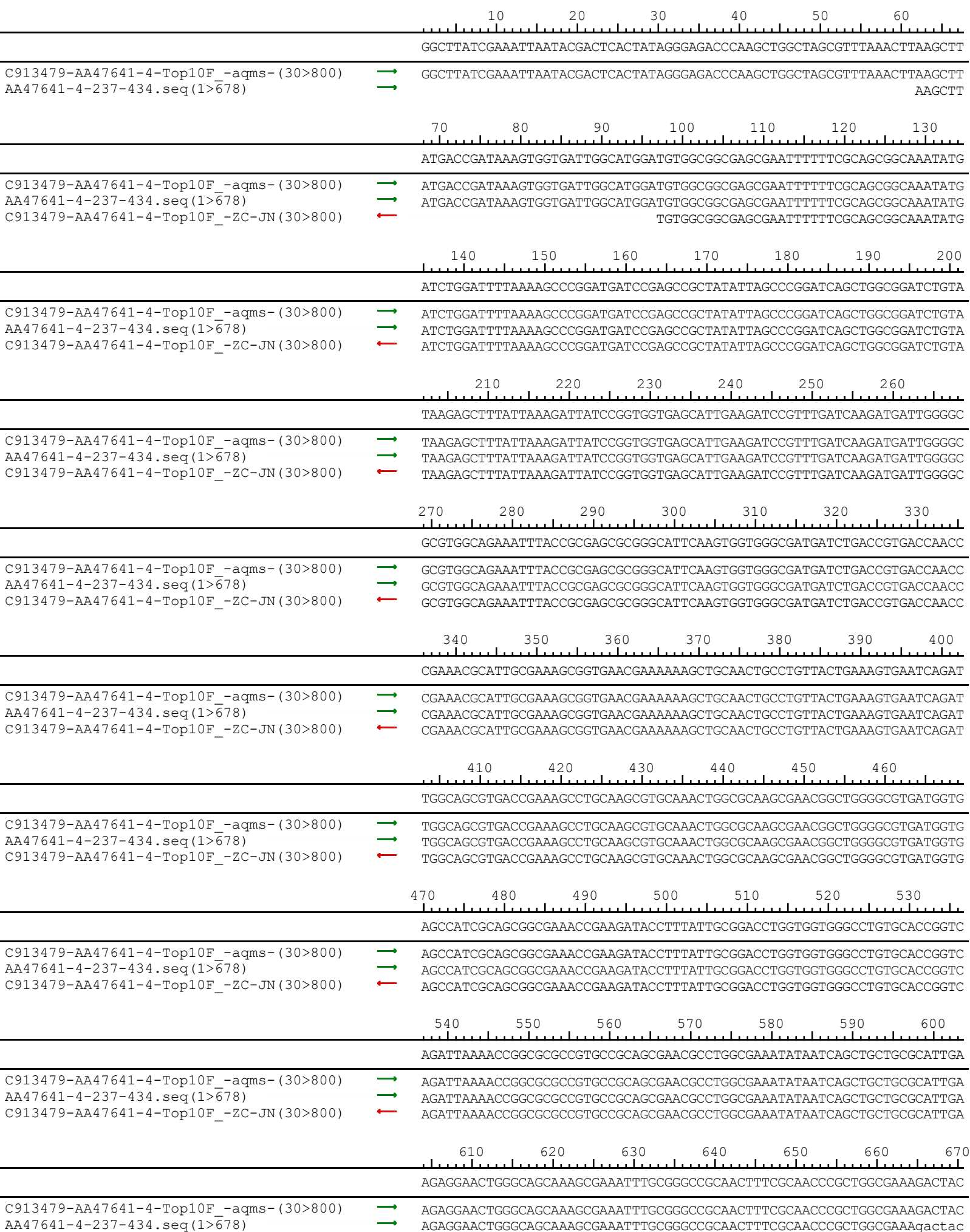

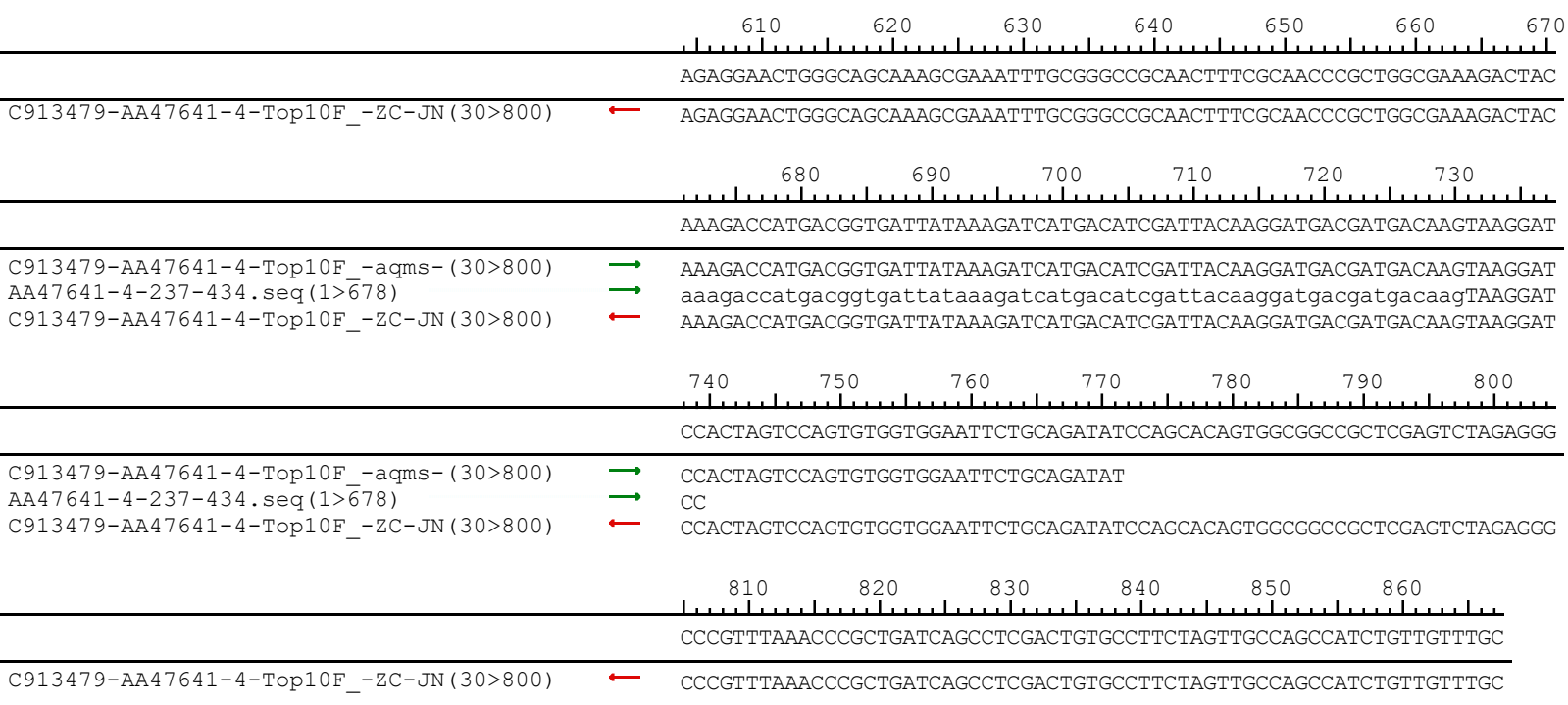

Supplement: Supplemental Information 13 [file peerj-11-16140-s013.zip › Supplementary file 3/Fig4C Sequencing result 237-434aa in pcDNA3.1.pdf]

Project: Untitled.sqd Contig 4

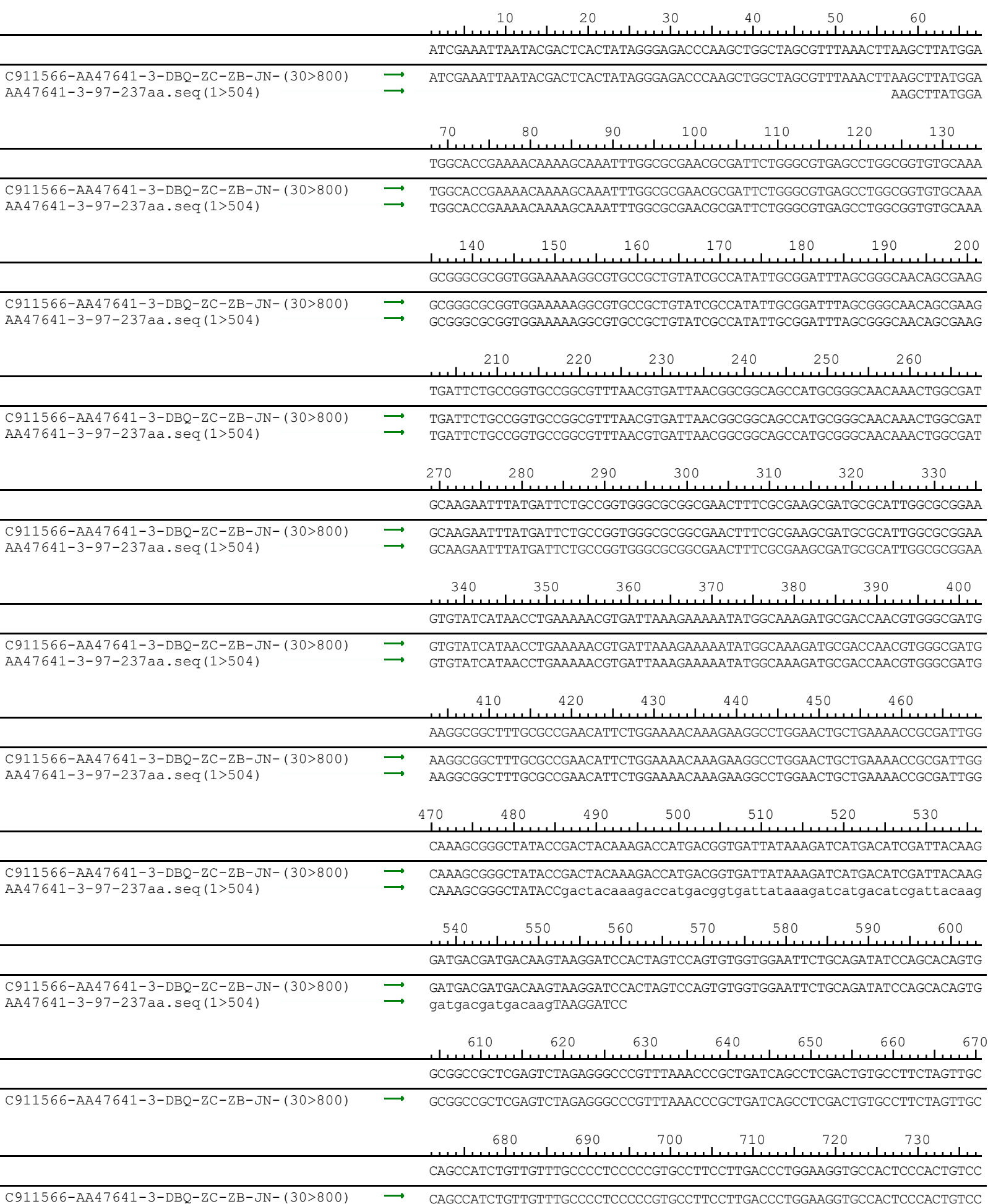

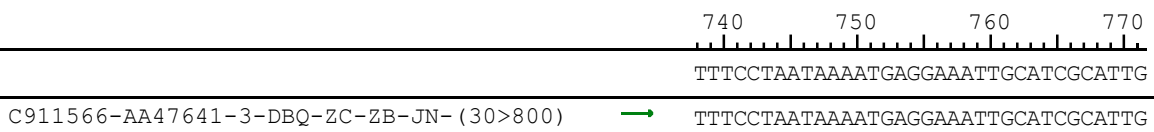

Supplement: Supplemental Information 13 [file peerj-11-16140-s013.zip › Supplementary file 3/Fig4C Sequencing result 97-237aa in pcDNA3.1.pdf]

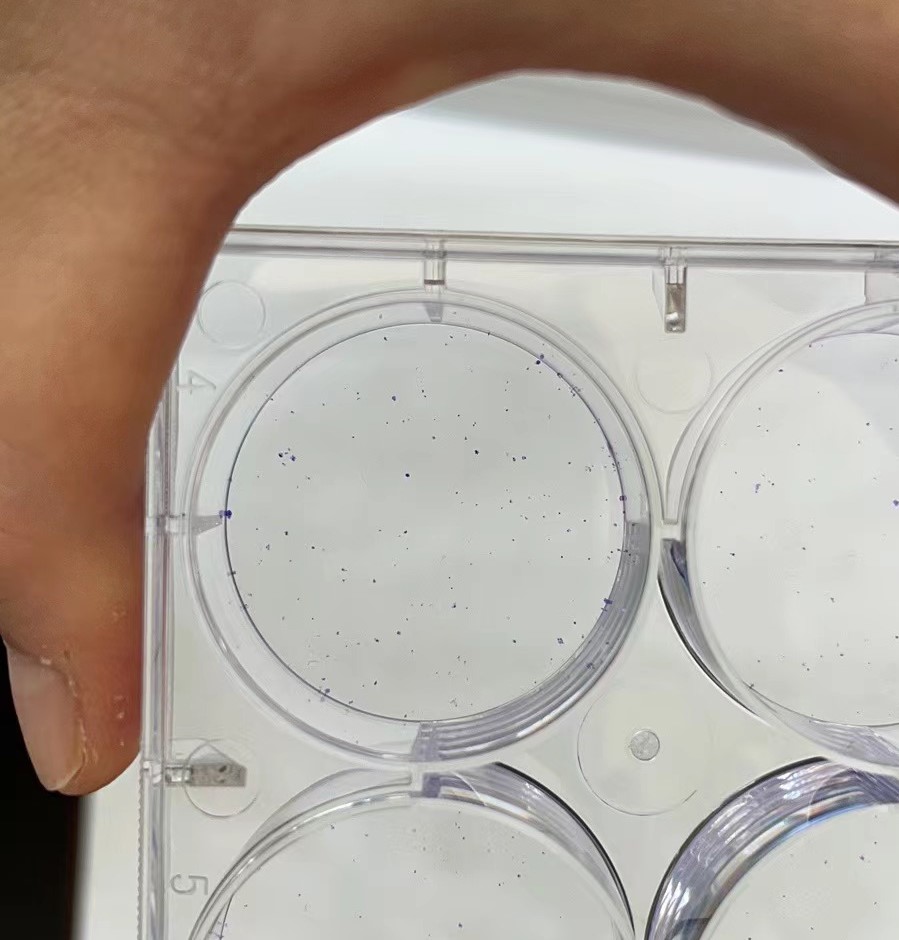

Supplement: Supplemental Information 15 [file peerj-11-16140-s015.zip › Raw data of supplementary assays/clone formation assay Fig.S2E-F;FigS3E-F/AMC-HN-8 pGenesil-1 Supplementary Figure S2.jpg]

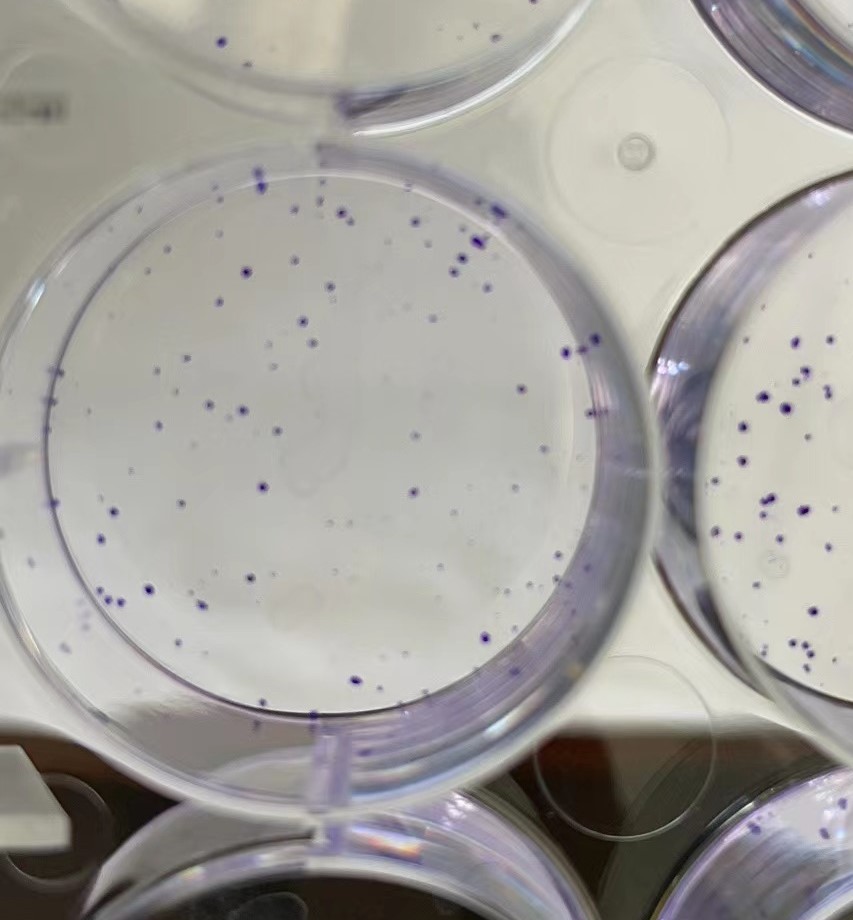

Supplement: Supplemental Information 15 [file peerj-11-16140-s015.zip › Raw data of supplementary assays/clone formation assay Fig.S2E-F;FigS3E-F/AMC-HN-8 pGenesil-1 Supplementary Figure S3.jpg]

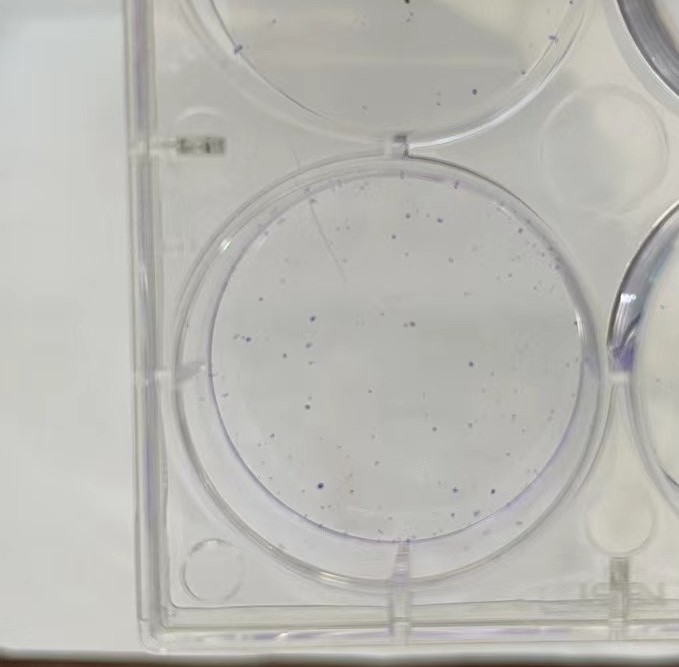

Supplement: Supplemental Information 15 [file peerj-11-16140-s015.zip › Raw data of supplementary assays/clone formation assay Fig.S2E-F;FigS3E-F/AMC-HN-8 shENO1 Supplementary Figure S2.jpg]

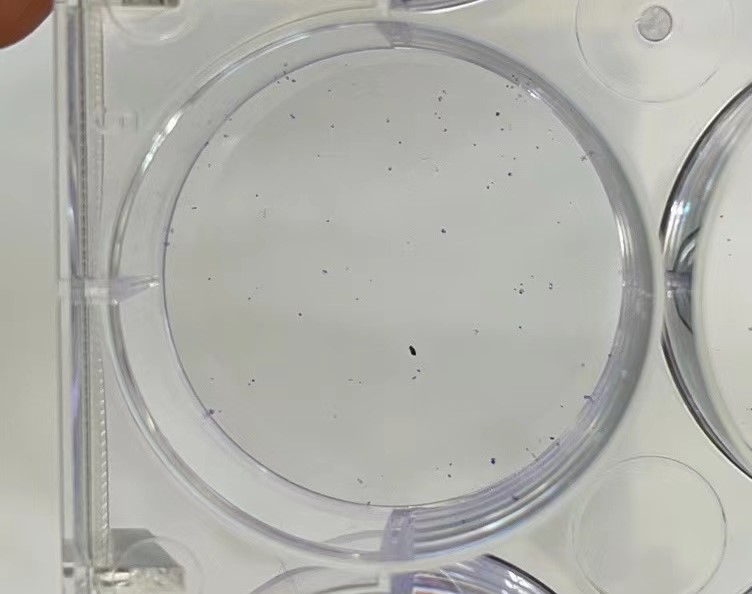

Supplement: Supplemental Information 15 [file peerj-11-16140-s015.zip › Raw data of supplementary assays/clone formation assay Fig.S2E-F;FigS3E-F/AMC-HN-8 shGPX2 Supplementary Figure S2.jpg]

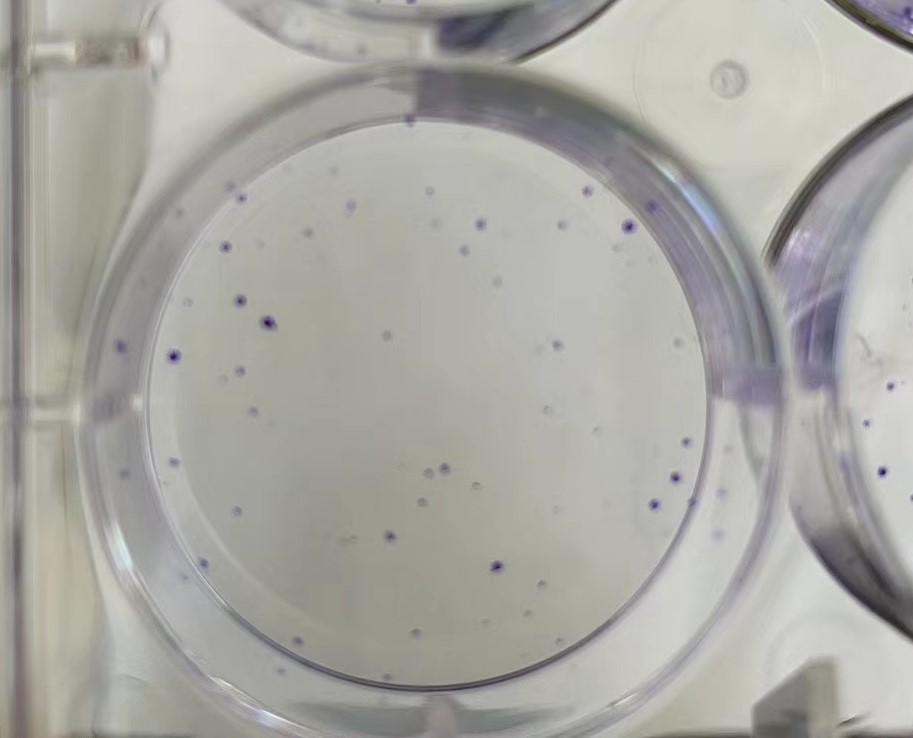

Supplement: Supplemental Information 15 [file peerj-11-16140-s015.zip › Raw data of supplementary assays/clone formation assay Fig.S2E-F;FigS3E-F/AMC-HN-8 shGPX2 Supplementary Figure S3 .jpg]

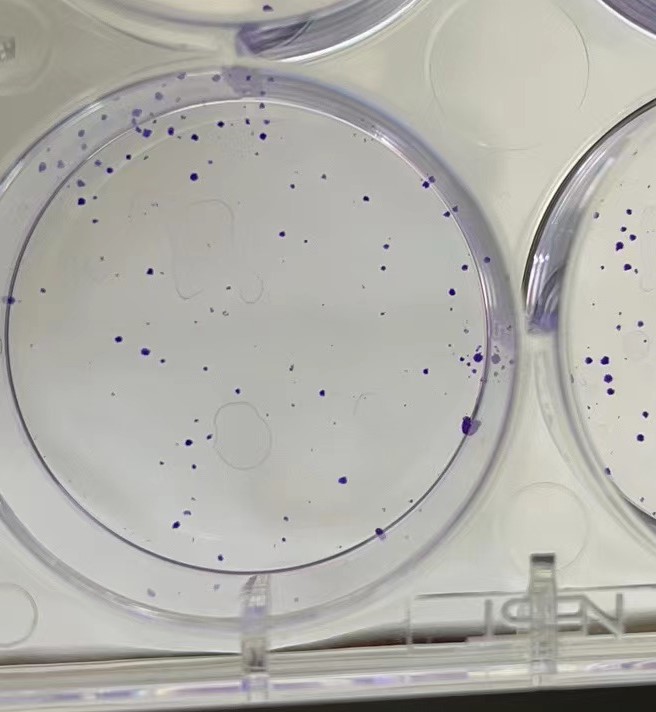

Supplement: Supplemental Information 15 [file peerj-11-16140-s015.zip › Raw data of supplementary assays/clone formation assay Fig.S2E-F;FigS3E-F/AMC-HN-8 shGPX2+Tempol Supplementary Figure S3.jpg]

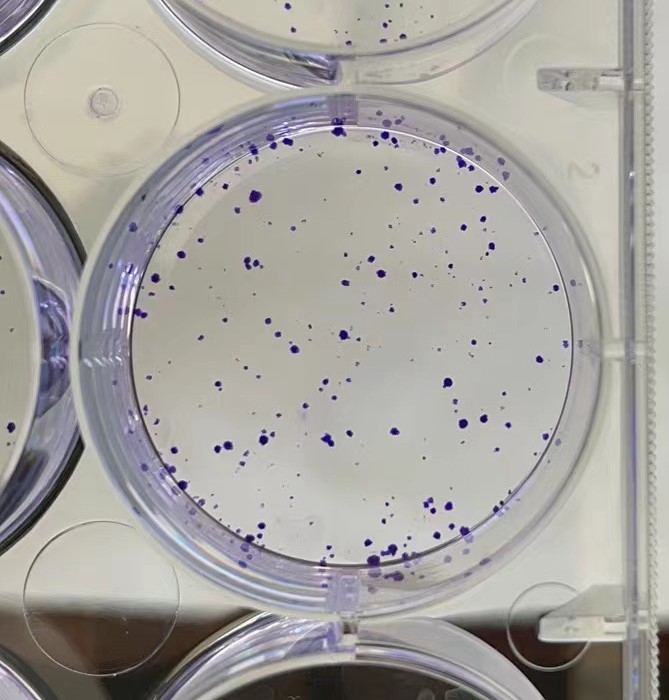

Supplement: Supplemental Information 15 [file peerj-11-16140-s015.zip › Raw data of supplementary assays/clone formation assay Fig.S2E-F;FigS3E-F/TU177 pGenesil-1 Supplementary Figure S2.jpg]

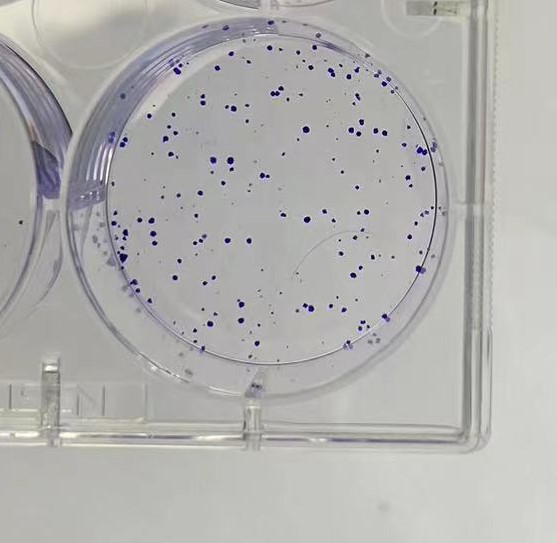

Supplement: Supplemental Information 15 [file peerj-11-16140-s015.zip › Raw data of supplementary assays/clone formation assay Fig.S2E-F;FigS3E-F/TU177 pGenesil-1 Supplementary Figure S3.jpg]

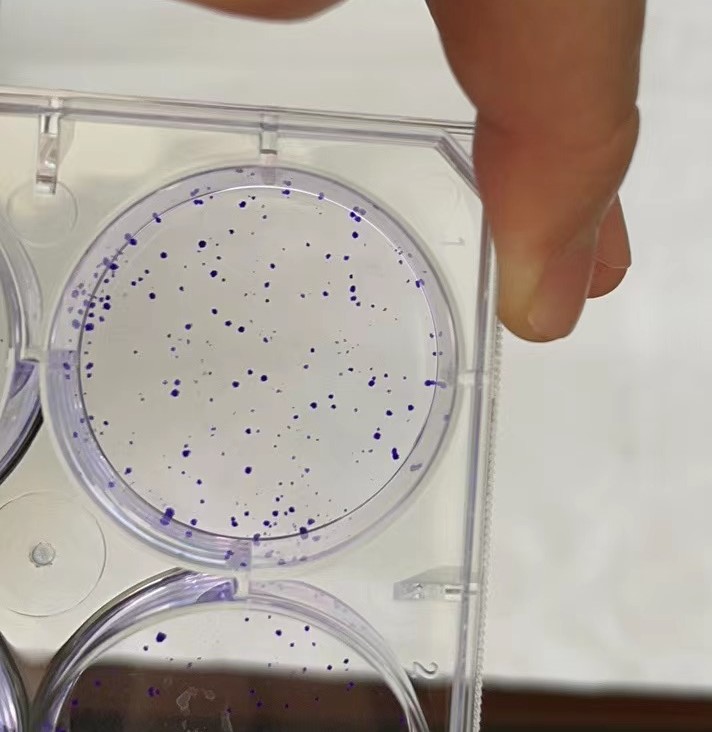

Supplement: Supplemental Information 15 [file peerj-11-16140-s015.zip › Raw data of supplementary assays/clone formation assay Fig.S2E-F;FigS3E-F/TU177 shENO1 Supplementary Figure S2.jpg]

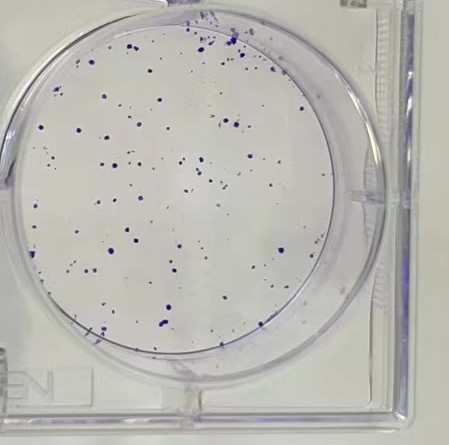

Supplement: Supplemental Information 15 [file peerj-11-16140-s015.zip › Raw data of supplementary assays/clone formation assay Fig.S2E-F;FigS3E-F/TU177 shGPX2 Supplementary Figure S2.jpg]

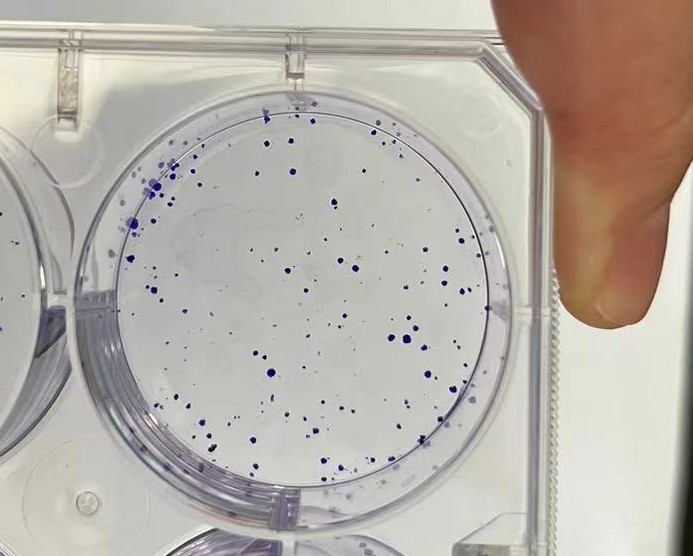

Supplement: Supplemental Information 15 [file peerj-11-16140-s015.zip › Raw data of supplementary assays/clone formation assay Fig.S2E-F;FigS3E-F/TU177 shGPX2 Supplementary Figure S3 .jpg]

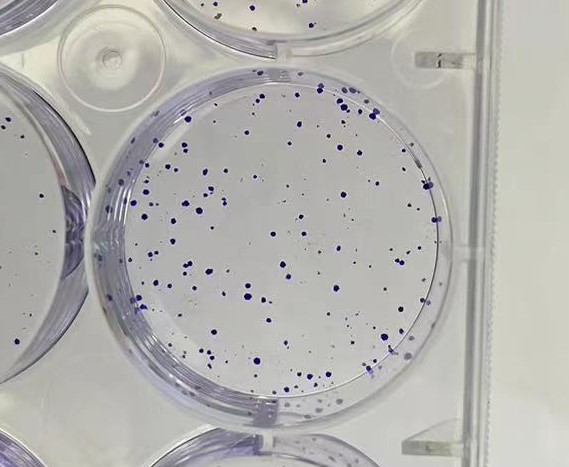

Supplement: Supplemental Information 15 [file peerj-11-16140-s015.zip › Raw data of supplementary assays/clone formation assay Fig.S2E-F;FigS3E-F/TU177 shGPX2+Tempol Supplementary Figure S3.jpg]
