# Supplementary material for: Long non-coding RNA NMRAL2P promotes glycolysis and reduces ROS in head and neck tumors by interacting with the ENO1 protein and promoting GPX2 transcription
Source: PeerJ. 2023 Oct 2;11:e16140. doi: 10.7717/peerj.16140 (PMC10552744; doi:10.7717/peerj.16140)
Supplement: Supplemental Information 5 [file peerj-11-16140-s005.docx]

Supplementary table S1:The sequence of primers and shRNA.

NMRAL2P (forward): 5ʹ- GCTGATATTGGAGCAGCCGT-3ʹ

NMRAL2P (reverse): 5ʹ- GCTTCCGGGGTAATCTTTGC -3ʹ

18S RNA(forward): 5ʹ- GTAACCCGTTGAACCCCATT-3ʹ

18S RNA(reverse): 5ʹ- CCATCCAATCGGTAGTAGCG-3ʹ

U6 (forward): 5ʹ-CTCGCTTCGGCAGCACA-3ʹ

U6 (reverse): 5ʹ-AACGCTTCACGAATTTGCGT-3ʹ

GAPDH(forward): 5ʹ- AGGTGAAGGTCGGAGTCAACG -3ʹ

GAPDH(reverse): 5ʹ-AGGGGTCATTGATGGCAACA-3ʹ

ENO1(forward): 5ʹ- GCCGTGAACGAGAAGTCCTG-3ʹ

ENO1 (reverse): 5ʹ- ACGCCTGAAGAGACTCGGT -3ʹ

GPX2 (forward): 5ʹ- GGTAGATTTCAATACGTTCCGGG -3ʹ

GPX2 (reverse): 5ʹ- TGACAGTTCTCCTGATGTCCAAA -3ʹ

NMRAL2P-ASO：CCACTAATGGGAGGGCAGAT

shENO1: GAATGTCATCAAGGAGAAATA

shGPX2: CCGATCCCAAGCTCATCATTT
