# Supplementary material for: Long non-coding RNA NMRAL2P promotes glycolysis and reduces ROS in head and neck tumors by interacting with the ENO1 protein and promoting GPX2 transcription
Source: PeerJ. 2023 Oct 2;11:e16140. doi: 10.7717/peerj.16140 (PMC10552744; doi:10.7717/peerj.16140)
Supplement: Supplemental Information 6 [file peerj-11-16140-s006.docx]

Supplementary table S2: 1399 mRNAs associated with oxidative stress

| gene |
| --- |
| KIT |
| NDUFA13 |
| MAPK13 |
| TNFSF4 |
| LEPQTL1 |
| MIR185 |
| ALS3 |
| ALS7 |
| OXTR |
| DHFR |
| ACACA |
| GPX8 |
| BGLAP |
| TAZ |
| MAPK12 |
| YBX1 |
| SFXN4 |
| SLC4A1 |
| HPSE |
| RPTOR |
| MTA1 |
| CD274 |
| ENDOG |
| BMP4 |
| MTTP |
| TCF7L2 |
| TLR6 |
| VDR |
| CPQ |
| NFU1 |
| GADD45G |
| FMO4 |
| BBC3 |
| AREG |
| HK1 |
| PIK3R2 |
| ATG5 |
| POU5F1 |
| SIRT6 |
| MIR214 |
| JAZF1 |
| FADD |
| BCL2A1 |
| IL11 |
| SELENOT |
| MAP3K11 |
| CYP4F2 |
| MIR24-2 |
| ASPA |
| ROCK1 |
| NEDD8 |
| GAA |
| ATP5PD |
| PEPD |
| CAPN2 |
| PTPRC |
| CYP20A1 |
| MKI67 |
| SORL1 |
| MIR210 |
| SORD |
| MUC5AC |
| ALDH3A2 |
| CHCHD2 |
| PLD1 |
| C5 |
| MIR222 |
| TNFRSF11B |
| TNFRSF10A |
| FABP1 |
| PFKM |
| PYCR2 |
| IL6R |
| SUMO1 |
| TEK |
| SLC7A1 |
| FIG4 |
| GLT8D1 |
| LBR |
| CXCL16 |
| OSM |
| GRIN1 |
| DYRK1A |
| MIR181A2 |
| DNM2 |
| TACO1 |
| GIGYF2 |
| C5AR1 |
| ALDH3B1 |
| CCR7 |
| MUC1 |
| CANX |
| ADH1A |
| FRZB |
| EEF2 |
| ZFAND1 |
| UBQLN4 |
| PKD1 |
| MIR200C |
| TNIP1 |
| SCN4B |
| IL16 |
| H3C14 |
| ARNT |
| CYP19A1 |
| SDC1 |
| CACNA2D1 |
| AQP1 |
| CDC25C |
| ACTN4 |
| BSG |
| LYRM4 |
| KL |
| ANXA11 |
| AVP |
| ABCC3 |
| MRAP |
| FANCD2 |
| FIS1 |
| PGAM5 |
| CAMKK2 |
| CFAP410 |
| VIPR1 |
| ADRB3 |
| RTN4 |
| MIR19A |
| FAAH |
| GLA |
| PDE4A |
| TARS2 |
| CX3CR1 |
| NOD2 |
| SFTPB |
| LANCL1 |
| CD38 |
| FECH |
| TPPP3 |
| IL1RAPL2 |
| TP53INP1 |
| ENC1 |
| PEX5 |
| IKBKG |
| FYN |
| ABCG2 |
| MIR199A1 |
| UCN2 |
| CD46 |
| KIAA0319L |
| MIR184 |
| CXCL2 |
| GPX5 |
| CD28 |
| YAP1 |
| GADD45B |
| EIF2AK4 |
| NEK1 |
| HDAC9 |
| ACAD8 |
| MIR203A |
| ADAM10 |
| MT-CO3 |
| RAG2 |
| C3 |
| HRH2 |
| VEGFC |
| SLC8A1 |
| MRPS34 |
| FCGR3A |
| IL12B |
| CIITA |
| SIAH1 |
| MIR23B |
| SET |
| MIR92A1 |
| MMD |
| ITGA2 |
| TLR5 |
| PHYH |
| HSPG2 |
| PRDM10 |
| RBP4 |
| DYNC1H1 |
| HPRT1 |
| SPARC |
| SELENOK |
| MAPK8IP1 |
| TFEB |
| TAT |
| HDAC2 |
| VNN1 |
| MIR142 |
| GDF15 |
| SORCS2 |
| MIR152 |
| SELENOP |
| ADSL |
| NEFL |
| HPX |
| OTC |
| LCAT |
| RYR3 |
| ISG15 |
| MAP3K1 |
| BCR |
| SLC25A1 |
| EPHA4 |
| MAPKAPK5 |
| ACTG1 |
| CLIC1 |
| SLC7A11 |
| TLR8 |
| NCF4 |
| DGKQ |
| IFNAR1 |
| MMP8 |
| CDKN2B |
| TOP1 |
| ACO2 |
| MIR25 |
| CUL3 |
| DUOX1 |
| CAMK4 |
| CUL1 |
| CYB5A |
| IGF2BP2 |
| UBE2D2 |
| FAM120A |
| AQP4 |
| PDGFB |
| MIR27A |
| KCNE2 |
| HAMP |
| BACH2 |
| PPP5C |
| AURKA |
| TLR7 |
| PTPA |
| WRN |
| TTPA |
| RARA |
| UCN |
| PKP2 |
| KCNT1 |
| ATXN8OS |
| PARK10 |
| PARK16 |
| PARK21 |
| MGMT |
| LIN28B |
| SMPD1 |
| GRM1 |
| DMPK |
| PYCR1 |
| UTRN |
| MIR148B |
| FOXJ1 |
| MIR144 |
| CCR5 |
| SLC40A1 |
| ITGB3 |
| CCL11 |
| MECOM |
| MIR20A |
| DUSP19 |
| VASP |
| HAO1 |
| MIR9-1 |
| FH |
| CYP21A2 |
| CACNA1A |
| BRAF |
| NOSIP |
| KLRK1 |
| ESR2 |
| PIK3C3 |
| VTN |
| IL33 |
| CXCR1 |
| PRKCG |
| TALDO1 |
| DECR1 |
| NR1H2 |
| HBEGF |
| AKAP9 |
| MIR34C |
| MSR1 |
| RPA1 |
| RCAN1 |
| FTH1 |
| CYP11A1 |
| CHGA |
| PLCB1 |
| SENP3 |
| RRM2B |
| PDK1 |
| SNCB |
| MIR107 |
| MIR181C |
| CAPN3 |
| RNASE3 |
| PRPH |
| CFI |
| LAMP1 |
| BLOC1S1 |
| PML |
| CCK |
| TIMP2 |
| TRAF2 |
| STIP1 |
| NOSTRIN |
| MYLK |
| GSTM5 |
| TPK1 |
| SLC2A4 |
| HLA-A |
| UNG |
| IL3 |
| RORA |
| MDH1 |
| RUNX2 |
| SCARA3 |
| PDIA3 |
| DDC |
| GSTM4 |
| PIGA |
| BRCA2 |
| UNC13A |
| VPS13C |
| TNFSF11 |
| CYP2C8 |
| SERPINF1 |
| DYSF |
| PSIP1 |
| TPI1 |
| PDGFRB |
| CAMK2G |
| EPHX2 |
| CR1 |
| CYP3A5 |
| AGRN |
| NR2C2 |
| XRCC6 |
| PPP3CA |
| UBQLN1 |
| FOXO4 |
| MAPK7 |
| NES |
| FTL |
| BACH1 |
| SCGB1A1 |
| FMO2 |
| APC |
| MYD88 |
| HERPUD1 |
| CYP17A1 |
| TYRP1 |
| XRCC5 |
| FKBP1B |
| MMP14 |
| TMEM161A |
| ALOX15 |
| SOX2 |
| ASL |
| CCNF |
| DIABLO |
| MMP7 |
| TRPV4 |
| PGK1 |
| NDUFA1 |
| IL23A |
| ATXN1 |
| JUNB |
| HSPA14 |
| CD86 |
| GAL |
| FGF7 |
| SCP2 |
| BCL6 |
| SULT1A3 |
| GADD45A |
| GSN |
| MDH2 |
| IL2RB |
| ABCC8 |
| PPIG |
| MCU |
| MLYCD |
| CHMP2B |
| UBC |
| MSN |
| PTPN3 |
| NTF4 |
| SLC19A3 |
| CCNB1 |
| FMR1 |
| HSPA6 |
| HK2 |
| SELL |
| PRKCZ |
| PTGIS |
| ZC3H12A |
| AHR |
| GLS |
| PPOX |
| NCAM1 |
| RETN |
| B2M |
| CD55 |
| VKORC1L1 |
| SLC25A27 |
| EDNRB |
| PF4 |
| SMAD2 |
| SOCS1 |
| BLK |
| PYGM |
| CDK6 |
| MICB |
| PPARD |
| UBQLN2 |
| HYOU1 |
| TACR1 |
| BDKRB2 |
| ADRB1 |
| CTTN |
| DEPDC5 |
| MIR106B |
| NQO2 |
| ABCC2 |
| SREBF1 |
| SERPINH1 |
| H2BC21 |
| LPA |
| VCL |
| MIR145 |
| MIR143 |
| FASN |
| CASP4 |
| CD34 |
| KRT18 |
| CTSG |
| SLC11A2 |
| ODC1 |
| TNFRSF10B |
| CHEK1 |
| SFTPD |
| CD69 |
| PDLIM4 |
| GH1 |
| LPL |
| HNF1A |
| SCARB1 |
| PTX3 |
| RAD51 |
| EZH2 |
| COX15 |
| LGALS1 |
| GRIN2A |
| VAPB |
| ELAVL1 |
| HRH1 |
| ATR |
| PNKP |
| GFER |
| CDH5 |
| MIR24-1 |
| ABCA1 |
| DAXX |
| CDH2 |
| GZMB |
| CALB2 |
| MUTYH |
| ERCC8 |
| DDAH1 |
| NDUFS7 |
| NDUFS6 |
| ERO1A |
| DSP |
| NDUFA10 |
| SUMO2 |
| NRAS |
| IGF2BP1 |
| CXCL9 |
| SLC18A2 |
| PIK3C2A |
| BCL2L11 |
| STK4 |
| HNF4A |
| ALOX12 |
| GLS2 |
| CFH |
| F8 |
| DNAJB1 |
| TBK1 |
| ITIH4 |
| LRPPRC |
| XRCC1 |
| TP73 |
| ANGPT2 |
| AOX1 |
| NEAT1 |
| NME1 |
| DES |
| TRPA1 |
| TGFA |
| MAP3K7 |
| EPX |
| PLG |
| INSR |
| RHOD |
| GP1BA |
| LGALS3 |
| CYC1 |
| BAG3 |
| PARK12 |
| STAT4 |
| NR1H4 |
| DNAH8 |
| MT-ND6 |
| NDUFA9 |
| FCGR2B |
| CSK |
| STK24 |
| AR |
| GYG1 |
| PRKD2 |
| PPIA |
| CDK4 |
| TPT1 |
| COA8 |
| IRF5 |
| F5 |
| SCO2 |
| PKM |
| GLO1 |
| MYH6 |
| MME |
| FOXM1 |
| ACOX2 |
| JAK1 |
| H19 |
| NDRG1 |
| PEX11B |
| ERBB2 |
| FKBP5 |
| MIR133B |
| TPM1 |
| MIR221 |
| VHL |
| IL2RA |
| MSH2 |
| C4A |
| RAB5A |
| PLAUR |
| NEIL1 |
| SPR |
| LCK |
| MSRB3 |
| GLE1 |
| TAF15 |
| GFM2 |
| SETX |
| ADAMTS13 |
| GHRL |
| KCNMA1 |
| MIR125A |
| H2AX |
| FOXP3 |
| KRT8 |
| PLA2G2A |
| BRF2 |
| MIR200B |
| FKRP |
| ADPRS |
| H4-16 |
| ALAD |
| MIR181A1 |
| MT-ND4 |
| NDUFB9 |
| IGF2 |
| ACTN2 |
| ISCU |
| XIAP |
| CFLAR |
| GSTA2 |
| NLRP3 |
| QDPR |
| CASP2 |
| SRF |
| PKLR |
| TYMP |
| SCO1 |
| PTS |
| THBS1 |
| SYP |
| TYK2 |
| GRN |
| OPTN |
| GRIA1 |
| PIK3CB |
| IKBKB |
| CEBPB |
| CD80 |
| C4B |
| PPP1R15A |
| ACSL4 |
| EIF2B3 |
| PLA2G4A |
| TUBA1B |
| STK11 |
| MMP3 |
| GRM5 |
| DRD3 |
| SMARCA4 |
| NTRK1 |
| ECE1 |
| LYN |
| ELK1 |
| ALDH3A1 |
| PDCD1 |
| CCS |
| SIL1 |
| IL5 |
| PSEN2 |
| ARG2 |
| IDO1 |
| DYNLL1 |
| MTR |
| PDIA2 |
| SLPI |
| SLC1A1 |
| EIF2B4 |
| ADAM17 |
| CAMP |
| CYP2A6 |
| HDAC1 |
| CD79A |
| CYP27A1 |
| RXRA |
| PTPN1 |
| EP300 |
| CXCR3 |
| NAT2 |
| EDNRA |
| PFN1 |
| MT-ND3 |
| NTHL1 |
| KCNJ2 |
| NRG1 |
| CHCHD10 |
| BECN1 |
| CLEC4A |
| COQ2 |
| EIF4EBP1 |
| SGK1 |
| ANGPT1 |
| SOCS3 |
| UCP1 |
| MIR93 |
| AIF1 |
| IL15 |
| TRIM21 |
| LTA |
| NAMPT |
| MIR29A |
| MFN2 |
| CALM2 |
| TIA1 |
| RELA |
| NGFR |
| TRAP1 |
| HGF |
| GAP43 |
| MIR433 |
| SLC6A2 |
| CD4 |
| MYO9A |
| PENK |
| EPRS1 |
| CDH1 |
| CFTR |
| RPS6KA5 |
| PVALB |
| MRPL44 |
| CTSB |
| NTS |
| ERBB4 |
| EIF4G1 |
| KLF4 |
| CYP2B6 |
| ACE2 |
| SESN1 |
| NRF1 |
| PECAM1 |
| KLF2 |
| GSS |
| EIF2AK1 |
| FLT1 |
| RPS6KB1 |
| OXA1L |
| ATXN3 |
| HLA-B |
| ATF3 |
| RAF1 |
| MALAT1 |
| ADA |
| MAPK11 |
| GRIN2B |
| ANXA2 |
| COX6B1 |
| HMGCL |
| CXCR4 |
| CXCL10 |
| MMP13 |
| MIR122 |
| STK39 |
| GCLM |
| TLR3 |
| BAD |
| TBP |
| CCN2 |
| TJP1 |
| ALDH9A1 |
| BIRC5 |
| FCGR3B |
| PDGFRL |
| NLRP1 |
| KCNE1 |
| GAD1 |
| ENO2 |
| PEX12 |
| CDKN1B |
| GJA1 |
| HSPA1B |
| LOC111365141 |
| FGF1 |
| DRD4 |
| IRF1 |
| ATF2 |
| CDK1 |
| RNF112 |
| HMGCR |
| DHCR24 |
| NTRK2 |
| CXCL1 |
| AHSP |
| NDUFA6 |
| PLCG1 |
| IRAK1 |
| FMO1 |
| HSP90AB1 |
| ITGAL |
| AKT2 |
| FDXR |
| MIR126 |
| CCR6 |
| KIF1B |
| DSPP |
| CCNA2 |
| CPOX |
| ALDH1A1 |
| HSPB2 |
| CNTF |
| MT3 |
| SDHAF2 |
| EPAS1 |
| LONP1 |
| ABCC1 |
| SETD2 |
| E2F1 |
| MTFMT |
| CALB1 |
| MBP |
| S100A9 |
| NDUFS1 |
| IGF1R |
| CHUK |
| DNASE1 |
| IFNB1 |
| VIM |
| ANK2 |
| MAP2K7 |
| CR2 |
| DLG4 |
| DRD1 |
| PCNA |
| ADCY10 |
| PLAT |
| TSC1 |
| ELN |
| MBL2 |
| BMP2 |
| LCN2 |
| GLUD1 |
| TNFSF10 |
| DMD |
| MCL1 |
| NOL3 |
| CST3 |
| CBS |
| NDUFS8 |
| CHKA |
| DDAH2 |
| NTF3 |
| ACTB |
| PRKAB1 |
| STUB1 |
| FCGR2A |
| ITGB2 |
| MATR3 |
| APOB |
| STK25 |
| TLR9 |
| CSF2 |
| APAF1 |
| PDYN |
| OPRD1 |
| TNFAIP3 |
| ABCB1 |
| TGM2 |
| ACTA1 |
| MIR23A |
| GRB2 |
| HSD17B10 |
| MRPS14 |
| MIR132 |
| LTF |
| CCL4 |
| NDUFS2 |
| BTD |
| IFNA1 |
| RB1 |
| SMAD4 |
| MAP2K6 |
| SRXN1 |
| LPO |
| HBA1 |
| IRS1 |
| SLC17A5 |
| KCNQ1 |
| HTR2C |
| ENO1 |
| LAMP2 |
| ITGB1 |
| SERPINA1 |
| S100B |
| LOC110973015 |
| REST |
| PIK3R1 |
| CDC42 |
| CXCL12 |
| DAO |
| BAK1 |
| HCRT |
| ADORA2A |
| MTHFR |
| CSF3 |
| BRCA1 |
| GNAS |
| SIGMAR1 |
| IREB2 |
| TGFBR1 |
| CARS2 |
| CALM3 |
| MAP2 |
| MIF |
| CTSD |
| MAPKAPK3 |
| PAH |
| OXR1 |
| CACNB4 |
| PXN |
| HTR3A |
| IGF2R |
| CASP7 |
| GSTO2 |
| ANG |
| EPHA3 |
| PTPN22 |
| MRPS16 |
| GLUD2 |
| CREBBP |
| SLC25A13 |
| SIRT3 |
| GSTA4 |
| IDH2 |
| TOR1A |
| DCTN1 |
| MIR17 |
| C12orf65 |
| EPHX1 |
| TGFBR2 |
| BCHE |
| ERCC6 |
| NFE2L1 |
| EIF2AK2 |
| ADM |
| H2AC18 |
| COL2A1 |
| TRPV1 |
| VIP |
| SYK |
| A2M |
| ALOX5 |
| CD44 |
| IAPP |
| PRKCA |
| SNCAIP |
| S100A8 |
| NAGS |
| HNRNPA1 |
| ATP13A2 |
| SLC25A3 |
| DAPK1 |
| TTR |
| EIF4E |
| DLST |
| CRYAA |
| UBE2L3 |
| NEFH |
| G3BP1 |
| ADH1C |
| DNMT1 |
| MRPS22 |
| TFAM |
| OSGIN2 |
| CCL3 |
| SCN4A |
| UCHL1 |
| MIR195 |
| FGFR1 |
| OXT |
| MIR34A |
| AKR1A1 |
| OPA1 |
| VARS2 |
| SLC1A2 |
| CRHR1 |
| POR |
| PRKG1 |
| TGFB3 |
| KCNJ5 |
| BACE1 |
| SNTA1 |
| DRD5 |
| SERPINA3 |
| HSPA9 |
| SCN2A |
| AMPD1 |
| TNFRSF1B |
| ITPR1 |
| TSFM |
| MT-TK |
| MIR223 |
| TGFB2 |
| UCP3 |
| IDH1 |
| CSF1 |
| DNM1L |
| MET |
| TSC2 |
| PLA2G6 |
| PGD |
| KCNH2 |
| HMOX2 |
| PPIF |
| EHHADH |
| TXNRD2 |
| AKR1B1 |
| GSTM2 |
| MAP2K3 |
| NGB |
| MMP1 |
| NCF1 |
| MECP2 |
| NPPB |
| SIRT2 |
| STAT1 |
| ECHS1 |
| AOC3 |
| H6PD |
| FGF2 |
| GPX2 |
| GLRX2 |
| OSER1 |
| GSTM3 |
| KRIT1 |
| SDHAF1 |
| OPRM1 |
| SESN2 |
| APOH |
| VDAC1 |
| REN |
| SST |
| ADRB2 |
| NR3C2 |
| GSK3B |
| KRAS |
| MYC |
| ADH5 |
| HTR1A |
| FXN |
| NPPA |
| PTK2 |
| CPT1B |
| HLA-DRA |
| UGT1A1 |
| PRKD1 |
| CYB5R3 |
| GPX4 |
| GPT |
| FMO3 |
| F3 |
| TFRC |
| JAK2 |
| MT-TL1 |
| IL1R1 |
| MAP2K1 |
| MT-CO2 |
| ATP2A2 |
| PLA2G7 |
| CD40 |
| MAP2K4 |
| GSTO1 |
| TPH1 |
| BCL2L1 |
| ACHE |
| CRAT |
| GCLC |
| NFKBIA |
| SGCB |
| IL4 |
| UCP2 |
| NR4A2 |
| EGR1 |
| IL17A |
| EGR1 |
| LOC110806262 |
| TRMT10C |
| PNPT1 |
| CDKN2A |
| PTK2B |
| CCL5 |
| NPM1 |
| ETS1 |
| PRODH |
| ACO1 |
| EEF1A1 |
| OGDH |
| TAC1 |
| CASP1 |
| CYP11B2 |
| NDUFAF2 |
| PRKCD |
| SLC5A7 |
| SMAD3 |
| ENG |
| GLUL |
| HSD17B4 |
| RAC2 |
| MT-ND2 |
| PON3 |
| ATXN2 |
| PTEN |
| TSPO |
| ELANE |
| SDHC |
| TUFM |
| ERN1 |
| POMC |
| DBH |
| CYP2C9 |
| GPX7 |
| P4HB |
| FUS |
| MT-ND5 |
| FASLG |
| HLA-DRB1 |
| MIR155 |
| TF |
| CHKB |
| CDK2 |
| CD36 |
| HFE |
| ACP1 |
| GSTA1 |
| NOTCH1 |
| DUSP1 |
| PRDX4 |
| PTPN11 |
| AGTR1 |
| HSP90B1 |
| SLC18A3 |
| FN1 |
| PRKCB |
| CNR1 |
| TREM2 |
| NDUFV2 |
| GLRX |
| ITGAM |
| TECRL |
| IL1RN |
| CACNA1S |
| HTT |
| PLAU |
| TIMP1 |
| CDK5 |
| CALR |
| MGST1 |
| SUOX |
| PTGS1 |
| CYP2C19 |
| TREX1 |
| TTN |
| DLD |
| ADCYAP1 |
| CS |
| TXNIP |
| IL18 |
| GCDH |
| APOA1 |
| CYGB |
| CCND1 |
| PDHA1 |
| ASS1 |
| LRRK2 |
| HTR2A |
| DRD2 |
| SLC6A3 |
| MAPKAPK2 |
| ARG1 |
| GGT1 |
| SLC25A4 |
| NPY |
| GBA |
| BMP6 |
| HSPD1 |
| CYP1B1 |
| NOX1 |
| HP |
| LEP |
| BLVRB |
| NOS1AP |
| FARS2 |
| HRAS |
| CALCA |
| RAC1 |
| SNAP25 |
| PRKAA2 |
| PRKAA1 |
| EPO |
| MSRB2 |
| LOX |
| NDUFB8 |
| PDE5A |
| CRYAB |
| EGFR |
| RPS27A |
| MDM2 |
| THBD |
| NDUFV1 |
| CTLA4 |
| MIR22 |
| MAPK9 |
| NGF |
| IL2 |
| SRC |
| CREB1 |
| ATF6 |
| MIR146A |
| TLR2 |
| ATM |
| SDHD |
| IL1A |
| NDUFS3 |
| MT-ATP6 |
| MT-CYB |
| CDKN1A |
| OSGIN1 |
| SLC1A3 |
| ATF4 |
| GTPBP3 |
| RHOA |
| PPARA |
| ABL1 |
| KDR |
| SPP1 |
| ACAD9 |
| AGT |
| TRPM2 |
| GSTT1 |
| PIK3CA |
| PRDX1 |
| HMGB1 |
| CLU |
| PIK3CG |
| CDKN3 |
| HBB |
| NDUFA12 |
| SELP |
| ATP5F1A |
| SERPINE1 |
| C1QBP |
| PRDX3 |
| TXNRD1 |
| SLC22A5 |
| MSRB1 |
| AGER |
| PRL |
| LDLR |
| CTNNB1 |
| TRDN |
| CASQ2 |
| PC |
| CALM1 |
| ETFB |
| SHC1 |
| COMT |
| ANXA5 |
| MMP2 |
| TH |
| SELE |
| STAT3 |
| NUDT1 |
| MT-CO1 |
| MIR21 |
| EIF2S1 |
| EIF2AK3 |
| TNFRSF1A |
| TERT |
| IL13 |
| GDNF |
| SLC2A1 |
| FAS |
| SOD3 |
| MB |
| MT-ND1 |
| SDHA |
| CYP1A2 |
| SDHB |
| NR3C1 |
| MMP9 |
| TXN2 |
| OXSR1 |
| HSPA8 |
| C9orf72 |
| GFAP |
| IGF1 |
| TPO |
| EGF |
| MYH7 |
| MSRA |
| CYP2E1 |
| GCH1 |
| ELAC2 |
| MAOB |
| PRDX6 |
| CYP1A1 |
| PINK1 |
| GPX3 |
| ACADL |
| SP1 |
| NOX4 |
| CASP9 |
| F2 |
| ETFA |
| PPARG |
| HTRA2 |
| ADIPOQ |
| CYP3A4 |
| ALDH2 |
| FOXO3 |
| COX5A |
| SELENON |
| OGG1 |
| KNG1 |
| MTOR |
| CHAT |
| ABCD1 |
| MTO1 |
| TLR4 |
| BAX |
| PRDX2 |
| POLG |
| MAPK3 |
| VCAM1 |
| HSF1 |
| NCF2 |
| PRNP |
| FOS |
| MAOA |
| APOE |
| HIF1A |
| CACNA1C |
| CASP8 |
| LMNA |
| XBP1 |
| CRH |
| PPARGC1A |
| CAV1 |
| PON2 |
| BCL2 |
| TARDBP |
| MAPK10 |
| HSPA1A |
| ACE |
| APEX1 |
| OLR1 |
| ESR1 |
| MAP3K5 |
| VCP |
| AIFM1 |
| ICAM1 |
| CP |
| SCN5A |
| HSPB1 |
| BDNF |
| NFKB1 |
| PRDX5 |
| HBG2 |
| CAV3 |
| TYR |
| KEAP1 |
| GSTM1 |
| AARS2 |
| FOXO1 |
| PSEN1 |
| DDIT3 |
| GSTP1 |
| CPT1A |
| SLC25A20 |
| GPX1 |
| SQSTM1 |
| ETFDH |
| HSPA5 |
| ACOX1 |
| CYP2D6 |
| VWF |
| NDUFS4 |
| GAPDH |
| MAPT |
| PARP1 |
| VEGFA |
| RYR2 |
| CCL2 |
| HADHB |
| RYR1 |
| CYBB |
| GFM1 |
| HSPA4 |
| PTGS2 |
| SNCA |
| IFNG |
| NQO1 |
| HSP90AA1 |
| EDN1 |
| TGFB1 |
| JUN |
| CYBA |
| CXCL8 |
| CRP |
| ALB |
| SIRT1 |
| ACADS |
| HADH |
| AKT1 |
| INS |
| IL10 |
| ACADVL |
| TXN |
| CASP3 |
| G6PD |
| MAPK1 |
| SLC6A4 |
| ACADM |
| IL1B |
| CYCS |
| MAPK8 |
| HADHA |
| PRKN |
| IL6 |
| PON1 |
| PARK7 |
| GSR |
| XDH |
| APP |
| MAPK14 |
| MPO |
| SOD2 |
| CPT2 |
| NFE2L2 |
| TP53 |
| NOS1 |
| HMOX1 |
| TNF |
| CAT |
| NOS2 |
| SOD1 |
| NOS3 |
