# Supplementary material for: Long non-coding RNA NMRAL2P promotes glycolysis and reduces ROS in head and neck tumors by interacting with the ENO1 protein and promoting GPX2 transcription
Source: PeerJ. 2023 Oct 2;11:e16140. doi: 10.7717/peerj.16140 (PMC10552744; doi:10.7717/peerj.16140)
Supplement: Supplemental Information 8 — (A–B) After TU177 and AMC-HN-8 cells transfected into NMRAL2P-oe,NMRAL2P-ASO and the corresponding negative control groups Vector (pcDNA3.1), NC (NC-ASO), the changes of ENO1 protein were compared by western blot after 12 h with and without cycloheximide.(CHX: cycloheximide) [file peerj-11-16140-s008.pdf]

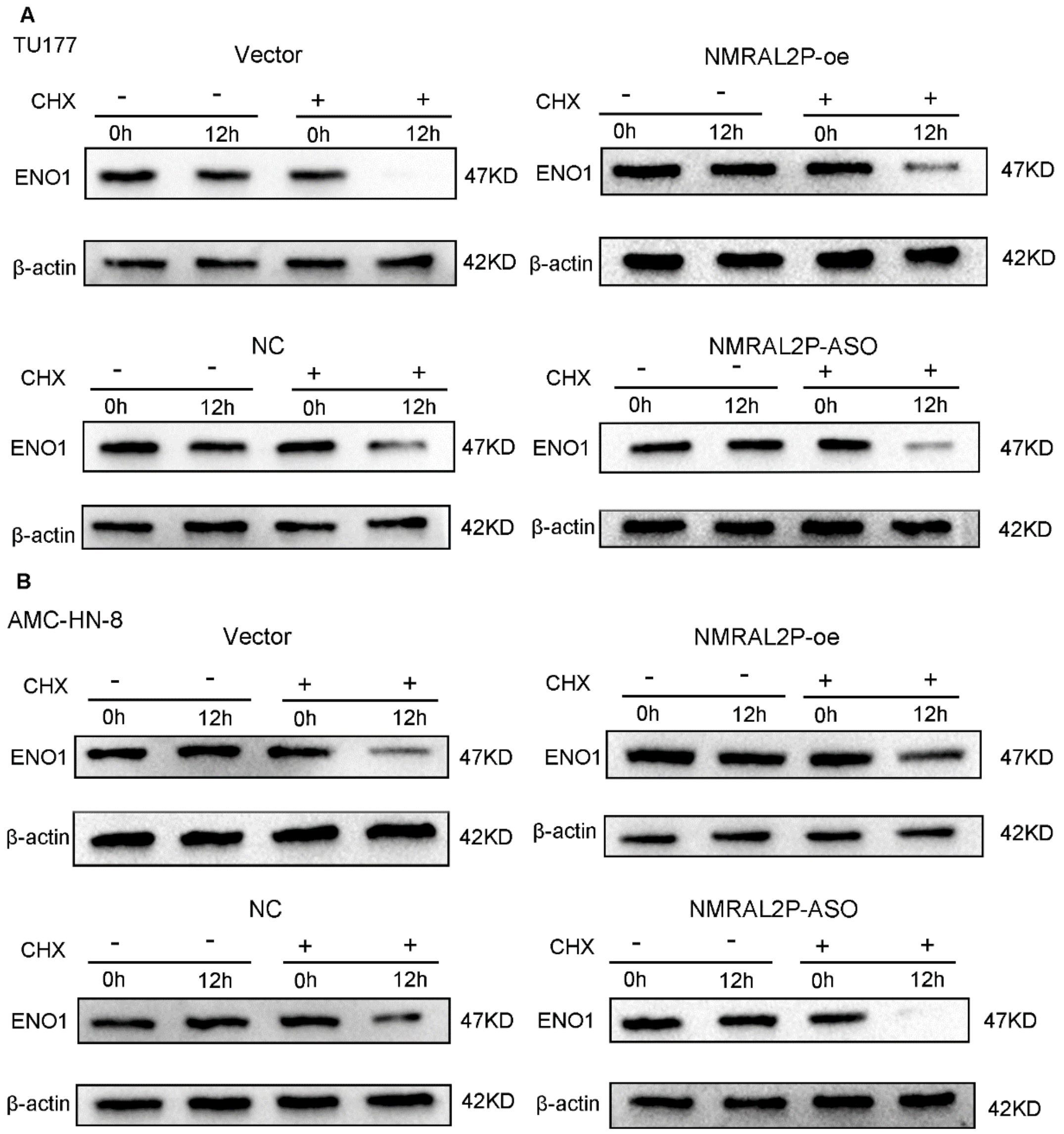

Supplementary Figure S2: Verification of the effectiveness of cycloheximide. (A-B): After TU177 and AMC-HN-8 cells transfected into NMRAL2P-oe,NMRAL2P-ASO and the corresponding negative control groups Vector (pcDNA3.1), NC (NC-ASO), the changes of ENO1 protein were compared by western blot after 12 hours with and without cycloheximide.(CHX: cycloheximide)
