# Supplementary material for: Long non-coding RNA NMRAL2P promotes glycolysis and reduces ROS in head and neck tumors by interacting with the ENO1 protein and promoting GPX2 transcription
Source: PeerJ. 2023 Oct 2;11:e16140. doi: 10.7717/peerj.16140 (PMC10552744; doi:10.7717/peerj.16140)
Supplement: Supplemental Information 9 — (A–B) The changes of cell viability of TU177 and AMC-HN-8 after transferred into shENO1 and shGPX2 were verified by MTS assay. (C–D) the changes of cell migration and invasion ability of TU177 and AMC-HN-8 after transferred into shENO1 and shGPX2 were verified by transwell assay. (E–F) the changes of proliferation activity of TU177 and AMC-HN-8 after transferred into shENO1 and shGPX2 were verified by clone formation assay. (G) The changes of lactate levels in TU177 and AMC-HN-8 cells after transferred into shENO1 were verified by lactic acid kit. (H) The changes of ROS yield, SOD activity and MAD content in TU177 and AMC-HN-8 cells after transferred into shGPX2 were verified. (ROS, Reactive Oxygen Species; SOD, Superoxide Dismutase; MAD, Malondialdehyde) [file peerj-11-16140-s009.pdf]

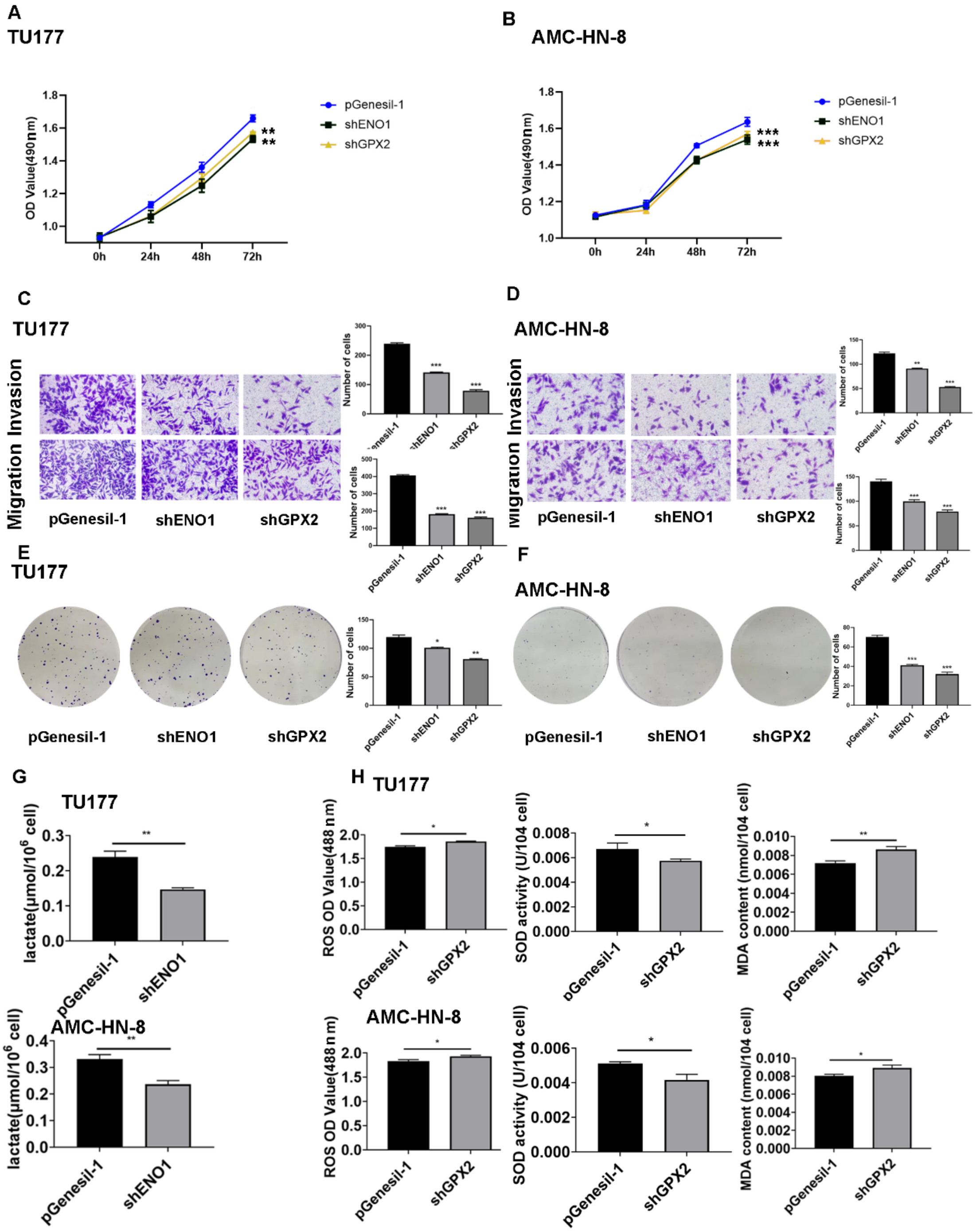

Supplementary Figure S3:ENO1 and GPX2 knock down function assay.(A-B):The changes of cell viability of TU177 and AMC-HN-8 after transferred into shENO1 and shGPX2 were verified by MTS assay.(C-D):the changes of cell migration and invasion ability of TU177 and AMC-HN-8 after transferred into shENO1 and shGPX2 were verified by transwell assay.(E-F):the changes of proliferation activity of TU177 and AMC-HN-8 after transferred into shENO1 and shGPX2 were verified by clone formation assay.(G): The changes of lactate levels in TU177 and AMC-HN-8 cells after transferred into shENO1 were verified by lactic acid kit. (H): The changes of ROS yield,

SOD activity and MAD content in TU177 and AMC-HN-8 cells after transferred into shGPX2 were verified. (ROS: Reactive Oxygen Species,SOD: Superoxide Dismutase,MAD:Malondialdehyde)
