# Supplementary material for: Long non-coding RNA NMRAL2P promotes glycolysis and reduces ROS in head and neck tumors by interacting with the ENO1 protein and promoting GPX2 transcription
Source: PeerJ. 2023 Oct 2;11:e16140. doi: 10.7717/peerj.16140 (PMC10552744; doi:10.7717/peerj.16140)
Supplement: Supplemental Information 10 — (A–F) MTS, Transwell, and colony formation experiments showed that Tempol partially enhanced the reduced migration, invasion, and proliferation ability of TU177 and AMC-HN-8 cells induced by GPX2 knockdown. (G–H) In TU177 and AMC-HN-8 cells, Tempol partially attenuated the enhancement of oxidative stress induced by shGPX2 knockout. (ROS, Reactive Oxygen Species; SOD, Superoxide Dismutase; MAD, Malondialdehyde; Tempol, A superoxide dismutase analogue that effectively neutralizes reactive oxygen species) [file peerj-11-16140-s010.pdf]

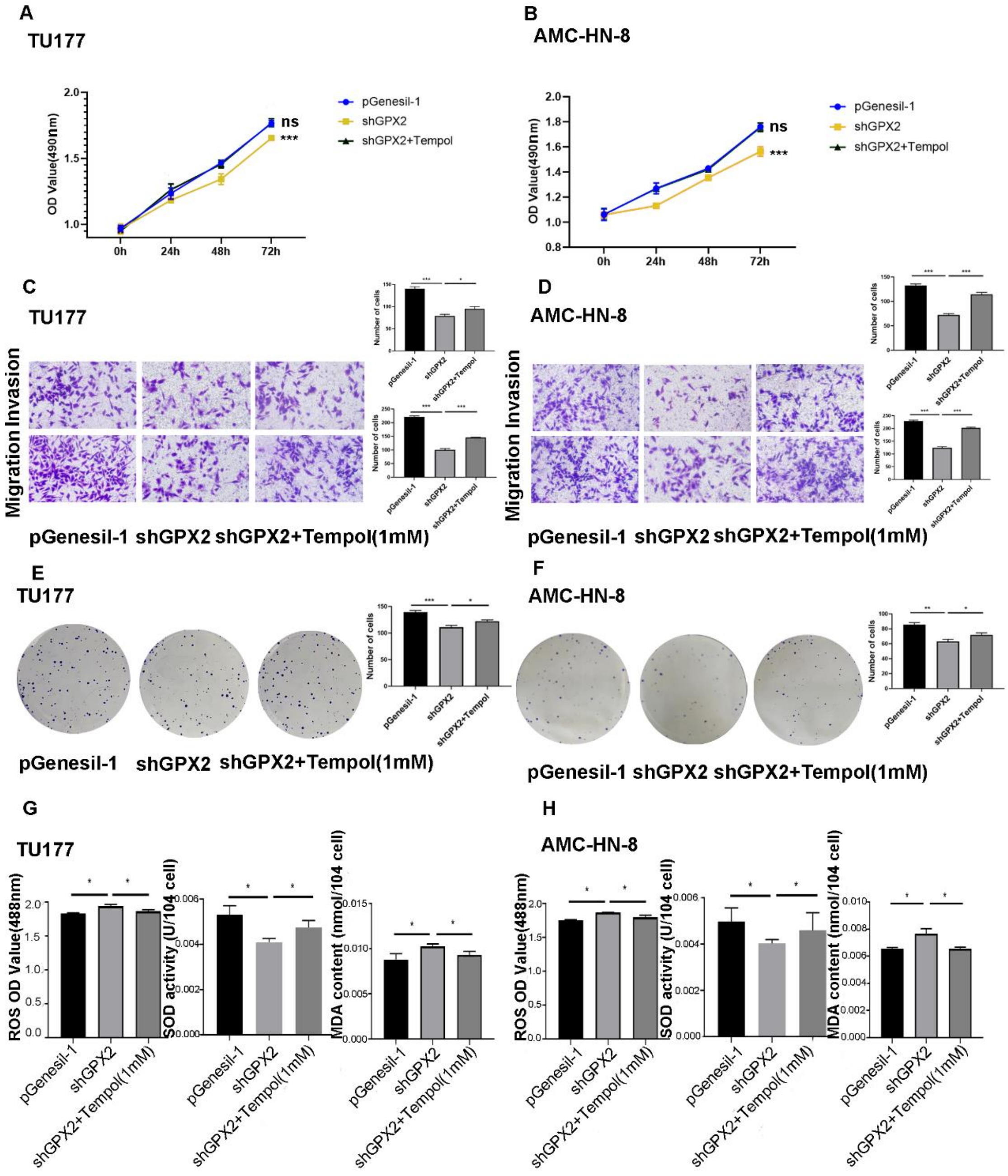

Supplementary Figure S4: Functional recovery assays.(A-F): MTS, Transwell, and colony formation experiments showed that Tempol partially enhanced the reduced migration, invasion, and proliferation ability of TU177 and AMC-HN-8 cells induced by GPX2 knockdown. (G-H): In TU177 and AMC-HN-8 cells, Tempol partially attenuated the enhancement of oxidative stress induced by shGPX2 knockout. (ROS: Reactive Oxygen Species,SOD: Superoxide Dismutase,MAD:Malondialdehyde, Tempol: A superoxide dismutase analogue that effectively neutralizes reactive oxygen species)
