# Supplementary material for: Long non-coding RNA NMRAL2P promotes glycolysis and reduces ROS in head and neck tumors by interacting with the ENO1 protein and promoting GPX2 transcription
Source: PeerJ. 2023 Oct 2;11:e16140. doi: 10.7717/peerj.16140 (PMC10552744; doi:10.7717/peerj.16140)
Supplement: Supplemental Information 11 [file peerj-11-16140-s011.pdf]

10 20 30 40 50 60

TTTAAACTTAAGCTTGGTACCGAGCTCGGATCCATAATTCTCACCAGAGAAGAAACACAGCAT

211016HE1007-2.YE8451F-XO.CMV-F_A02.ab1(40>600) →

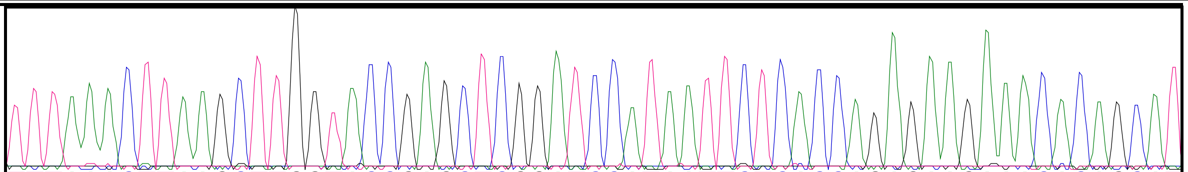

TTTAAACTTAAGCTTGGTACCGAGCTCGGATCCATAATTCTCACCAGAGAAGAAACACAGCAT

YE8451F ABX16537.seq(1>1825) → GGATCCATAATTCTCACCAGAGAAGAAACACAGCAT

70 80 90 100 110 120

TTCGGCTGGTGTCGCTGCTCCAGGGAAAGTTCTGTTACTCCACTGACTCTCTCTTTTCCTGAT

211016HE1007-2.YE8451F-XO.CMV-F_A02.ab1(40>600) →

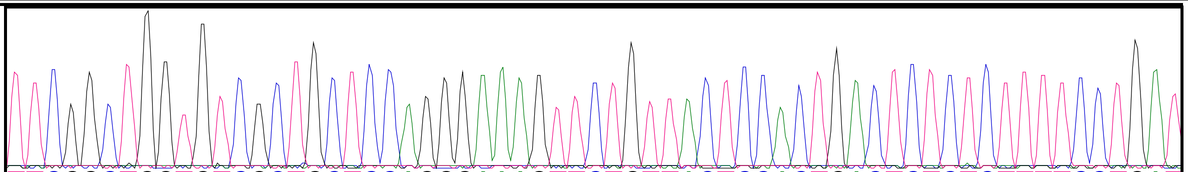

TTCGGCTGGTGTCGCTGCTCCAGGGAAAGTTCTGTTACTCCACTGACTCTCTCTTTTCCTGAT

YE8451F ABX16537.seq(1>1825) → TTCGGCTGGTGTCGCTGCTCCAGGGAAAGTTCTGTTACTCCACTGACTCTCTCTTTTCCTGAT

130 140 150 160 170 180

AACATGGCCAGCAAGAAAGTAATTACAGTGTTTGGAGCAACAGGGGAAGCTGGTGGCAGACTC

211016HE1007-2.YE8451F-XO.CMV-F_A02.ab1(40>600) →

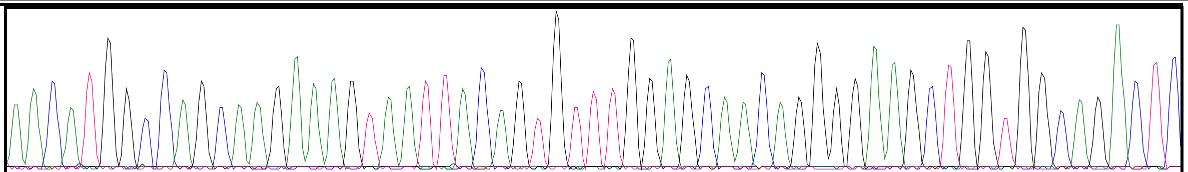

AACATGGCCAGCAAGAAAGTAATTACAGTGTTTGGAGCAACAGGGGAAGCTGGTGGCAGACTC

YE8451F ABX16537.seq(1>1825) → AACATGGCCAGCAAGAAAGTAATTACAGTGTTTGGAGCAACAGGGGAAGCTGGTGGCAGACTC

190 200 210 220 230 240 250

CGCCAAGCACCTGGGTCTGAAGCACGTGGTGTACAGCGGCCTGGAGAACGTCAAGCGACTGAC

211016HE1007-2.YE8451F-XO.CMV-F_A02.ab1(40>600) →

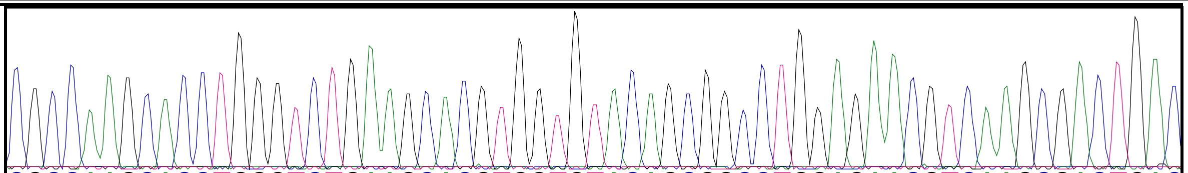

CGCCAAGCACCTGGGTCTGAAGCACGTGGTGTACAGCGGCCTGGAGAACGTCAAGCGACTGAC

YE8451F ABX16537.seq(1>1825) → CGCCAAGCACCTGGGTCTGAAGCACGTGGTGTACAGCGGCCTGGAGAACGTCAAGCGACTGAC

260 270 280 290 300 310

GGATGGCAAGCTGGAGGTGCCGCACTTTGACAGCAAGGGCGAGGTGGAGGAGTACTTCTGGTC

211016HE1007-2.YE8451F-XO.CMV-F_A02.ab1(40>600) →

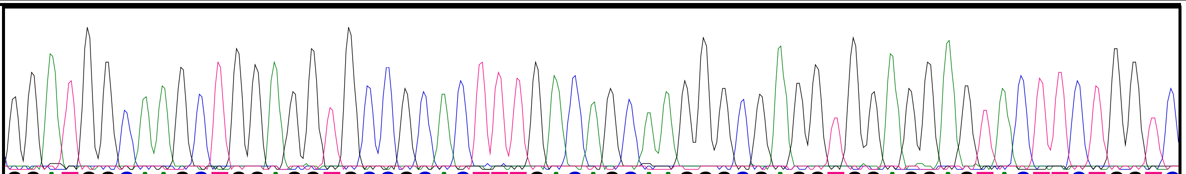

GGATGGCAAGCTGGAGGTGCCGCACTTTGACAGCAAGGGCGAGGTGGAGGAGTACTTCTGGTC

YE8451F ABX16537.seq(1>1825) → GGATGGCAAGCTGGAGGTGCCGCACTTTGACAGCAAGGGCGAGGTGGAGGAGTACTTCTGGTC

320 330 340 350 360 370

CATTGGCATCCCCATGACCAGTGTCCGCGTGGCGGCCTACTTTGAAAACTTTCTCGCGGCGTG

211016HE1007-2.YE8451F-XO.CMV-F_A02.ab1(40>600) →

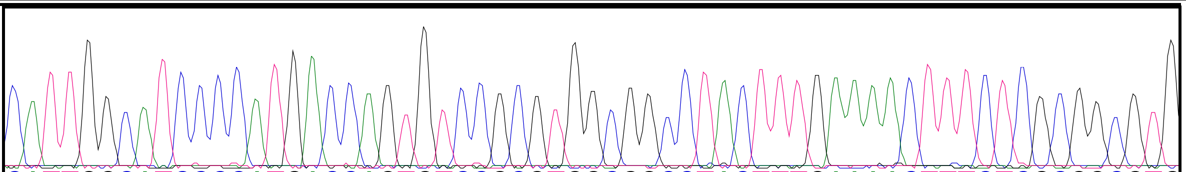

CATTGGCATCCCCATGACCAGTGTCCGCGTGGCGGCCTACTTTGAAAACTTTCTCGCGGCGTG

YE8451F ABX16537.seq(1>1825) → CATTGGCATCCCCATGACCAGTGTCCGCGTGGCGGCCTACTTTGAAAACTTTCTCGCGGCGTG

380 390 400 410 420 430 440

GCGGCCCGTGAAAGCCTCTGATGGAGATTACTACACCTTGGCTGTACCGATGGGAGATGTACC

211016HE1007-2.YE8451F-XO.CMV-F_A02.ab1(40>600) →

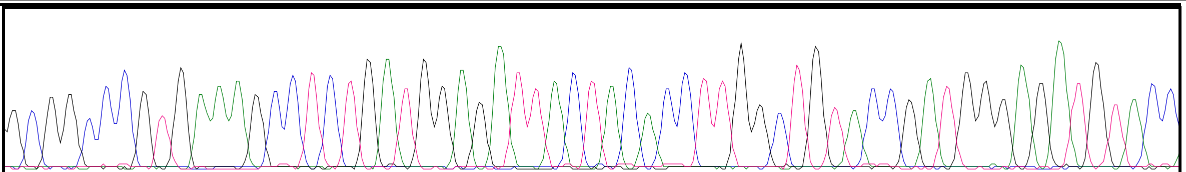

GCGGCCCGTGAAAGCCTCTGATGGAGATTACTACACCTTGGCTGTACCGATGGGAGATGTACC

YE8451F ABX16537.seq(1>1825) → GCGGCCCGTGAAAGCCTCTGATGGAGATTACTACACCTTGGCTGTACCGATGGGAGATGTACC

450 460 470 480 490 500

AATGGATGGTATCTCTGTTGCTGATATTGGAGCAGCCGTCTCTAGCATTTTTAATTCTCCAGA

211016HE1007-2.YE8451F-XO.CMV-F_A02.ab1(40>600) →

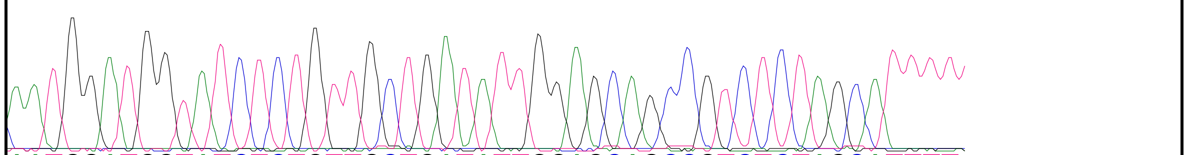

AATGGATGGTATCTCTGTTGCTGATATTGGAGCAGCCGTCTCTAGCATTTT

YE8451F ABX16537.seq(1>1825) → AATGGATGGTATCTCTGTTGCTGATATTGGAGCAGCCGTCTCTAGCATTTTTAATTCTCCAGA

450 460 470 480 490 500

AATGGATGGTATCTCTGTTGCTGATATTGGAGCAGCCGTCTCTAGCATTTTTAATTCTCCAGA

211016HE1007-2.YE8451F-XO.YE8451F1_C02.ab1(40>600)→

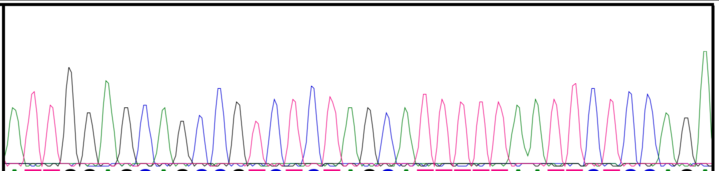

ATTGGAGCAGCCGTCTCTAGCATTTTTAATTCTCCAGA

510 520 530 540 550 560

GGAATTTTTAGGCAAGGCCGTGGGGCTCAGTGCAGAAGCACTAACAATACAGCAATATGCTGA

YE8451F ABX16537.seq(1>1825) → GGAATTTTTAGGCAAGGCCGTGGGGCTCAGTGCAGAAGCACTAACAATACAGCAATATGCTGA

211016HE1007-2.YE8451F-XO.YE8451F1_C02.ab1(40>600)→

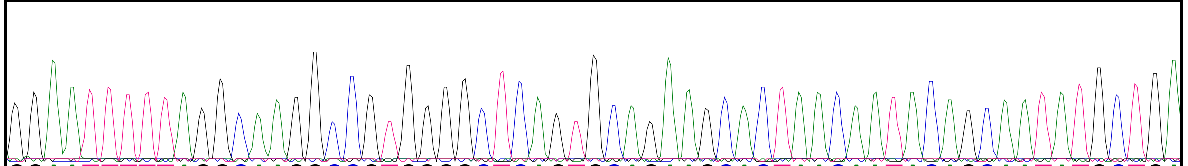

GGAATTTTTAGGCAAGGCCGTGGGGCTCAGTGCAGAAGCACTAACAATACAGCAATATGCTGA

570 580 590 600 610 620 630

TGTTTTGTCCAAGGTTTTGGGGAAAGAAGTCCGAGATGCAAAGATTACCCCGGAAGCTTTCGA

YE8451F ABX16537.seq(1>1825) → TGTTTTGTCCAAGGTTTTGGGGAAAGAAGTCCGAGATGCAAAGATTACCCCGGAAGCTTTCGA

211016HE1007-2.YE8451F-XO.YE8451F1_C02.ab1(40>600)→

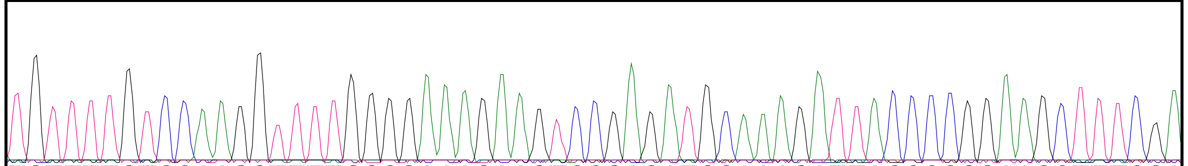

TGTTTTGTCCAAGGTTTTGGGGAAAGAAGTCCGAGATGCAAAGATTACCCCGGAAGCTTTCGA

640 650 660 670 680 690

GAAGCTGGGATTCCCTGCAGCAAAGGAAATAGCCAATATGTGTCGTTTCTATGAAATGAAGCC

YE8451F ABX16537.seq(1>1825) → GAAGCTGGGATTCCCTGCAGCAAAGGAAATAGCCAATATGTGTCGTTTCTATGAAATGAAGCC

211016HE1007-2.YE8451F-XO.YE8451F1_C02.ab1(40>600)→

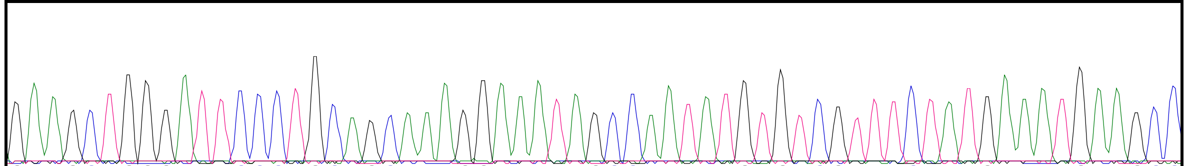

GAAGCTGGGATTCCCTGCAGCAAAGGAAATAGCCAATATGTGTCGTTTCTATGAAATGAAGCC

700 710 720 730 740 750

AGACCGAGATGTCAATCTCACCCACCAACTAAATCCCAAAGTCAAAAGCTTCAGCCAGTTTAT

YE8451F ABX16537.seq(1>1825) → AGACCGAGATGTCAATCTCACCCACCAACTAAATCCCAAAGTCAAAAGCTTCAGCCAGTTTAT
211016HE1007-2.YE8451F-XO.YE8451F1_C02.ab1(40>600) →

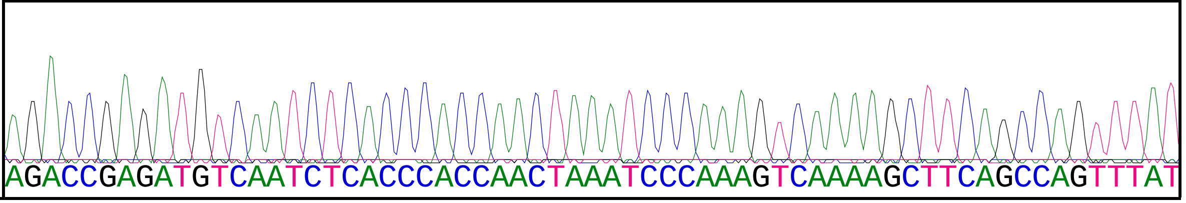

760 770 780 790 800 810

CTCAGAGAACCAGGGAGCCTTCAAGGGCATGTAGAAAATCAGCTGTTCAGATAGGCCTCTGCA

YE8451F ABX16537.seq(1>1825) → CTCAGAGAACCAGGGAGCCTTCAAGGGCATGTAGAAAATCAGCTGTTCAGATAGGCCTCTGCA
211016HE1007-2.YE8451F-XO.YE8451F1_C02.ab1(40>600) →

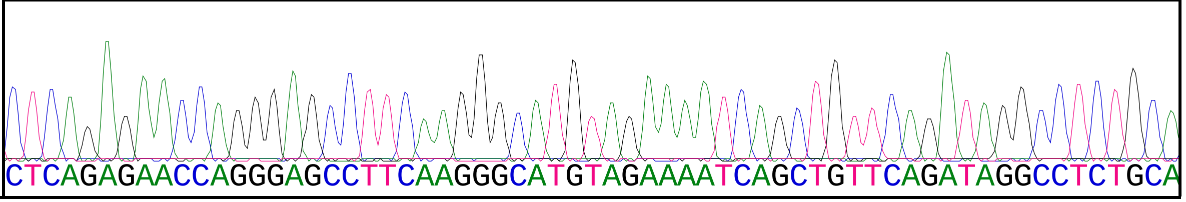

820 830 840 850 860 870 880

CCACACAGCCTCTTTCCTCTCTGATCCTTTTCCTCTTTACGGCACAACATTCATGTTGACAGA

YE8451F ABX16537.seq(1>1825) → CCACACAGCCTCTTTCCTCTCTGATCCTTTTCCTCTTTACGGCACAACATTCATGTTGACAGA
211016HE1007-2.YE8451F-XO.YE8451F1_C02.ab1(40>600) →

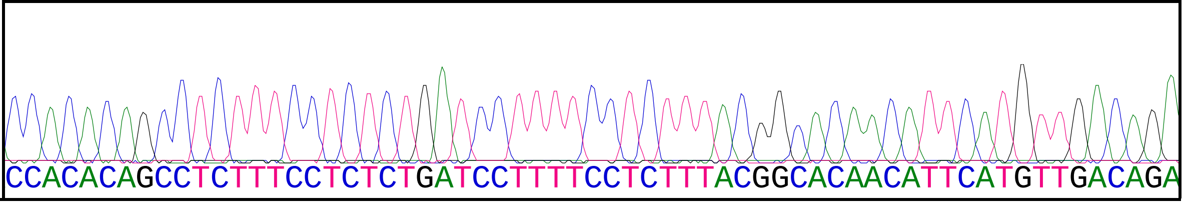

890 900 910 920 930 940

ACATGCTGGAATGCAATTGTTTGCAACACCGAAGGATTTCCTGCGGTCGCCTCTTCAGTAGGA

YE8451F ABX16537.seq(1>1825) → ACATGCTGGAATGCAATTGTTTGCAACACCGAAGGATTTCCTGCGGTCGCCTCTTCAGTAGGA
211016HE1007-2.YE8451F-XO.YE8451F1_C02.ab1(40>600) →

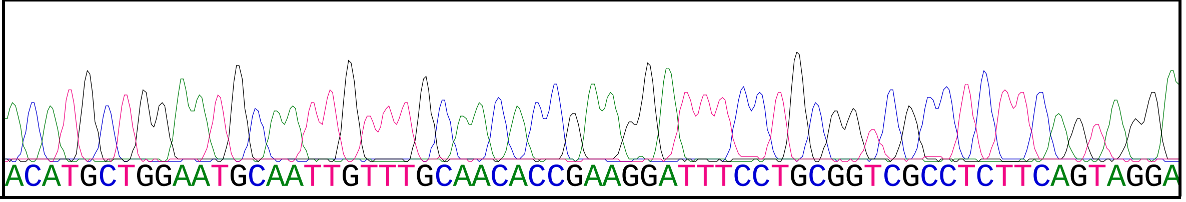

890 900 910 920 930 940

ACATGCTGGAATGCAATTGTTTGCAACACCGAAGGATTTCCTGCGGTCGCCTCTTCAGTAGGA

211016HE1007-2.YE8451F-XO.YE8451F2_D02.ab1(40>600)→

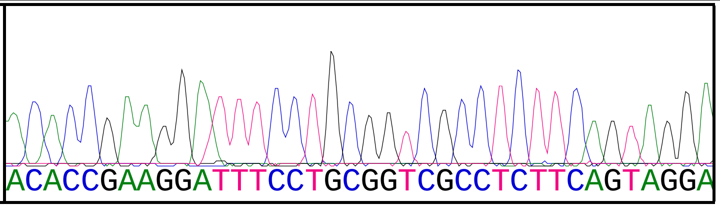

950 960 970 980 990 1000

AGCACTGCATTGGTGATAGAACACGGTAATTTGATTCACATTTAACTTGCTAGTTAGTGATAA

YE8451F ABX16537.seq(1>1825) → AGCACTGCATTGGTGATAGAACACGGTAATTTGATTCACATTTAACTTGCTAGTTAGTGATAA

211016HE1007-2.YE8451F-XO.YE8451F1_C02.ab1(40>600)→

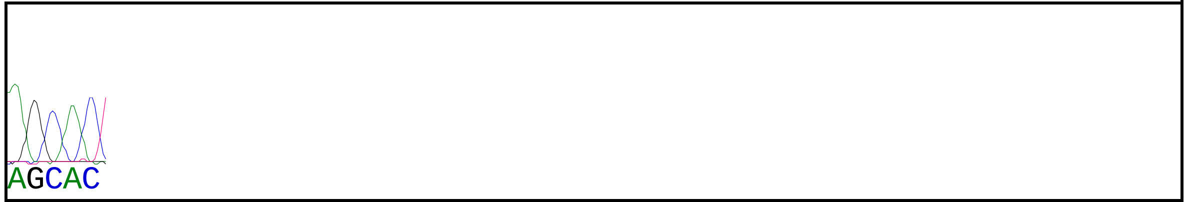

211016HE1007-2.YE8451F-XO.YE8451F2_D02.ab1(40>600)→

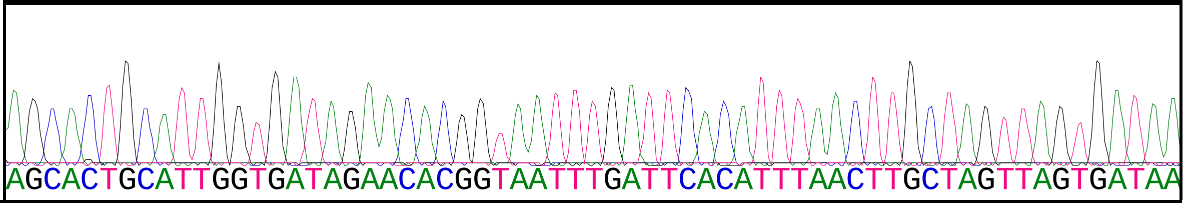

1010 1020 1030 1040 1050 1060 1070

GGGTGGTACACCTGTTTGGTAAAATGAGAAGCCTCGGAACTTGGAGCTTCTCTCCTACCACTA

YE8451F ABX16537.seq(1>1825) → GGGTGGTACACCTGTTTGGTAAAATGAGAAGCCTCGGAACTTGGAGCTTCTCTCCTACCACTA

211016HE1007-2.YE8451F-XO.YE8451F2_D02.ab1(40>600)→

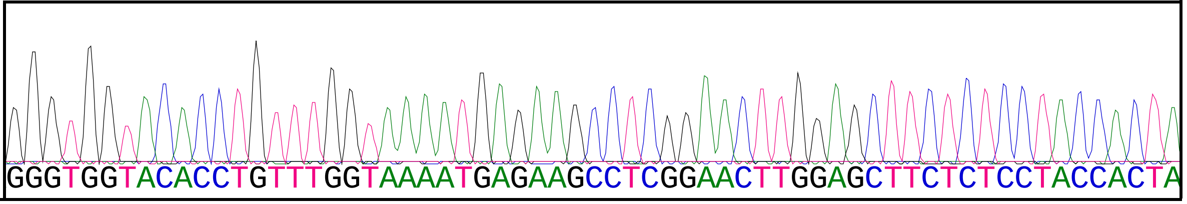

1080 1090 1100 1110 1120 1130

ATGGGAGGGCAGATTATACTGGGATTTCTCCTGGGTGAGTAATTTCAAGCCCTAATGCTGAAA

YE8451F ABX16537.seq(1>1825) → ATGGGAGGGCAGATTATACTGGGATTTCTCCTGGGTGAGTAATTTCAAGCCCTAATGCTGAAA

1080 1090 1100 1110 1120 1130
ATGGGAGGGCAGATTATACTGGGATTTCTCCTGGGTGAGTAATTTCAAGCCCTAATGCTGAAA

211016HE1007-2.YE8451F-XO.YE8451F2_D02.ab1(40>600)→

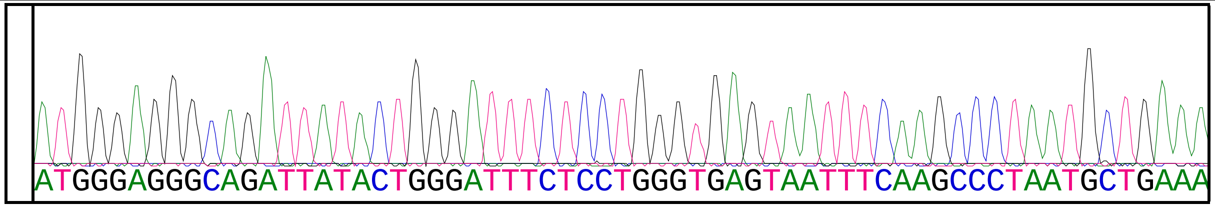

1140 1150 1160 1170 1180 1190
TTCCCCTAGGCAGCTCCAGTTTTCTCAACTGCATTGCAAAATTCCCAGTGAACTTTTAAGTAC

YE8451F ABX16537.seq(1>1825)→ TTCCCCTAGGCAGCTCCAGTTTTCTCAACTGCATTGCAAAATTCCCAGTGAACTTTTAAGTAC
211016HE1007-2.YE8451F-XO.YE8451F2_D02.ab1(40>600)→

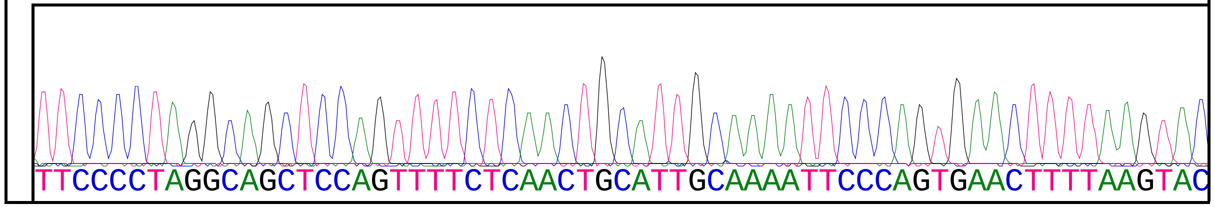

1200 1210 1220 1230 1240 1250 1260
TTTTAACTTAAAAAAAATGAACATCTTTGTAGAGAATTTTCTGGGGAACATGGTGTTCAATGAA

YE8451F ABX16537.seq(1>1825)→ TTTTAACTTAAAAAAAATGAACATCTTTGTAGAGAATTTTCTGGGGAACATGGTGTTCAATGAA
211016HE1007-2.YE8451F-XO.YE8451F2_D02.ab1(40>600)→

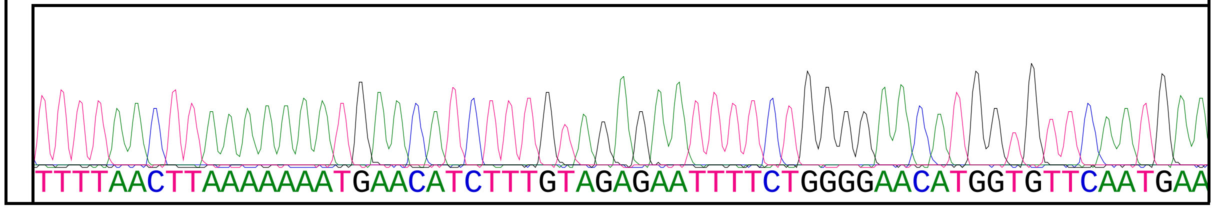

1270 1280 1290 1300 1310 1320
CAAGCACAAGCATTGGAAATGCTAAAATTCAGTTTTGCCTCAAGATTGGAAGTTTATTTTCTG

YE8451F ABX16537.seq(1>1825)→ CAAGCACAAGCATTGGAAATGCTAAAATTCAGTTTTGCCTCAAGATTGGAAGTTTATTTTCTG
211016HE1007-2.YE8451F-XO.YE8451F2_D02.ab1(40>600)→

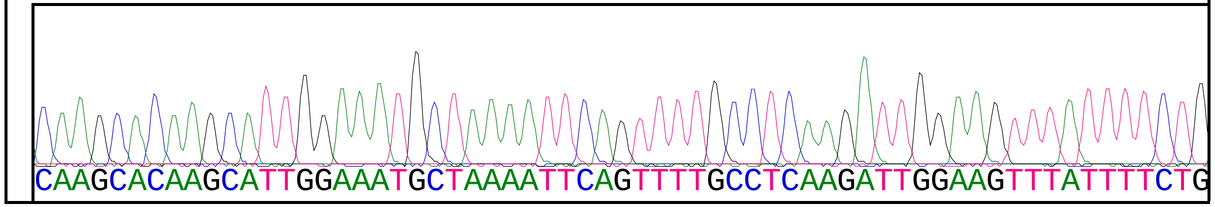

1330 1340 1350 1360 1370 1380

ACTCATTCATGAAGTCATCTATTGAGCCACCATTCAATTATTCATCTATTAATTCCTTGATCC

YE8451F ABX16537.seq(1>1825) → ACTCATTCATGAAGTCATCTATTGAGCCACCATTCAATTATTCATCTATTAATTCCTTGATCC

211016HE1007-2.YE8451F-XO.YE8451F2_D02.ab1(40>600) →

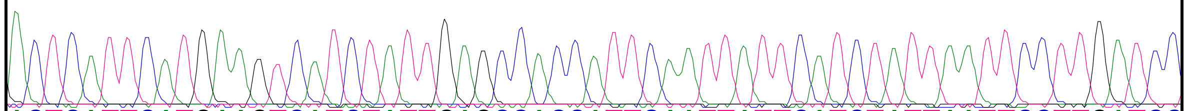

ACTCATTCATGAAGTCATCTATTGAGCCACCATTCAATTATTCATCTATTAATTCCTTGATCC

1390 1400 1410 1420 1430 1440

TTCATTTATCCATTCTGCAAACTTTTCTTGAGCACCAGCACGGGTGGCCATTTGTGGACTTCT

YE8451F ABX16537.seq(1>1825) → TTCATTTATCCATTCTGCAAACTTTTCTTGAGCACCAGCACGGGTGGCCATTTGTGGACTTCT

211016HE1007-2.YE8451F-XO.YE8451F2_D02.ab1(40>600) →

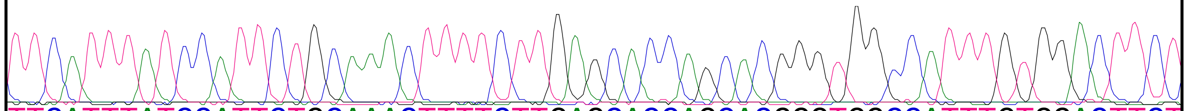

TTCATTTATCCATTCTGCAAACTTTTCTTGAGCACCAGCACGGGTGGCCATTTGTGGACTTCT

211016HE1007-2.YE8451F-XO.YE8451F3_A02.ab1(40>600) →

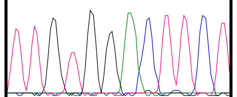

TTGTGGACTTCT

1450 1460 1470 1480 1490 1500 1510

CTTCATTCCTATGTGTTTTCTTATCAAAGTGATCCACTCTCGAAAGGCTCCTTTCCAGTCTGT

YE8451F ABX16537.seq(1>1825) → CTTCATTCCTATGTGTTTTCTTATCAAAGTGATCCACTCTCGAAAGGCTCCTTTCCAGTCTGT

211016HE1007-2.YE8451F-XO.YE8451F2_D02.ab1(40>600) →

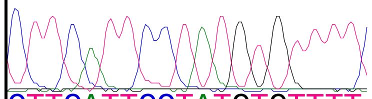

CTTCATTCCTATGTGTTTT

211016HE1007-2.YE8451F-XO.YE8451F3_A02.ab1(40>600) →

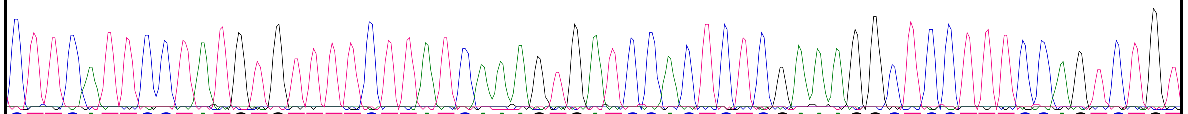

CTTCATTCCTATGTGTTTTCTTATCAAAGTGATCCACTCTCGAAAGGCTCCTTTCCAGTCTGT

1520 1530 1540 1550 1560 1570
GGTTGGGTTCAAGTCATGCCAGGGCCAGGGGGCCCATCTCCTCGTTTAGCTCTAGGCAAAATC

YE8451F ABX16537.seq(1>1825) → GGTTGGGTTCAAGTCATGCCAGGGCCAGGGGGCCCATCTCCTCGTTTAGCTCTAGGCAAAATC
211016HE1007-2.YE8451F-XO.YE8451F3_A02.ab1(40>600) →

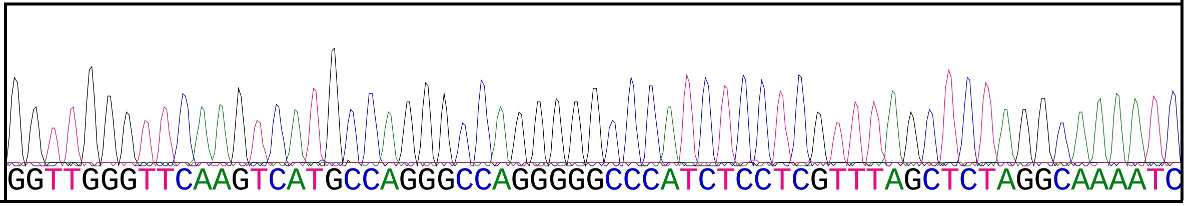

1580 1590 1600 1610 1620 1630
CAGGGGATCTGCAGTGGGGAGCGGGGGCAGGAAGCTGGAGGGAAGGCCTGTGAAGGGTAGGGA

YE8451F ABX16537.seq(1>1825) → CAGGGGATCTGCAGTGGGGAGCGGGGGCAGGAAGCTGGAGGGAAGGCCTGTGAAGGGTAGGGA
211016HE1007-2.YE8451F-XO.YE8451F3_A02.ab1(40>600) →

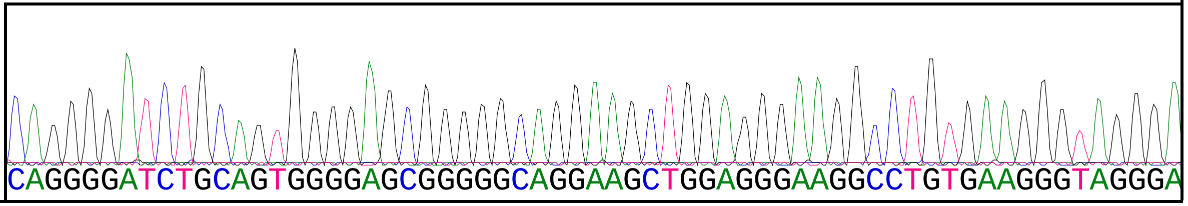

1640 1650 1660 1670 1680 1690 1700
TGTGGAAAGACAAGGTGACAGAAGGACCCAATAGGACCTTTCTATATCTCTGGCTTAGCATTT

YE8451F ABX16537.seq(1>1825) → TGTGGAAAGACAAGGTGACAGAAGGACCCAATAGGACCTTTCTATATCTCTGGCTTAGCATTT
211016HE1007-2.YE8451F-XO.YE8451F3_A02.ab1(40>600) →

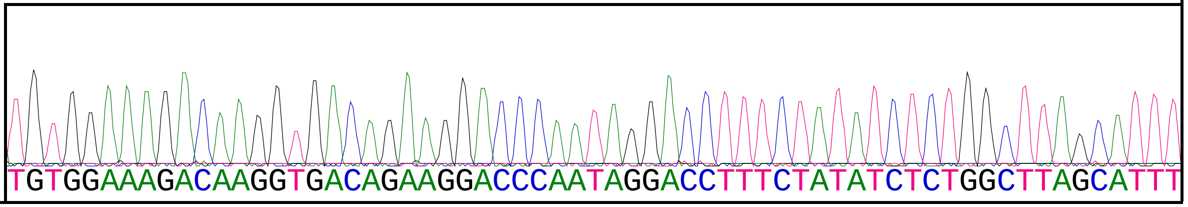

1710 1720 1730 1740 1750 1760
TCTACATCATATTGTAATCGTCTTATTTGCTAGTTTTCTTCCTTACTGTGAGTGACTAACAGT

YE8451F ABX16537.seq(1>1825) → TCTACATCATATTGTAATCGTCTTATTTGCTAGTTTTCTTCCTTACTGTGAGTGACTAACAGT
211016HE1007-2.YE8451F-XO.YE8451F3_A02.ab1(40>600) →

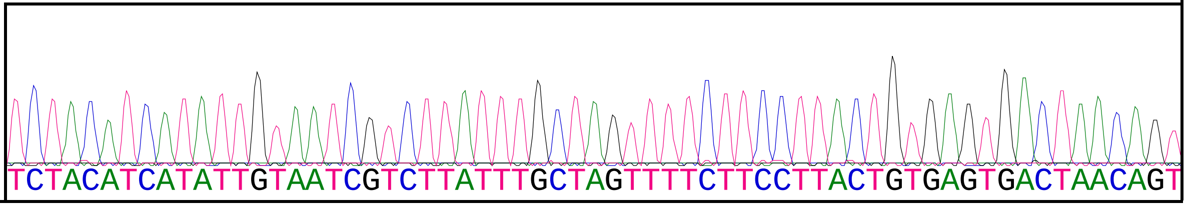

1770 1780 1790 1800 1810 1820

CATCTTTATCCCAGTGCCTGGTACATAATAAGTGATCAATAAATGTTGATTGACTAAATGAGT

YE8451F ABX16537.seq(1>1825) → CATCTTTATCCCAGTGCCTGGTACATAATAAGTGATCAATAAATGTTGATTGACTAAATGAGT

211016HE1007-2.YE8451F-XO.YE8451F3_A02.ab1(40>600) →

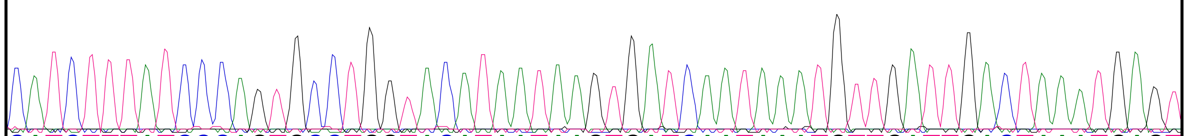

CATCTTTATCCCAGTGCCTGGTACATAATAAGTGATCAATAAATGTTGATTGACTAAATGAGT

1830 1840 1850 1860 1870

AAAAAAAAAAAAAAAAAAAGAATTCTGCAGATATCCAGCACAGTGGCG

YE8451F ABX16537.seq(1>1825) → AAAAAAAAAAAAAAAAAAAGAATTC

211016HE1007-2.YE8451F-XO.YE8451F3_A02.ab1(40>600) →

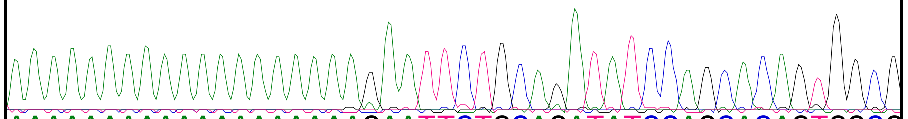

AAAAAAAAAAAAAAAAAAAGAATTCTGCAGATATCCAGCACAGTGGCG
