# Supplementary material for: Long non-coding RNA NMRAL2P promotes glycolysis and reduces ROS in head and neck tumors by interacting with the ENO1 protein and promoting GPX2 transcription
Source: PeerJ. 2023 Oct 2;11:e16140. doi: 10.7717/peerj.16140 (PMC10552744; doi:10.7717/peerj.16140)
Supplement: Supplemental Information 12 [file peerj-11-16140-s012.zip › Supplementary file 2/Target sequence of shGPX2 and shENO1.docx]

NMRAL2P-ASO：CCACTAATGGGAGGGCAGAT

shENO1: GAATGTCATCAAGGAGAAATA

shGPX2: CCGATCCCAAGCTCATCATTT
