# Supplementary material for: Long non-coding RNA NMRAL2P promotes glycolysis and reduces ROS in head and neck tumors by interacting with the ENO1 protein and promoting GPX2 transcription
Source: PeerJ. 2023 Oct 2;11:e16140. doi: 10.7717/peerj.16140 (PMC10552744; doi:10.7717/peerj.16140)
Supplement: Supplemental Information 13 [file peerj-11-16140-s013.zip › Supplementary file 3/CoA of 1-434aa in pcDNA3.1.pdf]

AZENTA LIFE SCIENCES

# Certificate of Analysis

**Project Tracking #**
80-1048012668

**Quote #**
80-1048012668_R19

**Clone ID #**
AA47641-1/C915302

**Sequence Name**
1-434aa in pcDNA3.1

**Cloning Vector**
pcDNA3.1(+)

**Cloning Sites**
5' HindIII and 3' BamHI

**Insert Length**
1383

| QC Item | Specification | Results |
|---|---|---|
| Insert Sequence | Insert sequence results consistent with target | √ Pass |
| Vector Sequence | Flanking sequence consistent with expected | √ Pass |
| Open Reading Frame across junction | Correct and consistent with target | n/a |
| Restriction Digest | Expected fragment sizes observed | √ Pass |
| PCR amplification | Correct without non-specific bands | n/a |
| DNA Quantity and Quality | Actual yield (by $A_{260}$) | 4 ug |
| | Concentration (n/a if lyophilized) | n/a |
| | Purity ($A_{260/A280}$ = 1.8 - 2.0) | √ Pass |
| | # of tubes | 1 |
| | Matrix | TE (lyophilized) |
| Endotoxin Test | Verified, ＜0.1 EU/ug(endo-free preps only) | n/a |
| Appearance | Clear, no visible particles | √ Pass |
| Label | Correct and clear | √ Pass |
| Comments | | |

**Restriction Digest Test Results**

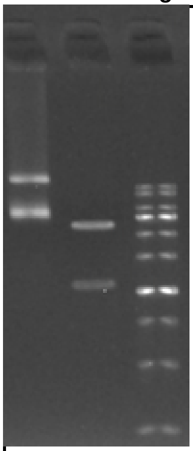

Lane Description( from left to right)
Lane 1: undigested
Lane 2: digested with SalI
Lane 3: ladder

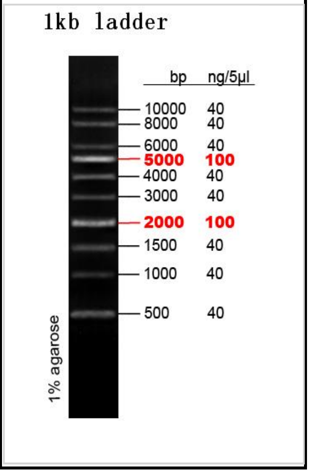


**Certified By**
Tianxing Ye

**Date**
11/05/2022
