# Supplementary material for: Long non-coding RNA NMRAL2P promotes glycolysis and reduces ROS in head and neck tumors by interacting with the ENO1 protein and promoting GPX2 transcription
Source: PeerJ. 2023 Oct 2;11:e16140. doi: 10.7717/peerj.16140 (PMC10552744; doi:10.7717/peerj.16140)
Supplement: Supplemental Information 13 [file peerj-11-16140-s013.zip › Supplementary file 3/CoA of 237-434 in pcDNA3.1.pdf]

Q-03-DR01-01

AZENTA LIFE SCIENCES

Certificate of Analysis

Project Tracking #	Quote #	Clone ID #
80-1048012668	80-1048012668_R19	AA47641-4/C913479

Sequence Name	Cloning Vector
237-434 in pcDNA3.1	pcDNA3.1(+)

Cloning Sites	Insert Length
5' HindIII and 3' BamHI	678

QC Item	Specification	Results
Insert Sequence	Insert sequence results consistent with target	√ Pass
Vector Sequence	Flanking sequence consistent with expected	√ Pass
Open Reading Frame across junction	Correct and consistent with target	n/a
Restriction Digest	Expected fragment sizes observed	√ Pass
PCR amplification	Correct without non-specific bands	n/a
DNA Quantity and Quality	Actual yield (by A_{260})	4 ug
	Concentration (n/a if lyophilized)	n/a
	Purity ($A_{260/A280}$ = 1.8 - 2.0)	√ Pass
	# of tubes	1
	Matrix	TE (lyophilized)
Endotoxin Test	Verified, ＜0.1 EU/ug(endo-free preps only)	n/a
Appearance	Clear, no visible particles	√ Pass
Label	Correct and clear	√ Pass
Comments		

Restriction Digest Test Results

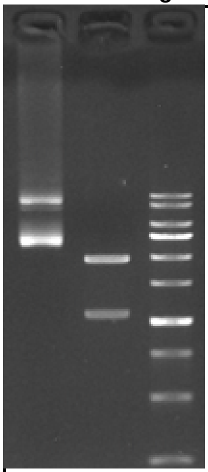

Lane Description(from left to right)
Lane 1: undigested
Lane 2: digested with SalI
Lane 3: ladder

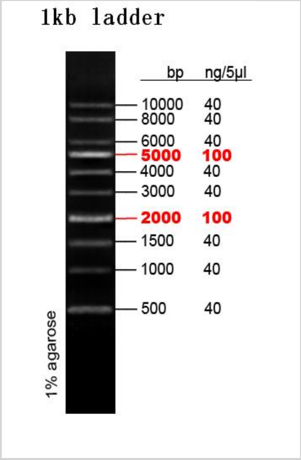

Certified By
Tianxing Ye

Date
10/30/2022
