# Supplementary material for: Long non-coding RNA NMRAL2P promotes glycolysis and reduces ROS in head and neck tumors by interacting with the ENO1 protein and promoting GPX2 transcription
Source: PeerJ. 2023 Oct 2;11:e16140. doi: 10.7717/peerj.16140 (PMC10552744; doi:10.7717/peerj.16140)
Supplement: Supplemental Information 13 [file peerj-11-16140-s013.zip › Supplementary file 3/CoA of 97-237aa in pcDNA3.1.pdf]

AZENTA LIFE SCIENCES

Certificate of Analysis

Project Tracking #
80-1048012668

Quote #
80-1048012668_R19

Clone ID #
AA47641-3/C911566

Sequence Name
97-237aa in pcDNA3.1

Cloning Vector
pcDNA3.1(+)

Cloning Sites
5' HindIII and 3' BamHI

Insert Length
504

QC Item	Specification	Results
Insert Sequence	Insert sequence results consistent with target	√ Pass
Vector Sequence	Flanking sequence consistent with expected	√ Pass
Open Reading Frame across junction	Correct and consistent with target	n/a
Restriction Digest	Expected fragment sizes observed	√ Pass
PCR amplification	Correct without non-specific bands	n/a
DNA Quantity and Quality	Actual yield (by A_{260})	4 ug
	Concentration (n/a if lyophilized)	n/a
	Purity ($A_{260/A280}$ = 1.8 - 2.0)	√ Pass
	# of tubes	1
	Matrix	TE (lyophilized)
Endotoxin Test	Verified, <0.1 EU/ug(endo-free preps only)	n/a
Appearance	Clear, no visible particles	√ Pass
Label	Correct and clear	√ Pass
Comments		

Restriction Digest Test Results

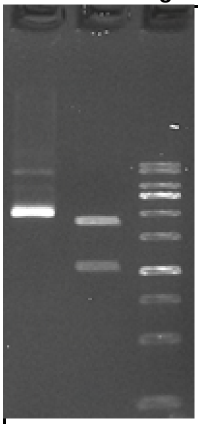

Lane Description(from left to right)
Lane 1: undigested
Lane 2: digested with SalI
Lane 3: ladder

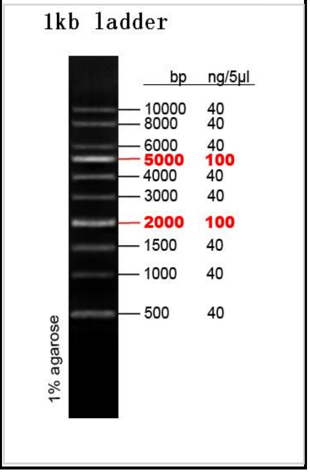

Certified By
Weiqin Shen

Date
10/27/2022
